# Differences in sleep spindles and polysomnography in humans: a meta-analysis on the influence of age, sex, and cognitive ability

**DOI:** 10.3389/frsle.2026.1802882

**Published:** 2026-04-14

**Authors:** Diana Campos-Beltrán, Shu Zhang, Lisa Marshall

**Affiliations:** 1 Institute of Experimental and Clinical Pharmacology and Toxicology, University of Lübeck, Lübeck, Germany; 2 University Hospital Schleswig Holstein, Lübeck, Germany; 3 Center of Brain, Behavior and Metabolism, University of Lübeck, Lübeck, Germany

**Keywords:** age, cognitive ability, EEG, polysomnography, sex, sleep, sleep spindles

## Abstract

This meta-analysis examines EEG sleep spindle and macrostructure differences in humans related to healthy aging, sex, and cognitive ability. Inclusion criteria required quantitative EEG data of healthy subjects, including sleep spindle properties and sleep polysomnography comparing younger to older subjects, females to males, and/or correlations with cognitive ability scores. The search included seven databases. The Mixed Methods Appraisal Tool (MMAT) calculated the study quality (risk of bias). Two meta-analyses used Hedges' g, and one averaged correlation (95% CI), all conducted with Meta-Essentials v1.4, with standard assessments of heterogeneity, publication bias, and meta-regression, supplemented by subgroup and sensitivity analyses. Results provide tables, forest plots, funnel plots, and bubble plots. *k* = 42 studies with *N* = 1,878 healthy subjects met our criteria. With age, sleep spindles decreased in amplitude, density, and duration. Sleep quality was reduced in older subjects showing shorter durations of both slow wave sleep (SWS) and rapid-eye movement (REM) sleep. Females revealed higher sleep spindle power (11–16 Hz), more prominent in older subjects; greater sleep efficiency, more total sleep time (TST), and longer SWS. Correlations between sleep properties and cognitive ability revealed age-dependent effects. Results yield key considerations in population comparisons and when targeting spindle activity, both for mechanistic research and for neuropsychiatric treatment. Yet further systematic investigations are warranted.

## 1 Introduction

There is a growing amount of insight into the mechanisms of sleep and its function regarding neuroplasticity (For instance: Buzsaki, 1989; Sejnowski and Destexhe, 2000; Sirota et al., 2003; Steriade and Timofeev, 2003; Genzel et al., 2014; Latchoumane et al., 2017; Mcdevitt et al., 2017; Antony et al., 2018; Marshall, 2018; Rothschild, 2018; Brodt et al., 2023). At the same time, the attempt to modulate brain function by influencing brain activity during sleep is an ongoing endeavor (Krone et al., 2025). Different techniques of weak electric stimulation like transcranial direct current stimulation (tDCS), transcranial alternating current stimulation (tACS) or oscillatory-tDCS (e.g., at the slow oscillation or at the spindles frequencies) have been successfully applied during sleep for manipulating the targeted brain rhythms and improving both the declarative and the procedural memory consolidation in both humans and rodents (For instance: Campos-Beltrán and Marshall, 2017; Geva-Sagiv et al., 2023; Aksamaz et al., 2024; Nicolas et al., 2025). Moreover, the use of auditory closed-loop stimulation has given positive results on the ongoing sleep rhythms and enhanced the declarative memory in humans (Ngo et al., 2013; Harrington and Cairney, 2021; Moreira et al., 2021; Wunderlin et al., 2021; Schreiner et al., 2024). The manipulation of brain rhythms by exogenous stimulation is an exciting growing field for basic research in humans and animals, with a strong translational potential. Many publications have shown the dependence of the efficacy of the manipulations on the individual spontaneous EEG activity or the network state (Berryhill, 2012; Herrmann et al., 2013; Wiethoff et al., 2014; Chew et al., 2015; Li et al., 2015; Koo and Marshall, 2016; Koo et al., 2018; Chen et al., 2025; Duan et al., 2025; Nicolas et al., 2025). Whereas for basic research a homogenous subject population is used, for findings to have a translational impact, inter-individual differences across many levels must be considered: in particular, differences in the sleep EEG activity with healthy aging, between sexes, and among levels of cognitive ability. The aim of this meta-analysis is to provide a comprehensive reference—essentially for non-invasive brain stimulation (NIBS) research targeting sleep oscillations—regarding the influence of age, sex, and cognitive ability on baseline EEG activity, with a specific emphasis on sleep spindle properties. The latter are strongly associated with neuroplasticity during sleep (Gais et al., 2002; De Gennaro and Ferrara, 2003; Marshall et al., 2006; Mölle et al., 2009, 2011; Dehnavi et al., 2019, 2021; Schreiner et al., 2024; Duan et al., 2025). The relevance of spindle coupling to sleep slow oscillations as recently investigated (Baena et al., 2023) are not covered in this study.

Other meta-analyses have recently been published in relation to normal sleep polysomnography (PSG) across lifespan (Ohayon et al., 2004; Boulos et al., 2019; Weighall and Kellar, 2024), or regarding sleep spindles and cognitive abilities (Reynolds et al., 2018; Ujma, 2018; Chen et al., 2024). However, this meta-analysis investigates both, differences in sleep PSG (plus sleep spindle power), as well as several measures of sleep spindle activity. The present study is divided into three sections, encompassing three meta-analyses. The first and second assess differences in sleep spindles and polysomnography in younger as compared to older subjects (of both sexes), and in females as compared to males (of all ages). The third meta-analysis investigates relationships between sleep spindle properties and cognitive ability in children, adolescents, adults, and healthy elderly, and moderation by sex. For comprehensive reviews on the neurophysiology of sleep spindles we refer to De Gennaro and Ferrara (2003); Fernandez and Luthi (2020); Castelnovo et al. (2025), and Jourde et al. (2025).

## 2 Methods

This meta-analysis was performed in accordance with “PRISMA: Preferred Reporting Items for Systematic Reviews and Meta-Analyses” (Page et al., 2021a,b) and results are presented likewise (Supplementary Table 1). The study protocol was not registered (e.g., in PROSPERO) given its descriptive nature.

### 2.1 Reference search, study selection, and quality assessment

An exhaustive systematic bibliographic search was performed using seven different databases: PubMed (Medline, NCBI), Google Scholar, BioMed Central (BMC, Springer Nature), ScienceDirect (Elsevier), Wiley Online Library (John Wiley & Sons), Livivo (ZB MED) and Cochrane Library. To ensure that all articles reviewed here included sleep spindle measurements, “sleep spindles” was selected as the main keyword for the literature search. Since we were interested in healthy subjects, clinical trial registers were not searched. Some articles were not found during the systematic search but were included given our knowledge of them. The last search was conducted in December 2021 with the addition of one article (Eggert et al., 2021).

All references containing the data of interest and that meet our inclusion criteria were incorporated, even if the topic addressed did not correspond to the main study's theme. Search terms and reasons for exclusion are given in Supplementary Tables 2–7. Inclusion criteria for the meta-analysis were that the study:

1. Was original,
2. Was published in English in an indexed peer-reviewed journal,
3. Used healthy, non-clinical subjects, at least in part (e.g., controls),
4. Showed quantitative data necessary for the meta-analysis (or referring to it),
5. Included sleep spindles and sleep polysomnography, the latter based essentially on EEG measurements:

5.1 Comparing young to older subjects and/or
5.2 Comparing females and males and/or
5.3 Correlating those recordings to measurements of cognitive ability.

All studies that did not meet our inclusion criteria were excluded.

The risk of bias of individual studies (quality) was judged using the Mixed Methods Appraisal Tool (MMAT) with 5 being the maximum score for each study (Pluye and Hong, 2014; Hong et al., 2018, 2019).

The strength of evidence (certainty assessment) for each result was not reported since our outcome does not involve intervention studies (Berkman et al., 2015).

### 2.2 Data analysis

After full-text screening, data were extracted manually from the included studies and added into a personalized data extraction form. The variables selected for our analyses were: mean ± standard deviation (SD), T-statistic, and F-statistic values, correlation coefficient, and sample size.

Some studies referred to but did not report all the data necessary for analyses. Here, authors were contacted by e-mail. The inclusion of this data in the calculations may impact publication bias. If data were already published elsewhere (Ujma, 2018), authors were not contacted. Other studies provided sufficient data to calculate the required information following standard procedures (Rupinski and Dunlap, 1996; Wan et al., 2014; Cumpston et al., 2019): transforming standard error of the mean (SEM) to SD (Landolt et al., 1996; Ehlers and Kupfer, 1997; Mander et al., 2014; Della Monica et al., 2018; Helfrich et al., 2018), using median as an approximate of the mean (Peters et al., 2014; Muehlroth et al., 2019), calculating the approximate duration in minutes of sleep stages from the percentage (Ehlers and Kupfer, 1997; Crowley et al., 2002; Peters et al., 2014; Gaudreault et al., 2018) or using an approximate Pearson correlation coefficient from the Spearman's rank correlation coefficient (Geiger et al., 2011; Hahn et al., 2018; Sulkamo et al., 2019).

The sleep spindle properties meta-analyzed here are density (number/time), amplitude (μV), duration (sec), mean frequency (Hz), spindle count or number, absolute spindle power (μV^2^), relative spindle power (%) and mean density spindle power (μV^2^/Hz). The polysomnographic parameters (PSG) include sleep latency (SLat, min), wake after sleep onset (WASO, min, %), total sleep time (TST, min), sleep efficiency (SEff, percentage of time spent asleep while in bed), sleep stage 1 (S1, min), sleep stage 2 (S2, min), sleep stage 3 (S3, min), sleep stage 4 (S4, min), slow waves sleep (SWS, min), non-rapid eye movement sleep (NREM sleep, min) and rapid eye movement sleep (REM sleep, min). SWS of studies scored by Rechtschaffen and Kales (1968) corresponds to N3 of later American Academy of Sleep Medicine (AASM) scoring criteria (AASM, 2007; Iber et al., 2007). Therefore, N3 stages shown by two studies (Gaudreault et al., 2018; Pesonen et al., 2019) were meta-analyzed in the SWS calculations.

We aimed to select the data as homogeneous as possible given the different recording and analyses methodologies, however some inter-study heterogeneity due to differentially defined frequency bands, spindle detection algorithms, sleep scoring methods, etc. is expected. Interestingly, it has been demonstrated that sleep spindles recorded from different electrode sites have correlations over 0.9 and consequently a few differences in the scalp site from which the data is obtained may not be biasing the meta-analysis (Ujma, 2018). The main characteristics of the studies included in the meta-analyses are shown in Supplementary Tables 9, 17, 25, 27, 29, 31. Note, most of the included studies investigated the spindle frequency range above 11 Hz and detected spindles in 1–3 channels. Thus, slow and fast spindle frequency bands refer to differences within the 11–17 Hz band, and no differentiation is made between potential local and global spindles, both factors of which are suggested to have functional implications (Mölle et al., 2011; Niknazar et al., 2022; Seok et al., 2022). Furthermore, only recent studies have distinguished systematically between spindle properties in S2 (N2) and SWS (N3), mostly revealing for fast spindles topographically wide spread higher density and power in S2, and for frontal slow spindles increased power and density in SWS (Cox et al., 2018; Malerba et al., 2022; Mcconnell et al., 2022; Dehnavi et al., 2023). Thus, NREM sleep depth is reported in the Supplementary Table, but not differentially meta-analyzed. The single study to investigate age-dependent differences of slow spindles within the 9–12.5 Hz frequency range (Muehlroth et al., 2019) was not meta-analyzed with the other studies that delimited slow spindles above 11 Hz (Mander et al., 2014; Fogel et al., 2017). One study correlated sleep spindles in adolescents with cognitive abilities (Bodizs et al., 2014) within a wide spindle frequency range of 9–16 Hz. Given the sleep spindle particularities in that age range (De Gennaro and Ferrara, 2003; Joechner et al., 2023), that study was included in our meta-analysis with the addition of a sensitivity analysis. Different criteria for defining age groups are likely to impact inter-study heterogeneity. Thus, Supplementary Tables 9, 17, 25 report the age-range or the mean age for the groups used by each study. When differences across lifespan were analyzed, the main outcome was the difference between a younger and an older group in sleep spindle properties and, when available, PSG values. Comparisons of sex, correspond to differences between female and male groups of healthy subjects (cp. 2.3. Statistics for directionality). Three studies showed data from several age groups from which we each selected a young and old group that met our criteria (25–34 and 67–79 years old from Principe and Smith, 1982; 20–29 and 60–69 years old from Nicolas et al., 2001; 20–30 and 65–84 years old from Della Monica et al., 2018). Data from some studies were combined or separated to fit our categories, as long as studies fulfilled inclusion criteria (Ehlers and Kupfer, 1997; Crowley et al., 2002; Fogel et al., 2007; Peters et al., 2008; Della Monica et al., 2018). Data of the longitudinal study by Hahn et al. (2018) stem from the same subjects (*n* = 32), as children and later as adolescents. We included both (dependent) groups in our analyses.

To analyze the effect of cognitive ability, we focused on properties of sleep spindles because it was the only measurement in the published literature that met our criteria. When studies conducted several tests of cognitive ability, we selected the most universal test reflecting general ability, e.g., fluid intelligence.

Of the cognitive ability tests, The Wechsler scales of intelligence were most frequently used, either the adult (WAIS) or the children's version (WISC), that measure fluid and crystalline intelligence (Abdelhamid et al., 2021). In addition, two studies used Raven's Progressive Matrices, either the standard form (Raven Progressive Matrices Test, RPMT) or the advanced form (APM), that provides an estimate measure of non-verbal fluid intelligence (Bilker et al., 2012). One study used the Stanford-Binet Intelligence Scale, closely similar to the Wechsler scale, but not differentiating between fluid and crystalline intelligence. Further tests in individual studies were the Cambridge Brain Sciences Trials, the MAB: Multidimensional Aptitude Battery, the Zahlen-Verbindungs-Test, and the CFIT: Culture Fair Intelligence Test. For the exact references of each task and methodology, please refer to the original studies.

### 2.3 Statistics

Some studies consisted only of small samples for which Hedges' g value provides the best effect size estimate (Grissom and Kim, 2005). Therefore, the Hedges' g value was used to estimate all differences between age and sex groups. Hedges' g value is calculated by dividing the differences between the groups' means by the pooled and weighted standard deviation (Hedges, 1981; Durlak, 2009). Interpretation follows “the rule of thumb”, namely absolute values of effect sizes (Hedge's g) around 0.20 are small, around 0.50 are medium and around or above 0.80 are large (Hedges, 1981; Durlak, 2009).

In this review, positive effect sizes indicate that old (or male) subjects revealed larger mean values than young (or female) subjects. Effect sizes for the relation between cognitive ability and sleep spindles were calculated as averaged correlation coefficients, using the Fisher's r-to-Z-transformation method (Fisher, 1921) and resultant numbers provide the direction (positive or negative) and the strength (magnitude) of that relationship. Effect sizes are shown in forest plots, with subcategories listed alphabetically. All results are discussed using the random effects model (Dersimonian and Laird, 1986), but the Supplementary Table also provide the fixed effects model, and the corresponding levels of significance (95% confidence interval). When only one study was available for a category (e.g., for slow sleep spindle frequency), the effect size of the study was calculated using the fixed-effects model and data were only reported in a Supplementary Table.

Heterogeneity among studies was assessed using the Cochran's Q, I^2^, Tau, and the T^2^ statistics (Dersimonian and Laird, 1986). The Cochran's Q test calculates differences among the effect sizes (of the individual studies) and the combined effect size, both weighted and averaged, following a *X*^2^ distribution (Cochran, 1950). The significance of the Q-statistic defines an undetermined degree of heterogeneity (Hak et al., 2016). Statistic I^2^ shows the percentage of observed variance that is likely to remain if we could remove the sampling error (Higgins and Thompson, 2002; Higgins et al., 2003; Abdelhamid et al., 2021). A low I^2^ value (e.g., ≤ 25%), indicates that dispersion in the corresponding forest plot would disappear if the sampling error could be removed. Tau and T^2^ estimate the between-study variance of true effect sizes, independent of the number of studies. For large I^2^ (e.g. ≥ 75%; often in the case of small study numbers), tau and T^2^ should be compared since they estimate the distribution of true effect sizes across studies. These values are given in Supplementary Table (i.e., Dersimonian and Laird, 1986; Higgins and Thompson, 2002; Higgins et al., 2003; Hak et al., 2016). The prediction intervals (95%) describe the range of the effects sizes and are calculated considering Tau and available in the forest plots (Hak et al., 2016). We focused on I^2^ and T^2^ statistics for interpreting results since these are more suitable for studies with small subject numbers (Higgins and Thompson, 2002; Higgins et al., 2003).

The risk of publication bias was controlled using both the Egger's regression and the Begg and Mazumbar's correlation (Begg and Mazumdar, 1994; Egger et al., 1997) and shown with funnel plots. Egger's regression was performed for more than two publications, as also mentioned in the discussion. Publication bias, meaning that small studies and/or negative results are less likely to be published, is considered when the test output is significant (Borenstein, 2009). In case of asymmetrical funnel plots, the Trim-and-Fill method was used. However, interpretation of publication bias of highly heterogeneous studies could be biased (Hak et al., 2016). Publication bias was not applicable for the studies analyzing the differences between sexes in sleep spindle and PSG parameters.

Subgroup analyses were performed when sufficient data was available to elaborate subsets (e.g., age groups). Sensitivity analyses were performed to compare results when eliminating a study (e.g., studies with lower MMAT scores; Supplementary Table 8).

Meta-regression analyses were performed to control the potential linear effect of moderators (e.g., percentage of females) on the dependent variable, and results are shown in Supplementary Tables 16, 24, 40 and Supplementary Figures 4, 5, 7 (bubble plots). The proportion of the heterogeneity in our random-effects models attributable to each moderator was calculated by the coefficient of determination (R^2^). All statistical analyses were performed using Meta-Essentials version 1.4 (Van Rhee et al., 2015; Hak et al., 2016; Suurmond et al., 2017).

## 3 Results

A total of 2275 citations were obtained from the systematic literature search (August 2020), from which a total of 41 studies (k = 41) with a total sample size of 1788 subjects reached our inclusion criteria and were included in the meta-analysis after eliminating duplicates, title and abstract screening and the final full-text screening (Supplementary Tables 2–7). An updated systematic literature search following the same search parameters as stated above (data not shown) was performed in December 2021 resulting in the addition of one study (k = 42) with a total sample size of 1878 healthy subjects. Introduction and discussion are supplemented by results from studies up to July 2025.

The quality of the studies (risk of bias) was assessed using the MMAT (Pluye and Hong, 2014; Hong et al., 2018, 2019). The main topic from most of the studies was different from the focus of this meta-analysis and despite having higher scores in the MMAT (Supplementary Table 8), not all studies provided all necessary data. Only two studies had a score below 3 (Gaillard and Blois, 1981; Principe and Smith, 1982). For controlling the impact of the studies with lower scores, sensitivity analyses were performed.

The next sections provide the results of the three meta-analyses: (3.1) Sleep spindle properties and PSG-age-dependent differences; (3.2) Sleep spindle properties and PSG sex-dependent differences; and (3.3) Sleep spindle properties and cognitive ability. Section is subdivided into subsections with results for sleep spindle properties and when available, sleep macrostructure.

### 3.1 Sleep spindle properties and polysomnography—age-dependent differences

**Figure 1:**
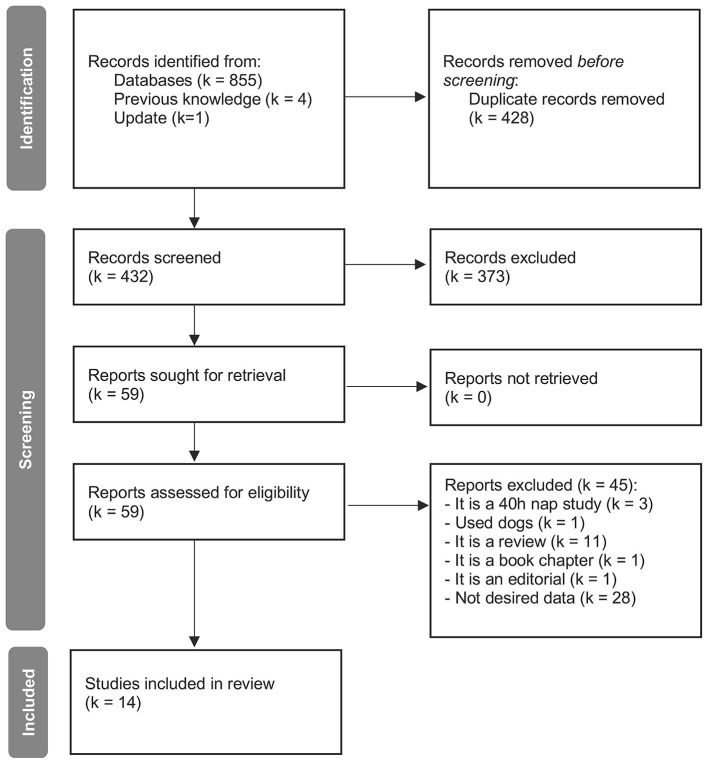
PRISMA 2020 flow diagram for the reference search on differences in sleep electrophysiology and sleep spindles dependent upon age. k = number of studies.

For meta-analyzing the effects of age a total of 14 studies with a total sample size of 626 subjects (336 aged, 47% females) were included (Figure 1 and Supplementary Tables 9–11; Principe and Smith, 1982; Guazzelli et al., 1986; Landolt et al., 1996; Nicolas et al., 2001; Crowley et al., 2002; Peters et al., 2008; Mander et al., 2014; Peters et al., 2014; Fogel et al., 2017; Della Monica et al., 2018; Gaudreault et al., 2018; Helfrich et al., 2018; Muehlroth et al., 2019; Eggert et al., 2021). The studies included met our inclusion criteria as listed in the Methods section 2.1 for comparing at least two age groups of interest.

#### 3.1.1 Sleep spindle properties

**Figure 2:**
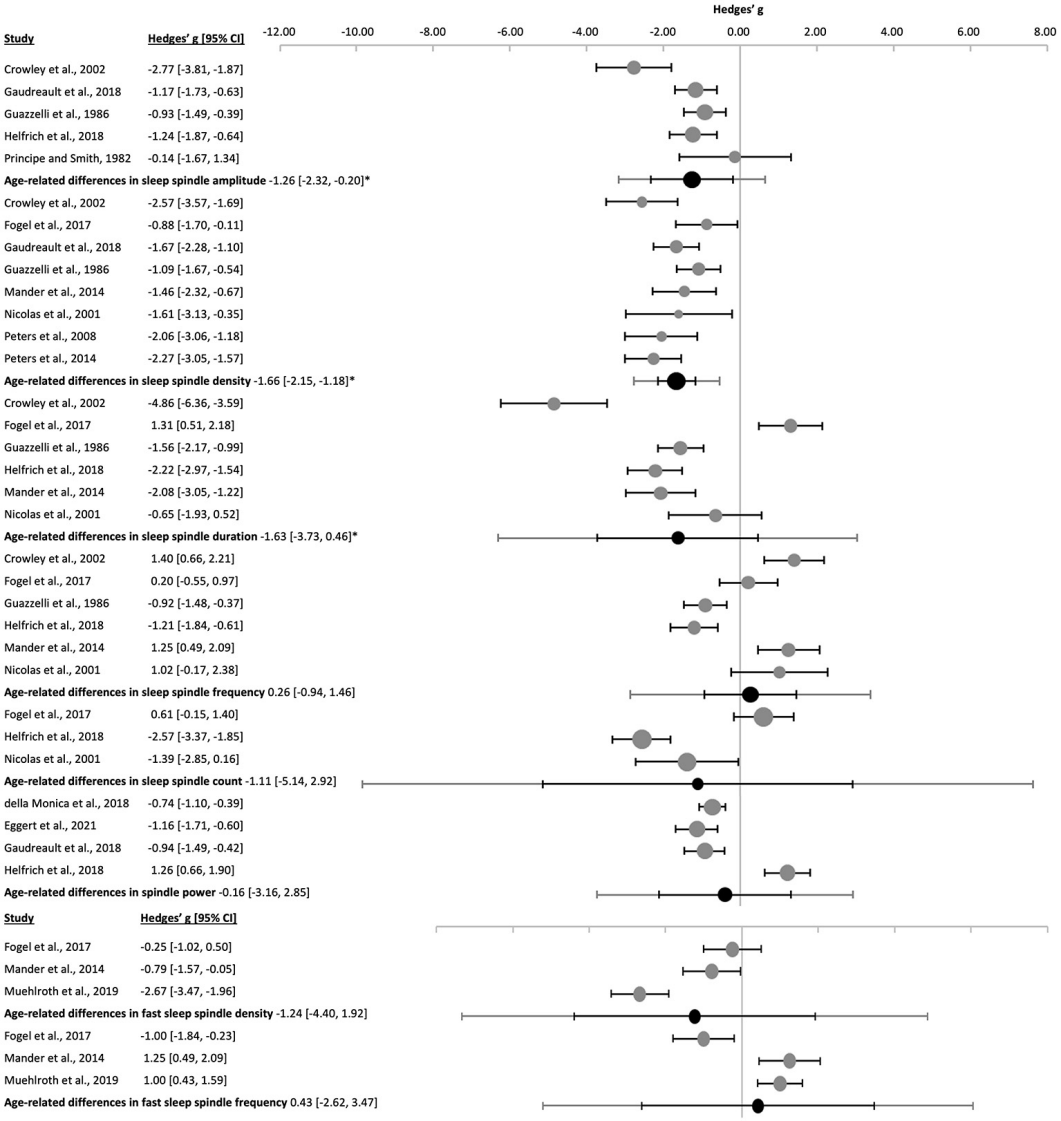
Forest plot of age-related differences in sleep spindle properties. For each individual study effect sizes (gray dots) and the 95% confidence intervals (black lines) are shown regarding all **(top)** and fast **(bottom)** sleep spindle. The combined effect size (black dot), the combined confidence intervals (black line) and the prediction interval (gray line) using the random-effects model follow. The size of the dots represents the weight of the study (gray) or the combined weight (black). Negative Hedges' g values indicate that the younger group revealed a higher mean value of the corresponding parameter. *p_g_ ≤ 0.05.

Sleep spindles of older subjects had a lower amplitude [k = 5, *n* = 221, g = −1.26 (−2.32, −0.20), p_g_ = 0.001, *I*^2^ = 72.87%], decreased density [k = 8, *n* = 307, g = −1.66 (−2.15, −1.18), p_g_ = 4 x 10^−16^, *I*^2^ = 55.37%], and shorter duration [k = 6, *n* = 222, g = −1.63 (−3.73, 0.46), p_g_ = 0.045, *I*^2^ = 93.74%] than younger adults (Figure 2 and Supplementary Table 12). However, neither spindle frequency [k = 6, *n* = 222, g = 0.26 (−0.94, 1.46), p_g_ = 0.580, *I*^2^ = 90.71%], sleep spindle count [k = 3, *n* = 92, g = −1.11 (−5.14, 2.92), p_g_ = 0.235, *I*^2^ = 94.43%], nor spindle power [k = 4, *n* = 303, g = −0.41 (−2.16, 1.35), p_g_ = 0.461, *I*^2^ = 93.01%] were significantly reduced in elderly (Figure 2 and Supplementary Table 12). No risk of publication bias was observed and despite a few outliers, the resulting funnel plots were symmetrical (Supplementary Table 12 and Supplementary Figure 1).

Given the possible impact of the (non-digital) methodology widely used up to the 1980s-decade and the lower MMAT scores, sensitivity analyses were performed eliminating these older studies (Principe and Smith, 1982; Guazzelli et al., 1986). Deleting the data from Guazzelli et al. (1986) resulted in similar results in sleep spindle density, amplitude and frequency, but eliminated the significant difference in sleep spindle duration between young and older subjects (Supplementary Table 14). Removing Principe and Smith (1982) augmented the risk of publication bias (Egger's regression's *p* = 0.010), but the age-related differences in sleep spindle amplitude remained similar (Supplementary Table 14).

One of the studies analyzed activity during a nap, possibly introducing a bias given the different sleep duration and composition. Subsequent, sensitivity analyses eliminating Fogel et al. (2017), produced, however, similar results (Supplementary Table 15).

Meta-regression analyses did not show any significant relationships between the percentage of females and the age-related differences in sleep spindle properties (Supplementary Table 16). Yet, bubble plots (Supplementary Figure 4) indicated, albeit not significant, that studies with a relatively greater number of females found higher spindle power.

#### 3.1.2 Fast sleep spindle properties

Only a few studies distinguished between fast and slow spindles, thus limiting strength of findings on the differences in fast sleep spindles with healthy aging (Figure 2 and Supplementary Table 12). The most robust finding was a trend toward reduced fast sleep spindle density [k = 3, *n* = 111, g = −1.24 (−4.40, 1.92), p_g_ = 0.092, *I*^2^ = 91.38%] in older as compared to younger subjects. Changes in spindle duration [k = 2, *n* = 58, g = −0.30 (−18.54, 17.95), p_g_ = 0.837, *I*^2^ = 95.93%], mean frequency [k = 3, *n* = 111, g = 0.43 (−2.62, 3.47), p_g_ = 0.547, *I*^2^ = 90.62%], and spindle count [k = 2, *n* = 81, g = −0.70 (−18.18, 16.78), p_g_ = 0.611, *I*^2^ = 96.59%] of fast sleep spindles were not significantly altered in aged subjects. Fast sleep spindle amplitude was only analyzed in one of the included studies (*n* = 53, g = −0.45, p_g_ = 0.100; Muehlroth et al., 2019). Landolt et al. (1996) was the only study to analyze relative spindle power between 13 and 15 Hz and found significantly increased power in old as compared to young subjects in the 13–14 Hz frequency range (*n* = 16, g = 1.45, p_g_ = 0.007), but not in the 14–15 Hz frequency range (*n* = 16, g = 0.37, p_g_ = 0.443).

The studies were highly heterogeneous, yet with no publication bias (Supplementary Table 12 and Supplementary Figure 2). Removal of the nap study (Fogel et al., 2017) did not affect fast spindle density or any other result (Supplementary Table 15). Both density and mean frequency of fast sleep spindles were significantly associated with the percentage of females from the limited samples analyzed here (Supplementary Figure 4 and Supplementary Table 16).

#### 3.1.3 Slow sleep spindle properties

Age-dependent slow sleep spindle properties were analyzed in even fewer studies than for fast spindles (Supplementary Table 12). Spindle density was significantly reduced in elderly as compared to young subjects [k = 2, *n* = 58, g = −1.45 (−3.80, 0.91) p_g_ = 0.000, *I*^2^ = 0.00%]. Slow spindle duration [k = 2, *n* = 58, g = 0.03 (−23.89, 23.94), p_g_ = 0.989, *I*^2^ = 97.29%], and frequency [k = 2, *n* = 58, g = 1.19 (−14.17, 16.54), p_g_ = 0.163, *I*^2^ = 93.73%] did not differ significantly with age. Interestingly, for older subjects, slow spindle count (k = 1, *n* = 28, g = 0.73, p_g_ = 0.027) and relative power in the 12–13 Hz range (*n* = 16, g = 1.64, p_g_ = 0.003) were significantly increased in the both of the studies that investigated these age-related differences (Landolt et al., 1996; Fogel et al., 2017). Sensitivity analyses removing the nap study (Fogel et al., 2017) was not performed given the paucity of studies.

#### 3.1.4 Polysomnography

**Figure 3:**
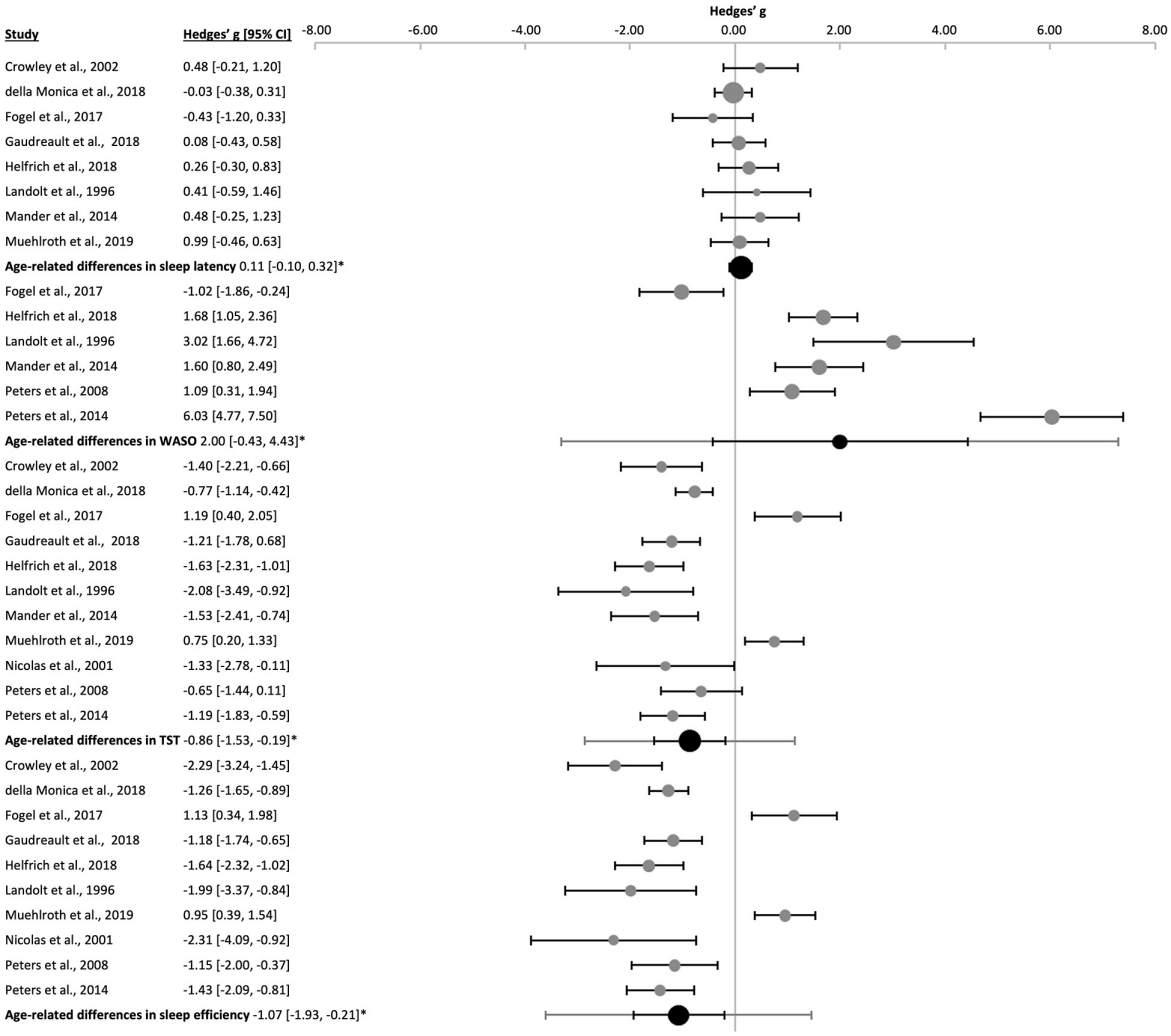
Forest plot of the age-related differences in several polysomnography parameters. Individual effect sizes (gray dots) and the 95% confidence intervals (black lines) are shown for each study, followed by the combined effect size (black dot), the combined confidence intervals (black line) and the prediction interval (gray line) using the random-effects model. The size of the dots represents the weight. Negative Hedges' g values indicate that the younger group revealed a higher mean value of the corresponding parameter. *p_g_ ≤ 0.05.

Meta-analyses of the sleep macrostructure implicated in sleep quality denoted poorer sleep in aged subjects (Figure 3 and Supplementary Table 13) characterized by more wake after the sleep onset [WASO, k = 6, *n* = 202, g = 2.00 (−0.43, 4.43), p_g_ = 0.035, *I*^2^ = 94.53%], shorter total sleep time [TST, k = 11, *n* = 492, g = −0.86 (−1.53, −0.19), p_g_ = 0.004, *I*^2^ = 86.96%] and lower sleep efficiency [k = 10, *n* = 462, g = −1.07 (−1.93, −0.21), p_g_ = 0.005, *I*^2^ = 90.78%]. Sleep latency was not significantly affected by age [k = 8, *n* = 404, g = 0.11 (−0.10, 0.32), p_g_ = 0.230, *I*^2^ = 0.00%]. No publication bias was observed (Supplementary Table 13 and Supplementary Figure 3). Removing the nap study (Fogel et al., 2017) resulted in similar results but augmented the risk of publication bias in the changes in sleep latency with age (Egger's regression's *p* = 0.006, Begg and Mazumbar's *p* = 0.024, Supplementary Table 15).

**Figure 4:**
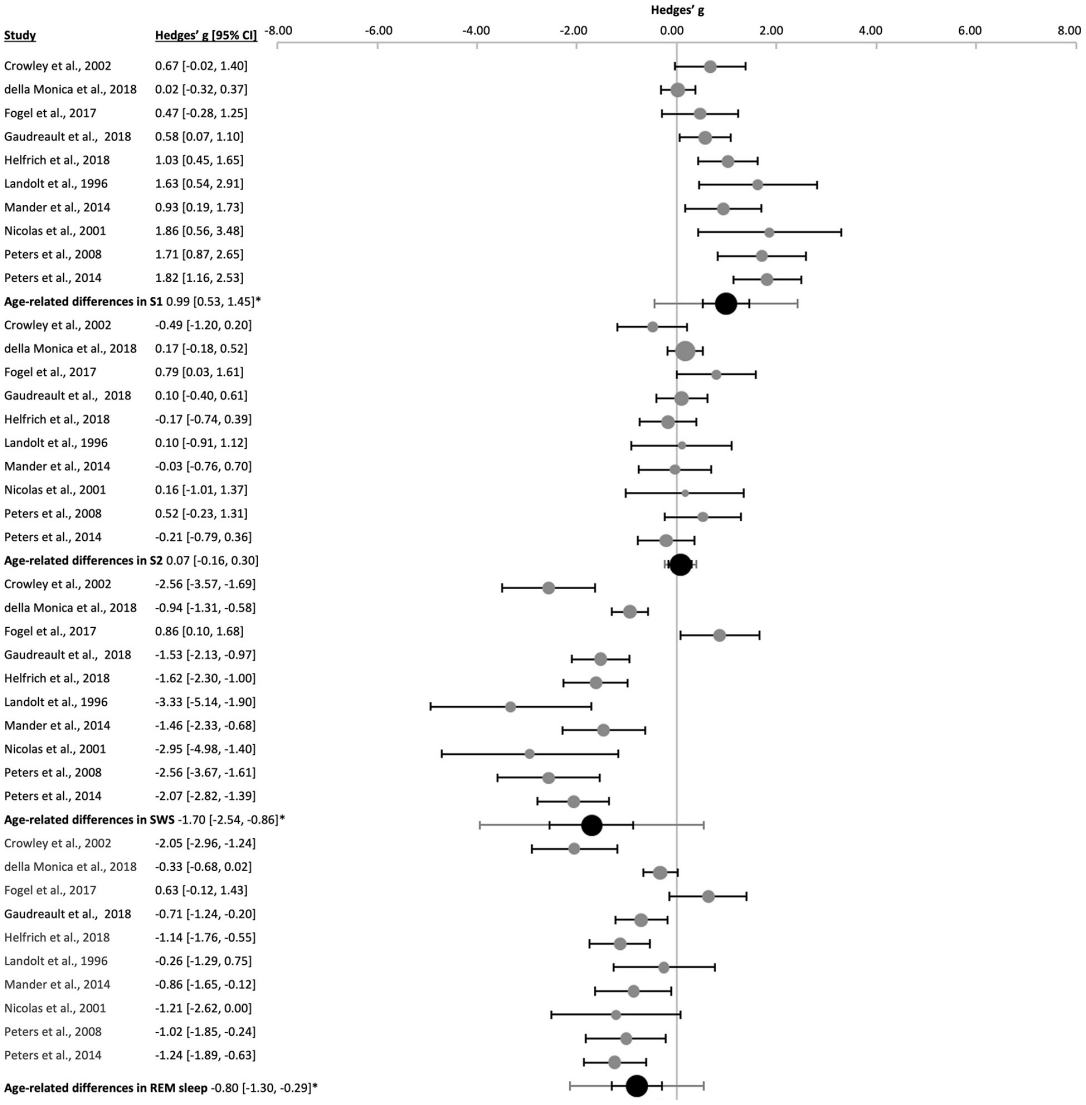
Forest plot of the age-related differences in sleep stages. Individual effect sizes (gray dots) and the 95% confidence intervals (black lines) are shown for each study, followed by the combined effect size (black dot), the combined confidence intervals (black line) and the prediction interval (gray line) using the random-effects model. The size of the dots represents the weight. Negative Hedges' g values indicate that the younger group revealed a higher mean value of the corresponding parameter. *pg ≤ 0.05.

When needed, the reported percentage of the sleep stages were converted into minutes as an approximation (Nicolas et al., 2001; Crowley et al., 2002; Peters et al., 2014; Gaudreault et al., 2018) to analyze the sample together. Concordant with poorer sleep quality, durations of manifested deep NREM sleep and REM sleep were shorter in the older subjects (Figure 4 and Supplementary Table 13): duration of S3 [k = 2, *n* = 28, g = −1.53 (−4.38, 1.32), p_g_ = 8 x 10^−12^, *I*^2^ = 0.00%], S4 [k = 3, *n* = 158, g = −2.39 (−5.18, 0.40), p_g_ = 0.0002, *I*^2^ = 80.26%], SWS [k = 10, *n* = 439, g = −1.70 (−2.54, −0.86), p_g_ = 4 x 10^−6^, *I*^2^ = 86.54%] and REM sleep [k = 10, *n* = 439, g = −0.80 (−1.30, −0.29), p_g_ = 0.0003, *I*^2^ = 74.14%]. Whereas S1 duration [k = 10, *n* = 439, g = 0.99 (0.53, 1.45), p_g_ = 1 x 10^−6^, *I*^2^ = 77.19%] was longer in aged subjects, S2 duration [k = 10, *n* = 439, g = 0.07 (−0.16, 0.30), p_g_ = 0.485, *I*^2^ = 8.65] was not altered significantly.

Apart from the significant Egger's regression found for S1 (*p* = 0.023), no other risks of publication bias were observed (Supplementary Table 13 and Supplementary Figure 3). Removing the nap study (Fogel et al., 2017) when performing sensitivity analyses did not change the overall results, but the risk of publication bias appeared for SWS (Egger's regression's *p* = 0.002, Begg and Mazumbar's *p* = 0.022, Supplementary Table 15).

### 3.2 Sleep spindle properties and sleep polysomnography—sex dependent differences

**Figure 5:**
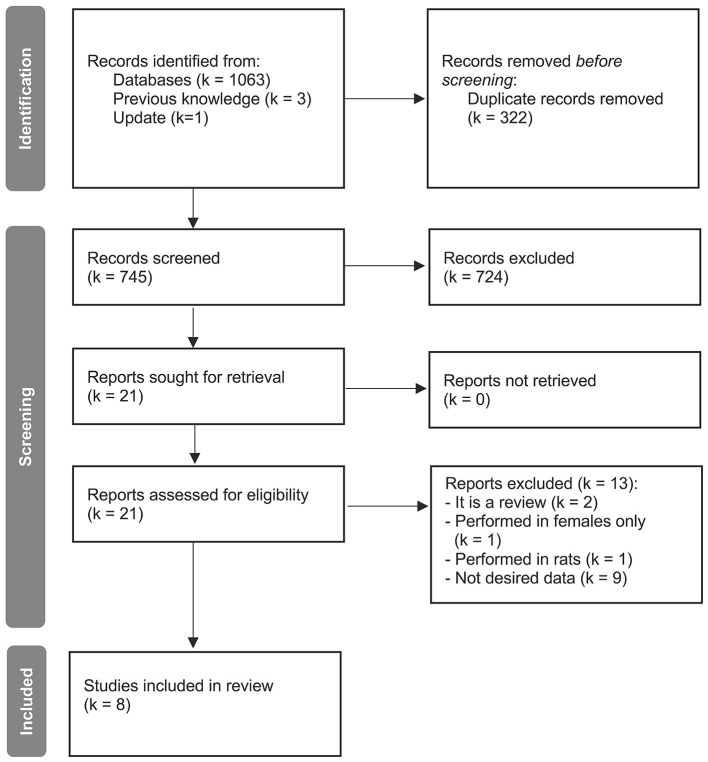
PRISMA 2020 flow diagram for the reference search on the differences in sleep electrophysiology and sleep spindles between sexes. *k* = number of studies.

For meta-analyzing the differences between sexes in the sleep EEG, a total of eight studies divided into twelve sub-studies with a total sample size of 747 subjects (384 females, 51.4% females) were included (Figure 5 and Supplementary Tables 17–19; Gaillard and Blois, 1981; Ehlers and Kupfer, 1997; Crowley et al., 2002; Huupponen et al., 2002; Ujma et al., 2014; Della Monica et al., 2018; Pesonen et al., 2019; Eggert et al., 2021). The studies included met our inclusion criteria as listed in the Methods section 2.1 for comparing females vs. males and some of them allowed us to compare sexes divided by age sub-groups. Data from Crowley et al. (2002) was divided into two sub-studies by age, with a young and an old group. The study of Della Monica et al. (2018), was divided into three sub-studies by age: a young, middle-aged and old group. Data of Ehlers and Kupfer (1997) provided two sub-studies for age: a young and middle-aged group. Data from Pesonen et al. (2019) were meta-analyzed with the young groups since their subjects were all 17 years. The rest of the studies were treated as young groups (Gaillard and Blois, 1981), old groups (Eggert et al., 2021) or all age-ranges (Huupponen et al., 2002; Ujma et al., 2014).

#### 3.2.1 Sleep spindle properties

**Figure 6:**
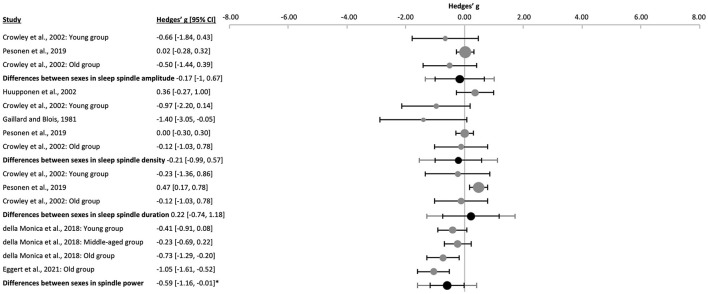
Forest plot of the differences between sexes in sleep spindle properties. The individual effect size (gray dots) and the 95% confidence intervals (black line) are shown for each study, followed by the combined effect size (black dot), the combined confidence intervals (black line) and the prediction interval (gray line) using the random-effects model. The size of the dots represents the weight. Negative Hedges' g values indicate that females revealed a higher mean value of the corresponding parameter than males. *p_g_ ≤ 0.05.

There were no significant differences between females and males in sleep spindle amplitude [k = 3, *n* = 210, g = −0.17 (−1.00, 0.67), p_g_ = 0.391], density [k = 5, *n* = 260, g = −0.21 (−0.99, 0.57), p_g_ = 0.452, *I*^2^ = 55.70%, Egger's regression's *p* = 0.078, Begg and Mazumbar's rank correlation/test *p* = 0.05; Figure 6 and Supplementary Table 20], nor in duration [k = 3, *n* = 210, g = 0.22 (−0.74, 1.18), p_g_ = 0.331]. Absolute spindle band power was significantly lower in males than females [*n* = 206, g = −0.59 (−1.16, −0.01), p_g_ = 0.001]. Mean spindle frequency was also significantly lower in males [k = 2, *n* = 34, g = −0.34 (−1.56, 0.87), p_g_ = 0.0003]. However, there was no effect of sex on mean spindle power density (11–16 Hz), i.e., relative to background activity, meta-analyzed from the two available samples of Ehlers and Kupfer (1997) [*n* = 61, g = −0.35 (−3.72, 3.01), p_g_ = 0.180].

To control for possible bias by differences in methodology, sensitivity analyses were performed by removing the oldest publication (Gaillard and Blois, 1981) from the sleep spindle density meta-analysis. The comparison remained non-significant [k = 4, *n* = 250, g = −0.04 (−0.71, 0.64), p_g_ = 0.864, Supplementary Table 21].

When analyzing the samples separately by age group, differences in sleep spindle properties revealed only minor discrepancies compared to the whole data set (Supplementary Table 23). In addition, no significant effects of age were found by meta-regression in any sleep spindle parameter (Supplementary Figure 5 and Supplementary Table 24).

#### 3.2.2 Fast sleep spindle properties

Only one study analyzed the differences in fast sleep spindle properties between females and males (Ujma et al., 2014). In this study, males had higher fast sleep spindle density (g = 0.32, p_g_ = 0.047) and duration (g = 0.41, p_g_ = 0.011), and lower amplitude (g = −0.56, p_g_ = 0.0005) and frequency (g = −0.65, p_g_ = 0.00006) than females in a sample of 160 subjects with an age range from 17 to 69 years (Supplementary Table 20).

#### 3.2.3 Slow sleep spindle properties

Similarly, only one study compared the effect of sex on slow sleep spindle properties: Ujma and colleagues (Ujma et al., 2014) found a significantly lower slow spindle frequency in males than females from 17 to 69 years (*n* = 160, g = −0.45, p_g_ = 0.005, Supplementary Table 20).

#### 3.2.4 Polysomnography

**Figure 7:**
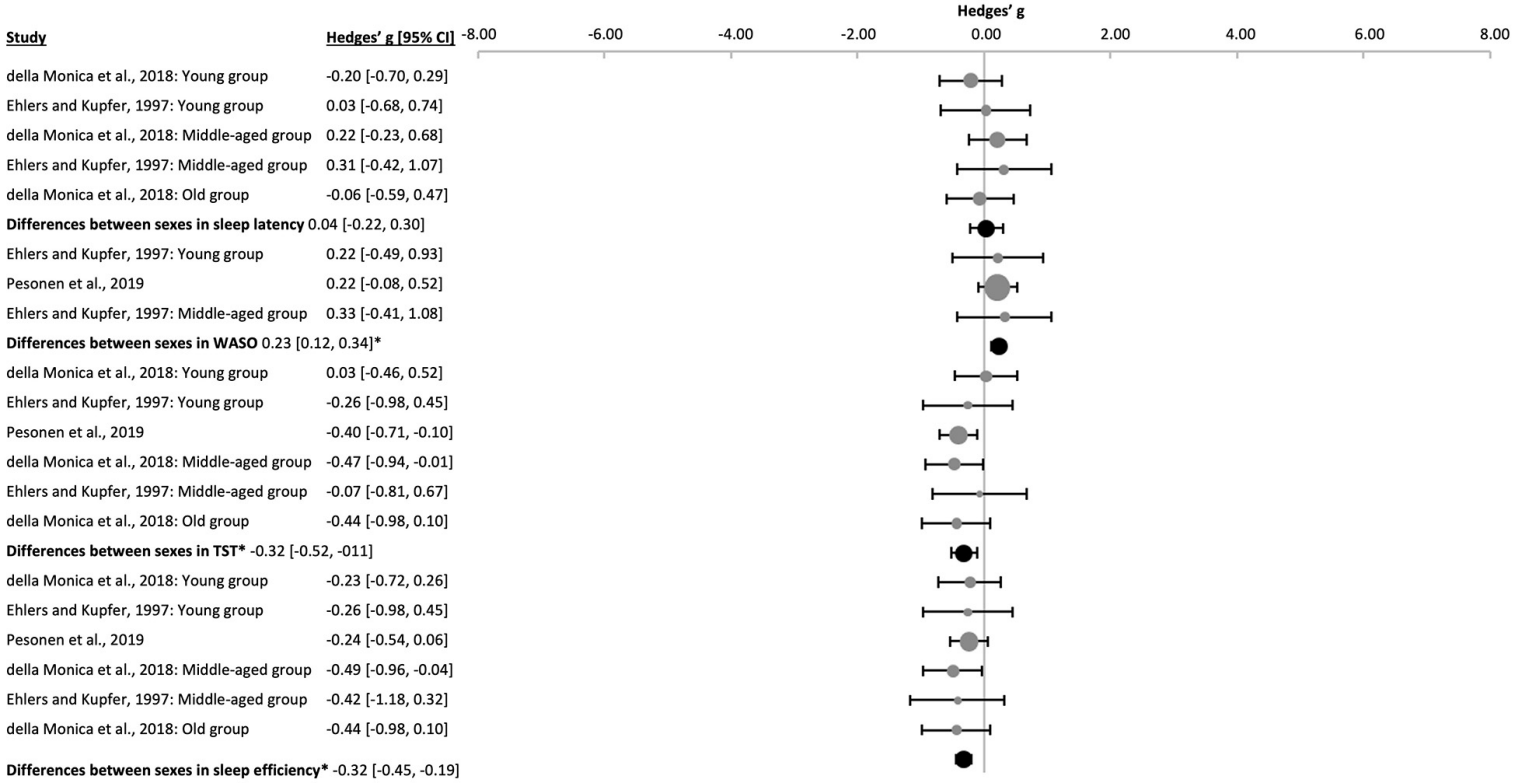
Forest plot of the differences between sexes in several sleep properties. The individual effect sizes (gray dots) and the 95% confidence intervals (black lines) are shown for each study, followed by the combined effect size (black dot), the combined confidence intervals (black line) and the prediction interval (gray line) using the random-effects model. The size of the dots represents the weight. Negative Hedges' g values indicate that females revealed a higher mean value of the corresponding parameter than males. *p_g_ ≤ 0.05.

Overall, sleep was more consolidated in females than males, however not all parameters reached significance (Figure 7 and Supplementary Table 22). Males revealed more awakenings during the night [WASO, k = 3, *n* = 237, g = 0.23 (0.12, 0.34), p_g_ = 0.000], had shorter TST [k = 6, *n* = 443, g = −0.32 (−0.52, −0.11), p_g_ = 8 x 10^−5^] and worse sleep efficiency [k = 6, *n* = 443, g = −0.32 (−0.45, −0.19), p_g_ = 6 x 10^−11^]. Sleep latency did not differ significantly [k = 5, *n* = 267, g = 0.04 (−0.22, 0.30), p_g_ = 0.678]. However, when meta-analyzing young, middle-aged, and healthy aged subjects separately, similar effect sizes for WASO, TST and sleep efficiency were seen (Figure 7 and Supplementary Table 23). Sleep latency was neither significantly shorter in young males [k = 2, *n* = 98, g = −0.13 (−1.51, 1.26), p_g_ = 0.247] nor in the one study that analyzed older subjects (k = 1, *n* = 64, g = −0.06, p_g_ = 0.819, Supplementary Table 23).

**Figure 8:**
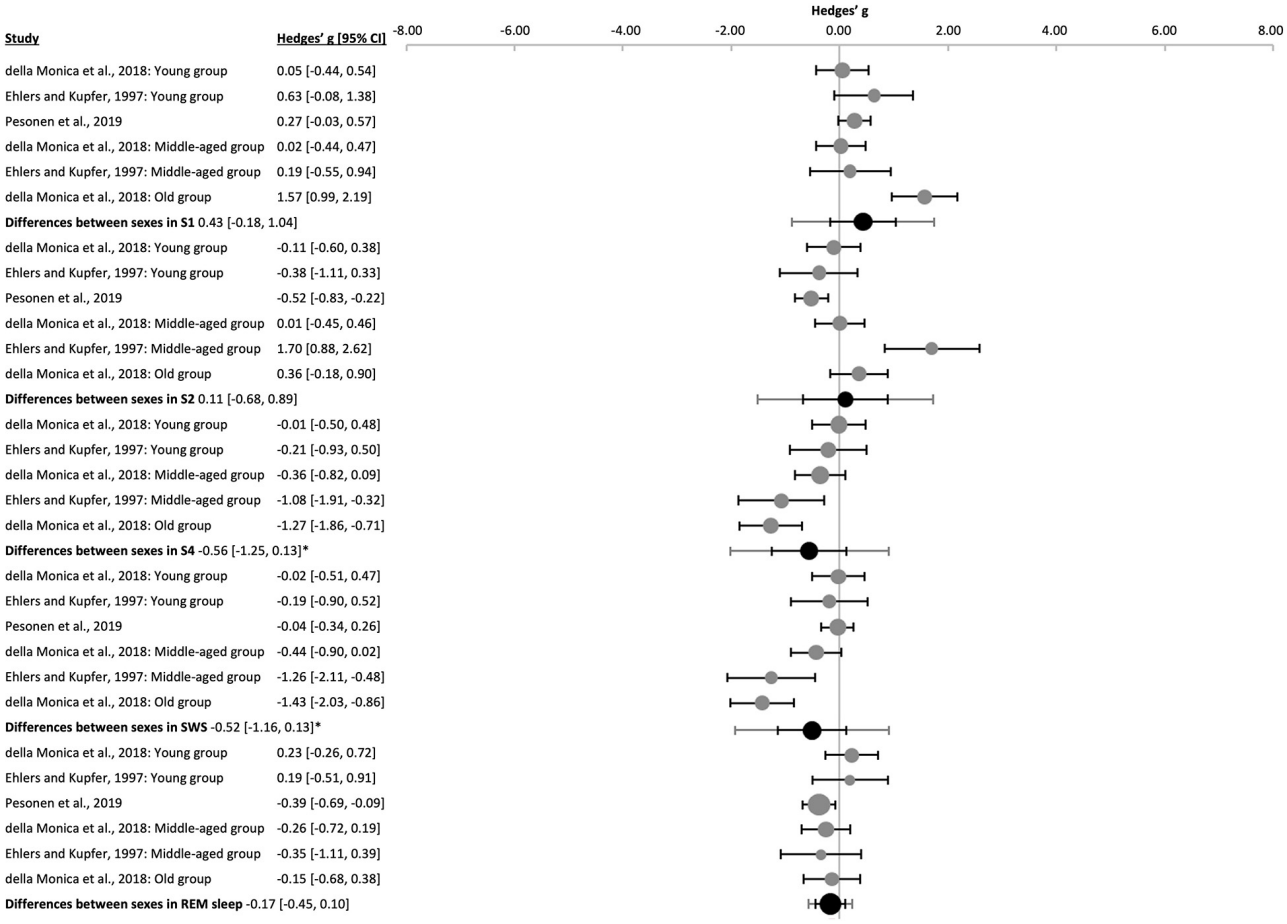
Forest plot of the differences between sexes in sleep stages. The individual effect size (gray dots) and the 95% confidence intervals (black line) are shown for each study, followed by the combined effect size (black dot), the combined confidence intervals (black line) and the prediction interval (gray line) using the random-effects model. The size of the dots represents the weight. Negative Hedges' g values indicate that females revealed a higher mean value of the corresponding parameter than males. *Pg ≤ 0.05.

Sleep stages were reported both as percentage of TST (Ehlers and Kupfer, 1997) or as duration in minutes (Della Monica et al., 2018; Pesonen et al., 2019). For this meta-analysis, data on the percentage of the sleep stages were converted into minutes. The duration of S4 [k = 5, *n* = 276, g = −0.56 (−1.25, 0.13), p_g_ = 0.023] and SWS [k = 6, *n* = 443, g = −0.52 (−1.16, 0.13), p_g_ = 0.039] were significantly shorter in males as compared to females (Figure 8 and Supplementary Table 22). The duration of REM sleep was shorter [k = 6, *n* = 443, g = −0.17 (−0.45, 0.10), p_g_ = 0.106], yet failing to reach significance, and S1 tended to be longer [k = 6, *n* = 443, g = 0.43 (−0.18, 1.04), p_g_ = 0.068] in males. Interestingly, the difference between sexes in the duration of S2 was not significant when analyzing all the ages together [k = 6, *n* = 443, g = 0.11 (−0.68, 0.89), p_g_ = 0.724], in the middle-age group [k = 2, *n* = 105, g = 0.82 (−9.96, 11.60), p_g_ = 0.336] or in the older sample alone (k = 1, *n* = 64, g = 0.36, p_g_ = 0.184), but S2 duration was significantly shorter in young males [k = 3, *n* = 274, g = −0.40 (−0.93, 0.13), p_g_ = 0.001, Supplementary Table 23]. Differences in significance appeared for S4 and SWS, as well as trends for S1 and REM sleep appeared when dividing the sample by age groups (Supplementary Table 23).

### 3.3 Sleep spindle properties and cognitive ability

**Figure 9:**
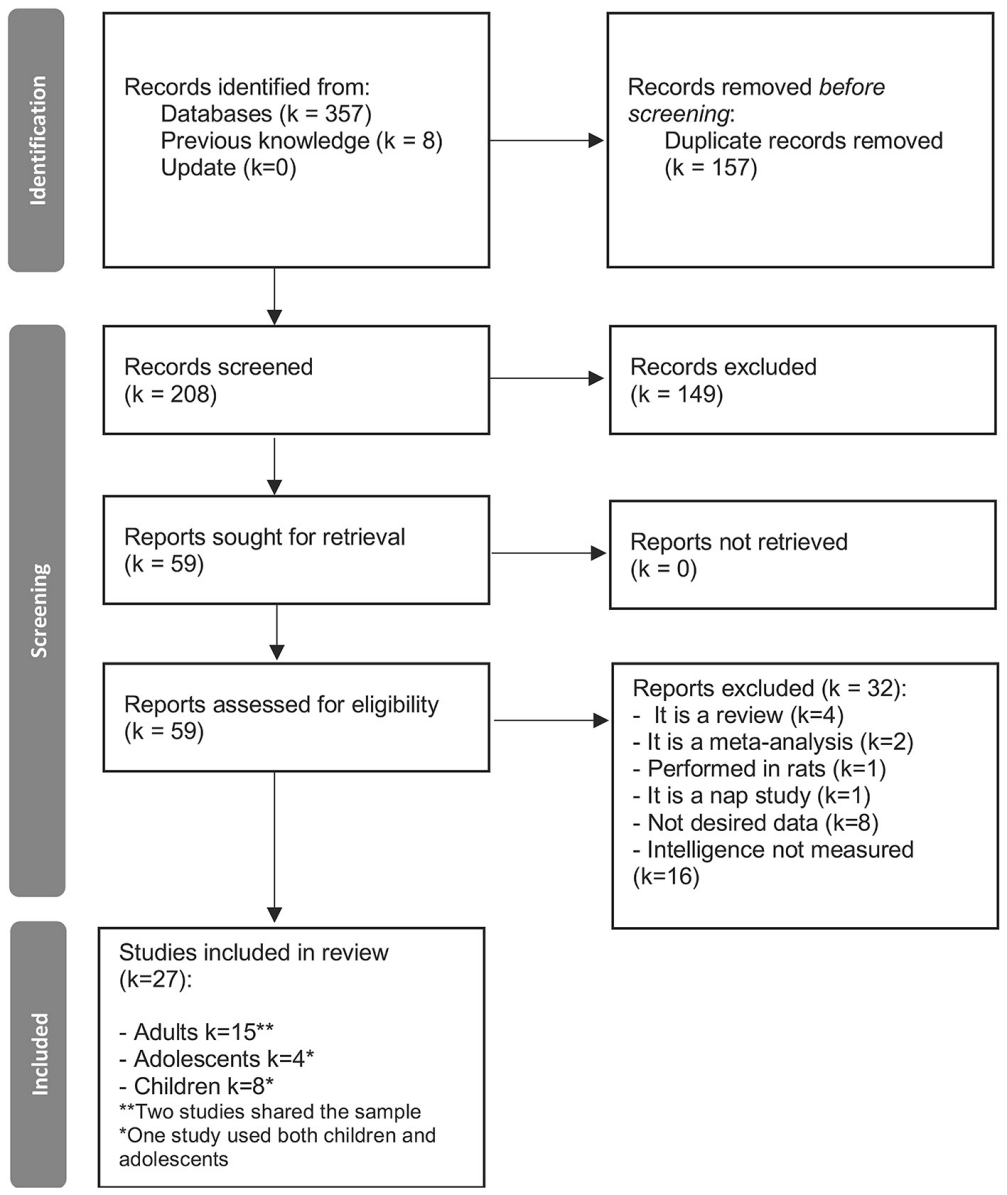
PRISMA 2020 flow diagram for the reference search on the differences in sleep spindles in relation to cognitive ability. *k* = number of studies.

For meta-analyzing the relationship between sleep spindles and cognitive ability, a total of 27 studies with a total sample of 1063 subjects (526 females, 49.5% females) were included (Figure 9). The studies met our inclusion criteria as listed in the Methods section 2.1. These studies were meta-analyzed together as well as separating according to age group: Children, adolescents, adults, and healthy aged subjects. A total of 8 studies with a total sample size of 216 (108 females, 50% females) were included in the children's group (Supplementary Tables 25, 26; Geiger et al., 2011; Chatburn et al., 2013; Gruber et al., 2013; Hoedlmoser et al., 2014; Tessier et al., 2015; Ujma et al., 2016; Hahn et al., 2018; Sulkamo et al., 2019). A total of 4 studies with a total sample of 266 (153 females, 57.5% females) were included in the adolescent group (Supplementary Tables 27, 28; Bodizs et al., 2014; Nader and Smith, 2015; Hahn et al., 2018; Pesonen et al., 2019). Hahn et al. (2018) was a longitudinal study in which the same sample was analyzed during childhood and as adolescents.

A total of 15 studies divided into 18 sub-studies with a total sample size of 615 (289 females, 47% females) were included in the adult group (Supplementary Tables 29, 30; Bodizs et al., 2005; Fogel and Smith, 2006; Schabus et al., 2006; Fogel et al., 2007; Peters et al., 2007, 2008; Tucker and Fishbein, 2009; Lustenberger et al., 2012; Ujma et al., 2014; Ward et al., 2014; Ujma et al., 2015a; Fang et al., 2017a,b, 2019; Guadagni et al., 2021) from which one study (Guadagni et al., 2021) and one sub-study (Peters et al., 2008) were performed in healthy aged adults with a total sample size of 77 (38 females, 49% females, Supplementary Tables 31, 32).

#### 3.3.1 Sleep spindle properties

The relationship between cognitive abilities and sleep spindle amplitude was only available in one study using adult subjects (Fang et al., 2019), and revealed a positive correlation (*n* = 27, *r* = 0.435, p_*r* =_ 0.002, 59% females, Supplementary Tables 33, 36).

**Figure 10:**
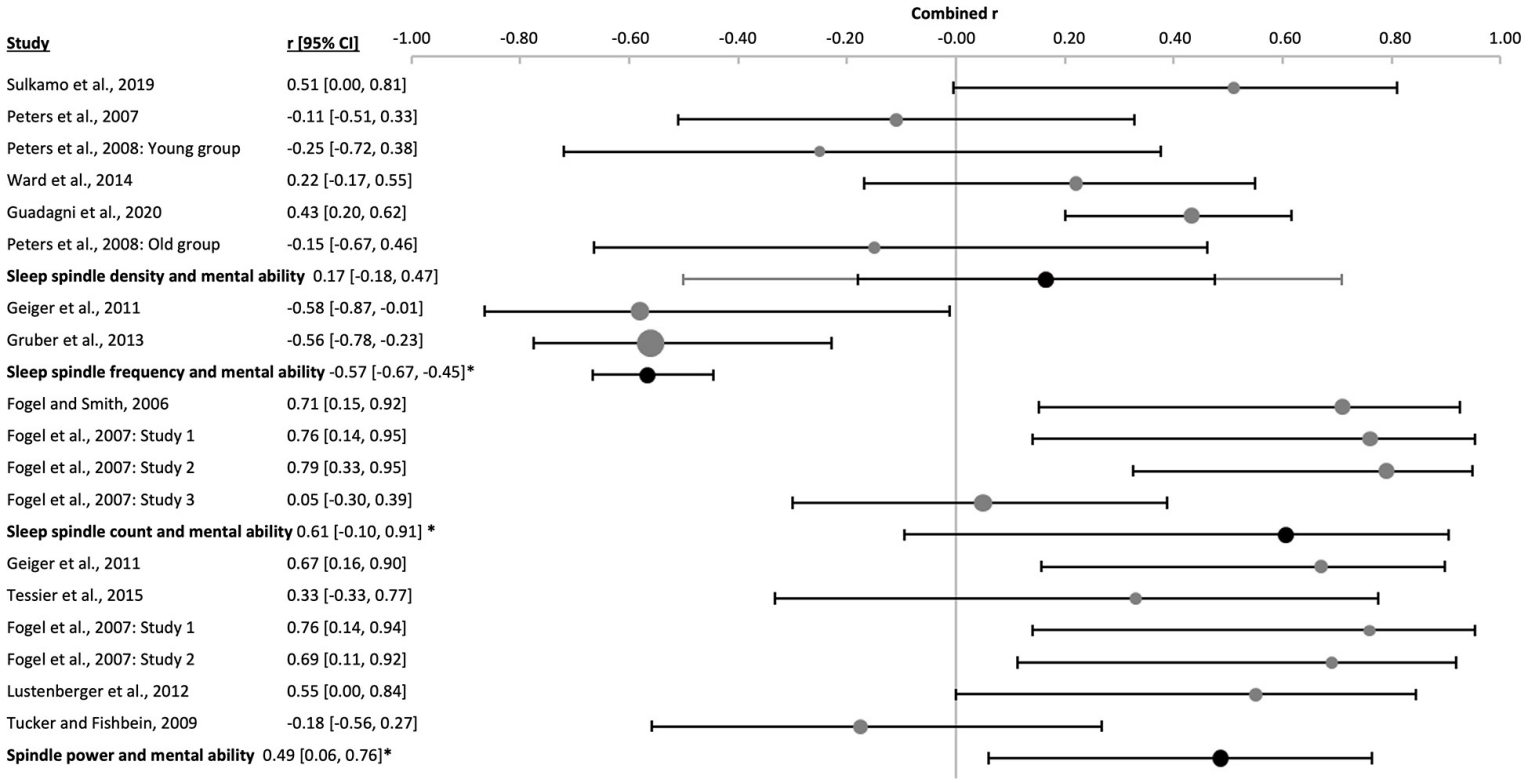
Forest plot of the correlation between sleep spindle properties and cognitive ability in all ages. Individual correlation coefficients (Fisher's z-transformed, gray dots) and the 95% confidence intervals (black lines) are shown for each study, followed by their combined correlation coefficients (Fisher's z-transformed, black dot), the combined confidence intervals (black line) and the prediction interval (gray line) using the random-effects model. The size of the dots represents the weight. Positive and negative values indicate the direction of the correlations. *p_r_ ≤ 0.05.

**Figure 11:**
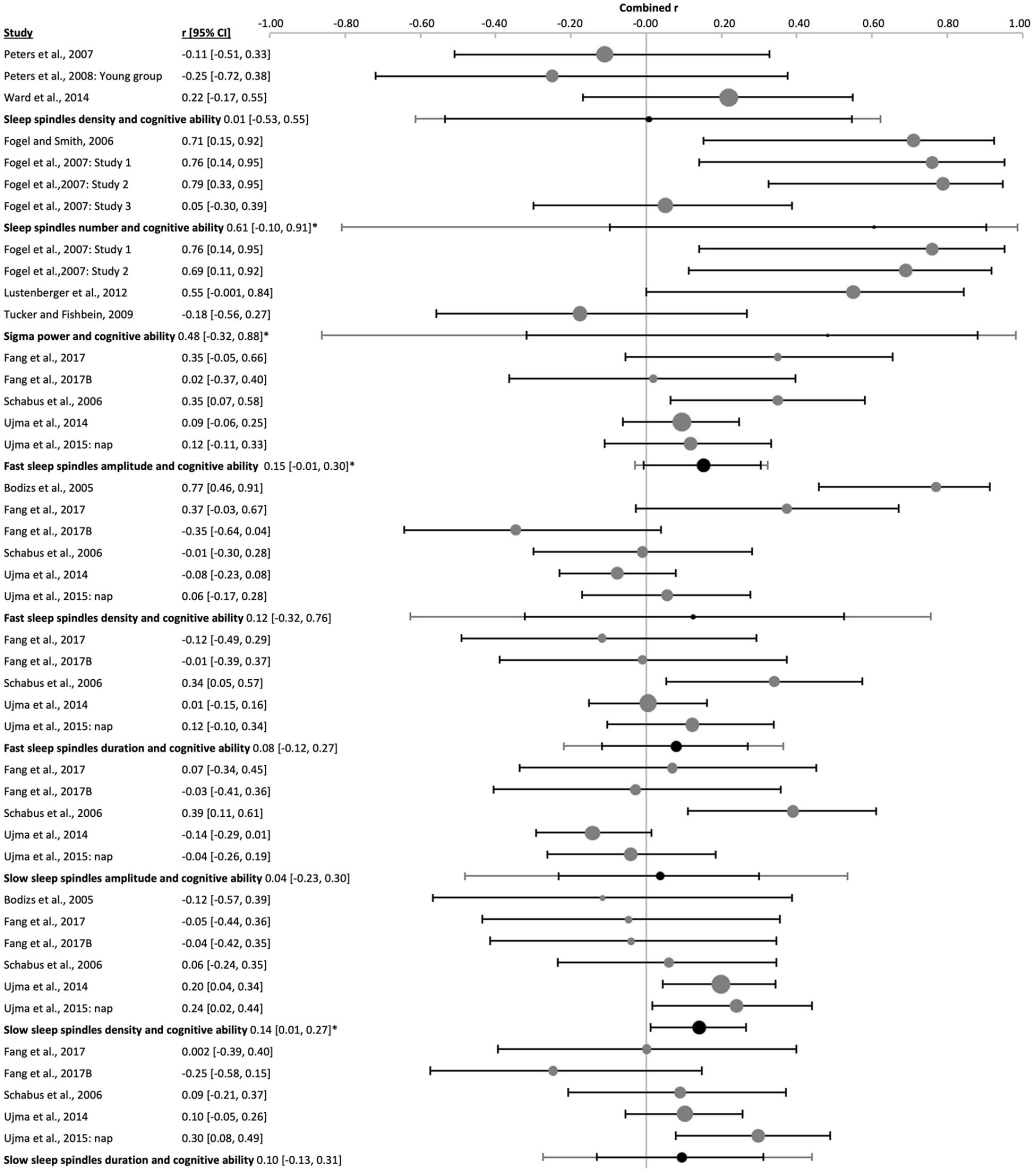
Forest plot of the correlation between sleep spindle properties and cognitive ability in adults. The individual correlations (Fisher's z-transformed, gray dots) and the 95% confidence intervals (black line) are shown for each study, followed by the combined correlation (Fisher's z-transformed, black dot), the combined confidence intervals (black line) and the prediction interval (gray line) using the random-effects model. The size of the dots represents the weight. Moreover, the combined correlation separated by age groups is showed on the right side. Positive and negative correlations indicate the direction, and the magnitude indicates the strength of the relation between cognitive abilities and the corresponding sleep spindle parameter. *p_r_ ≤ 0.05.

The combined correlation effect of EEG spindle power with cognitive ability for all ages was also highly significant and positive [k = 6, *n* = 88, *r* = 0.49 (0.06, 0.76), p_*r* =_ 0.004, *I*^2^ = 63.42%, Egger's regression's *p* = 0.02, Figure 10 and Supplementary Table 33]. Two of the studies were performed in children [k = 2, *n* = 27, *r* = 0.53 (−0.98, 1), p_*r* =_ 0.012, *I*^2^ = 12.80%, publication bias's p= n.s., Supplementary Table 34] and the rest in adults [k = 4, *n* = 61, *r* = 0.48 (−0.32, 0.88), p_*r* =_ 0.05, *I*^2^ = 74.67%, Figure 11 and Supplementary Table 36]. Interestingly, Egger's regression test indicating funnel plot asymmetry, was significant for the whole data set and for the adult subgroup (*p* = 0.04), whereas Begg and Mazumbar's rank correlation test reached significance only in the adult subgroup (*p* = 0.042). Thus, particularly among studies including adults publication bias is indicated (Supplementary Tables 33, 34, 36 and Supplementary Figure 6). The influence of females in these studies was low (*R*^2^ = 14.76%, P_z_ = n.s., Supplementary Table 40 and Supplementary Figure 7). Sensitivity analysis accounting for the use an approximated Pearson correlation coefficient derived from a Spearman's correlation coefficient (Geiger et al., 2011) yielded comparable results [k = 5, *n* = 74, *r* = 0.45 (−0.11, 0.79), p_*r* =_ 0.025, *I*^2^ = 66.25%] yet did not eliminate the risk of publication bias (Egger's regression's *p* = 0.03, Begg and Mazumbar's *p* = 0.05, Supplementary Table 38).

Information on sleep spindle density was available in six studies (k = 6, *n* = 162, Figure 10, Supplementary Table 33), but the combined correlation was non-significant [*r* = 0.17 (−0.18, 0.47), p_*r* =_ 0.220, *I*^2^ = 56.11%]. Three of these studies were performed in adults [k = 3, *n* = 68, *r* = 0.01 (−0.53, 0.55), p_*r* =_ 0.957, *I*^2^ = 13.43%, Figure 11 and Supplementary Table 36] and two in healthy aged subjects [k = 2, *n* = 77, *r* = 0.21 (−1, 1), p_*r* =_ 0.472, *I*^2^ = 71.42%, Supplementary Table 37], both yielding non-significant combined correlations with cognitive ability. The only study with a significant correlational effect size for spindle density with cognitive ability (*n* = 17, *r* = 0.51, p_*r* =_ 0.035, Supplementary Table 34) was performed in children. Sensitivity analysis eliminating the approximated Pearson correlation from a Spearman's rank correlation coefficient (Sulkamo et al., 2019) increased the risk of publication bias [k = 5, *n* = 145, *r* = 0.10 (−0.28, 0.45), p_*r* =_ 0.478, *I*^2^ = 59.18%, Egger's regression's *p* = 0.03, Supplementary Table 38].

In line with the results for children, sleep spindle count revealed a significant combined positive correlation with cognitive ability [k = 4, *n* = 69, *r* = 0.61 (−0.10, 0.91), p_*r* =_ 0.005, *I*^2^ = 75.80%, Figures 10, 11, Supplementary Tables 33, 36]. Egger's regression was significant (*p* = 0.04) since one study showed a different result from the others (Supplementary Table 33 and Supplementary Figure 6).

Interestingly, for sleep spindle frequency, based on data of two studies with children, a combined negative correlation was found indicating lower sleep spindle frequency with higher cognitive ability scores (Geiger et al., 2011; Gruber et al., 2013); [k = 2, *n* = 43, *r* = −0.57 (−0.67, −0.45), p_*r* =_ 0.0000, *I*^2^ = 0.00%, Egger's *p* = 0.317; Figure 10 and Supplementary Tables 33, 34]. Sensitivity analysis eliminating the approximated Pearson correlation from Spearman's correlation (Geiger et al., 2011) did not have an impact on the results (*n* = 29, *r* = −0.56, p_*r* =_ 0.001; Supplementary Table 38).

The proportion of female participants influenced significantly the relationship between cognitive ability and sleep spindle count (P_z_ = 0.0005, *R*^2^ = 98.76%; Supplementary Figure 7 and Supplementary Table 40); however, this result is to be treated cautiously, as nearly all subjects here were females.

#### 3.3.2 Fast sleep spindle properties

**Figure 12:**
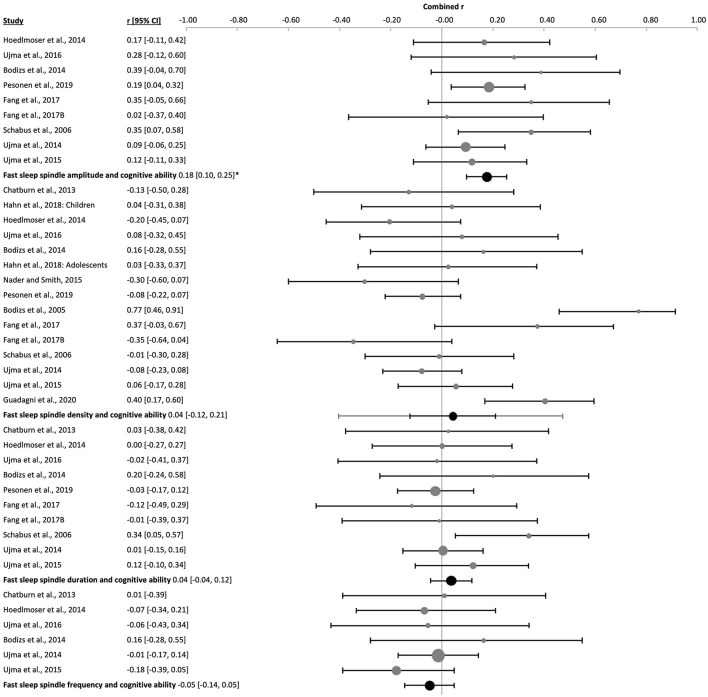
Forest plot of the correlation between fast sleep spindle properties and cognitive ability in all ages. The individual correlation coefficients (Fisher's z-transformed, gray dots) and the 95% confidence intervals (black line) are shown for each study, followed by the combined correlation coefficient (Fisher's z-transformed, black dot), the combined confidence intervals (black lines) and the prediction interval (gray line) using the random-effects model. The size of the dots represents the weight. Positive and negative correlation coefficients indicate the direction, and the magnitude indicates the strength of the relation between cognitive abilities and the corresponding sleep spindle parameter. *p_r_ ≤ 0.05.

The amplitude of fast sleep spindles correlated positively with cognitive ability [k = 9, *n* = 625, *r* = 0.18 (0.10, 0.25), p_*r* =_ 4 x 10^−7^, *I*^2^ = 0.00%, Figure 12 and Supplementary Table 33 and Supplementary Figure 6]. Two studies used children [k = 2, *n* = 82, p_*r* =_ 0.21 (−0.47, 0.73), p_*r* =_ 0.0002, *I*^2^ = 0.00%, Supplementary Table 34], two studies used adolescents [k = 2, *n* = 200, *r* = 0.21 (−0.58, 0.80), p_*r* =_ 0.002, *I*^2^ = 0.00%, Supplementary Table 35] and five studies used adult subjects [k = 5, *n* = 343, *r* = 0.15 (−0.01, 0.30), p_*r* =_ 0.008, *I*^2^ = 5.13%, Figure 11 and Supplementary Table 36]. There was no risk of publication bias (Supplementary Tables 33–37 and Supplementary Figure 6). No significant change appeared when eliminating the nap study [k = 8, *n* = 546, *r* = 0.18 (0.09, 0.27), p_*r* =_ 2 x 10^−6^, *I*^2^ = 0.00%, Supplementary Table 39]. The percentage of females had little influence on these results (*R*^2^ = 10.88%, p_z_ = n.s., Supplementary Table 40 and Supplementary Figure 7).

The combined density of fast sleep spindles was not significantly correlated with cognitive ability [k = 15, *n* = 834, *r* = 0.04 (−0.12, 0.21), p_*r* =_ 0.577, *I*^2^ = 67.57%, publication bias= n.s., Figure 12, Supplementary Table 33, and Supplementary Figure 6]. Of these studies, four were performed in children [k = 4, *n* = 143, *r* = −0.08 (−0.30, 0.14), p_*r* =_ 0.259, *I*^2^ = 0.00%, Figure 14 and Supplementary Table 34], four in adolescents [k = 4, *n* = 266, *r* = −0.07 (−0.28, 0.14), p_*r* =_ 0.300, *I*^2^ = 4.78%, Figure 15 and Supplementary Table 35], and six in adults [k = 6, *n* = 362, *r* = 0.12 (−0.32, 0.76), p_*r* =_ 0.487, *I*^2^ = 79.83%, Figure 11 and Supplementary Table 36]. In the study with older subjects a significant correlation yielding increased fast spindle density with higher cognitive ability was measured (k = 1, *n* = 63, *r* = 0.40, p_*r* =_ 0.001, Supplementary Table 37). Publication bias was not observed in any age group (Supplementary Tables 33–37 and Supplementary Figure 6). Sensitivity analyses were performed eliminating the study with an approximated Pearson correlation coefficient (Hahn et al., 2018) as well as the nap study (Ujma et al., 2015a) resulting in very similar results [k = 13, *n* = 766, *r* = 0.05 (−0.15, 0.24), p_*r* =_ 0.603; and k = 14, *n* = 755, *r* = 0.04 (−0.14, 0.23), p_*r* =_ 0.604, respectively, Supplementary Tables 38, 39]. The percentage of females did not statistically influence results (*R*^2^ = 4.00%, p_z_ = n.s., Supplementary Table 40 and Supplementary Figure 7).

The duration of fast sleep spindles was analyzed in relation to cognitive ability in ten studies with no significant effect [k = 10, *n* = 652, *r* = 0.04 (−0.04, 0.12), p_*r* =_ 0.303, *I*^2^ = 0.00%, Figure 12, Supplementary Table 33, and Supplementary Figure 6]. Three studies used children [k = 3, *n* = 109, *r* = 0.002 (−0.05, 0.05), p_*r* =_ 0.892, *I*^2^ = 0.00%, Figure 14 and Supplementary Table 34], two used adolescents [k = 2, *n* = 200, *r* = −0.0002 (−0.72, 0.72), p_*r* =_ 0.997, *I*^2^ = 0.00%, Supplementary Table 35] and five used adults [k = 5, *n* = 343, *r* = 0.08 (−0.12, 0.27), p_*r* =_ 0.265, *I*^2^ = 27.71%, Figure 11 and Supplementary Table 36]. No risk of publication bias was observed (Supplementary Tables 33–36 and Supplementary Figure 6). Deleting the nap study from the analyses did not have any impact [k = 9, *n* = 573, *r* = 0.03 (−0.06, 0.11), p_*r* =_ 0.520, *I*^2^ = 0.00%, Supplementary Table 39]. The percentage of females did not influence results (*R*^2^ =14.00%, p_z_ = n.s., Supplementary Table 40 and Supplementary Figure 7).

The combined effect of correlating mean frequency of fast sleep spindles with cognitive ability scores was also not significant [k = 6, *n* = 372, *r* = −0.05 (−0.14, 0.05), p_*r* =_ 0.206, *I*^2^ = 0.00%, publication bias = n.s., Figure 12, Supplementary Table 33, and Supplementary Figure 6). The same result was obtained when using only the data of adolescents (k = 1, *n* = 24, *r* = 0.16, p_*r* =_ 0.451, Supplementary Table 35) or adults separately [k = 2, *n* = 239, *r* = −0.08 (−0.8, 0.74), p_*r* =_ 0.341, *I*^2^ = 29.06%, Supplementary Table 36]. However, a negative correlation was reached for children [k = 3, *n* = 109, *r* = −0.05 (−0.14, 0.05), p_*r* =_ 0.045, *I*^2^ = 0.00%, Figure 14 and Supplementary Table 34]. For the combined effect eliminating the one nap study did not affect results [k = 5, *n* = 293, *r* = −0.01 (−0.09, 0.07), p_*r* =_ 0.669, Supplementary Table 39]. The percentage of females did not influence the results (*R*^2^ = 67.18%, p_z_ 0.190, Supplementary Table 40 and Supplementary Figure 7), however, this effect may be biased by one study using only males (Ujma et al., 2015a).

The correlation between EEG power in the fast spindle frequency band and cognitive ability was only analyzed in one study in children (k = 1, *n* = 13, *r* = 0.33, p_*r* =_ 0.278, 0% females, Supplementary Tables 33, 34).

#### 3.3.3 Slow sleep spindle properties

**Figure 13:**
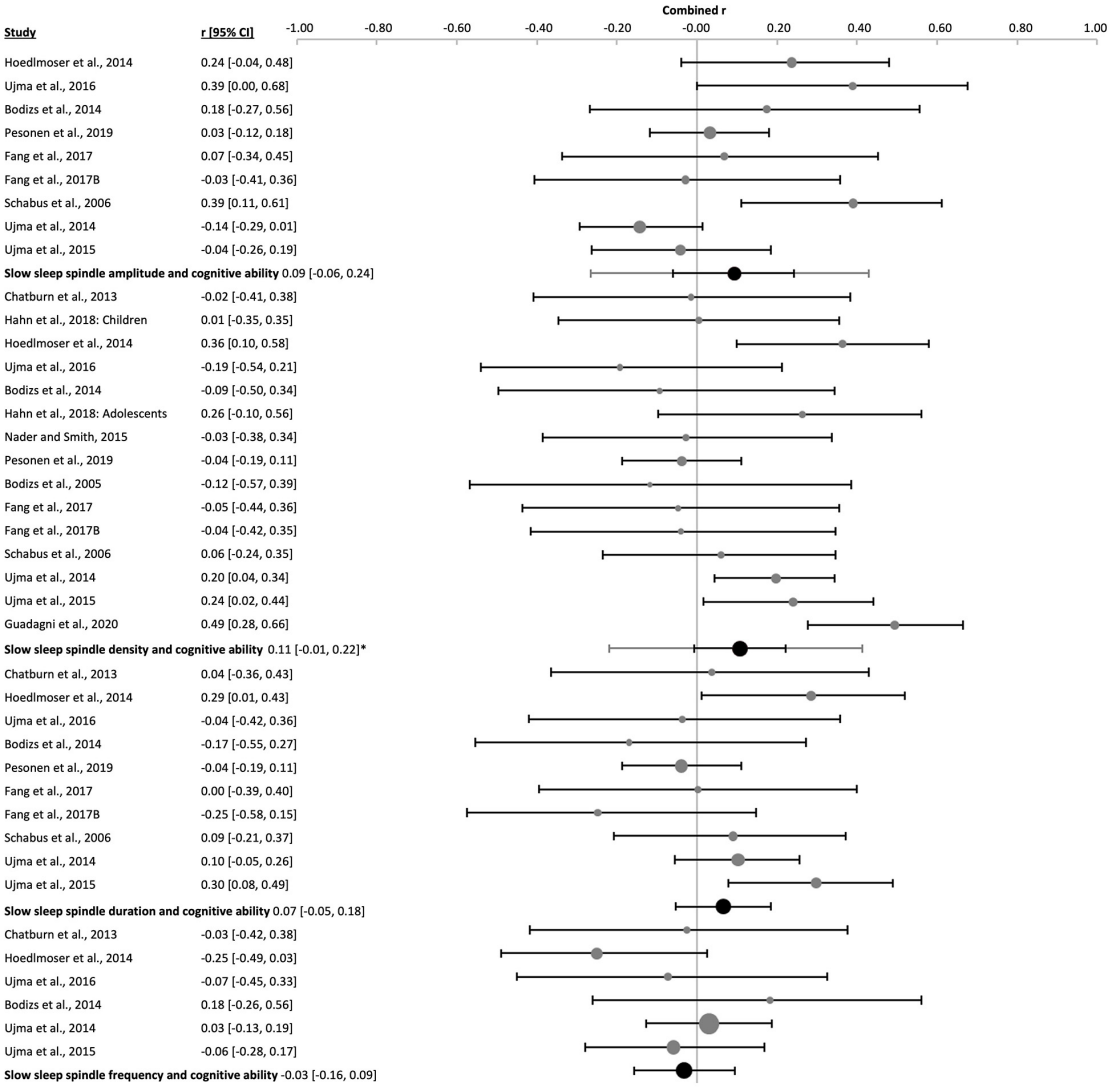
Forest plot of the correlation between slow sleep spindle properties and cognitive ability scores in all ages. The individual correlations (Fisher's z-transformed, gray dots) and the 95% confidence intervals (black lines) are shown for each study, followed by the combined correlation coefficient (Fisher's z-transformed, black dot), the combined confidence intervals (black line) and the prediction interval (gray line) using the random-effects model. The size of the dots represents the study or combined weight, respectively. Positive and negative correlations indicate the direction, and the magnitude indicates the strength of the relation between cognitive abilities and the corresponding sleep spindle parameter. *p_r_ ≤ 0.05.

The combined averaged correlation between the amplitude of the slow sleep spindles and cognitive ability was not significant [k = 9, *n* = 625, *r* = 0.09 (−0.06, 0.24), p_*r* =_ 0.160, *I*^2^ = 55.75, no publication bias, Figure 13, Supplementary Table 33, and Supplementary Figure 6]. Reflecting results for the two adolescent [k = 2, *n* = 200, *r* = 0.05 (−0.48, 0.55), p_*r* =_ 0.289, *I*^2^ = 0.00%, Supplementary Table 35] and five adult studies [k = 5, *n* = 343, *r* = 0.04 (−0.23, 0.30), p_*r* =_ 0.711, *I*^2^ = 63.73%, Figure 11 and Supplementary Table 36]. However, the two studies in children provided a positive correlation, as was observed for fast spindles at all ages [k = 2, *n* = 82, *r* = 0.29 (−0.61, 0.86), p_*r* =_ 0.0002, *I*^2^ = 0.00%, Supplementary Table 34]. Deleting the nap study with adult subjects from the meta-analysis had a minor impact on the combined effect [k = 8, *n* = 546, *r* = 0.12 (−0.05, 0.28), p_*r* =_ 0.104, *I*^2^ = 59.93%, Supplementary Table 39]. The percentage of females had no influence on the results (*R*^2^ = 7.14%, p_z_ = n.s., Supplementary Table 40 and Supplementary Figure 7).

**Figure 14:**
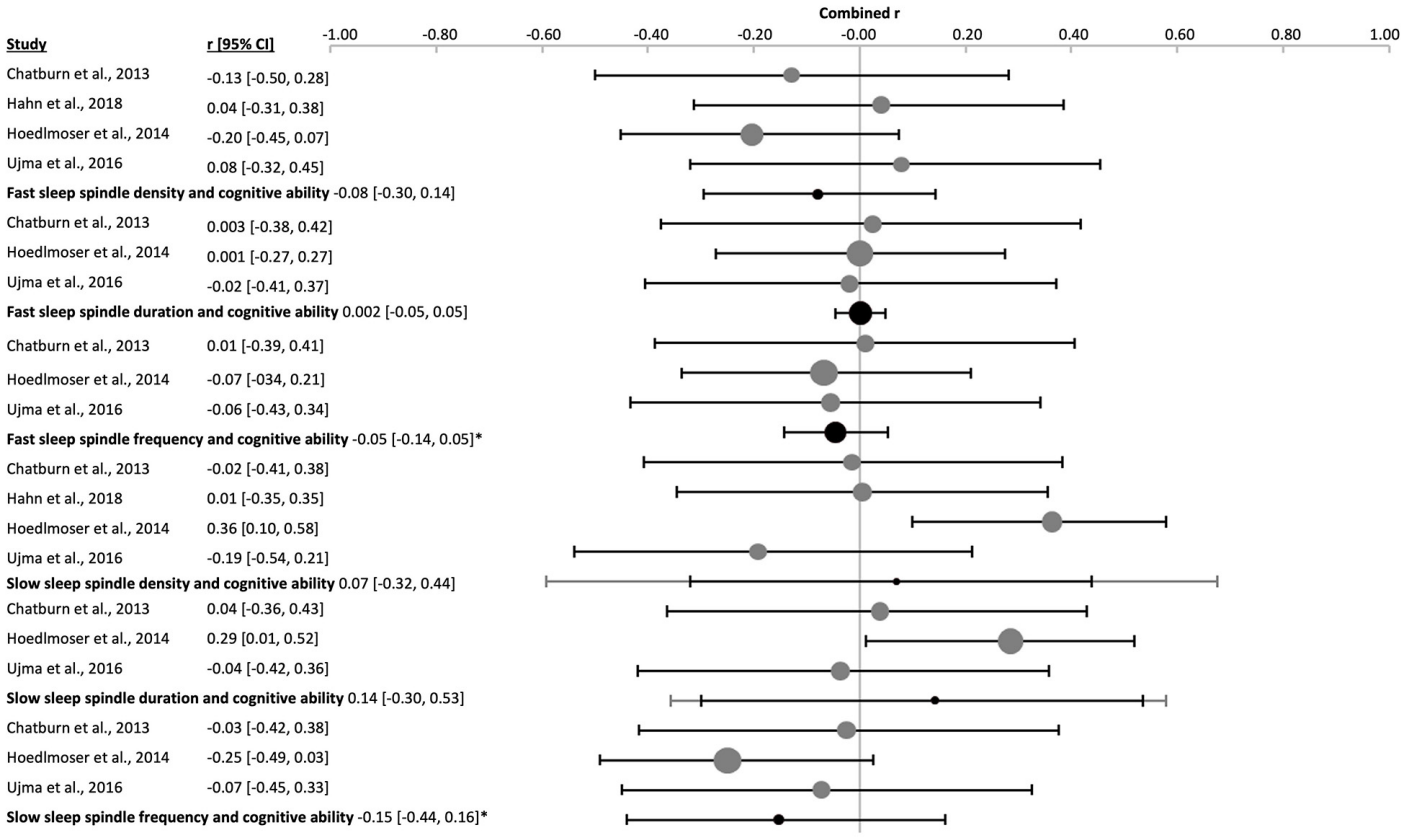
Forest plot of the correlation between sleep spindle properties and cognitive ability in children. The individual correlations (Fisher's z-transformed, gray dots) and the 95% confidence intervals (black line) are shown for each study, followed by the combined correlation (Fisher's z-transformed, black dot), the combined confidence intervals (black line) and the prediction interval (gray line) using the random-effects model. The size of the dots represents the weight. Moreover, the combined correlation separated by age groups is shown on the right side. Positive and negative correlation coefficients indicate the direction, and the magnitude indicates the strength of the relation between cognitive abilities and the corresponding sleep spindle parameter. *p_r_ ≤ 0.05.

**Figure 15:**
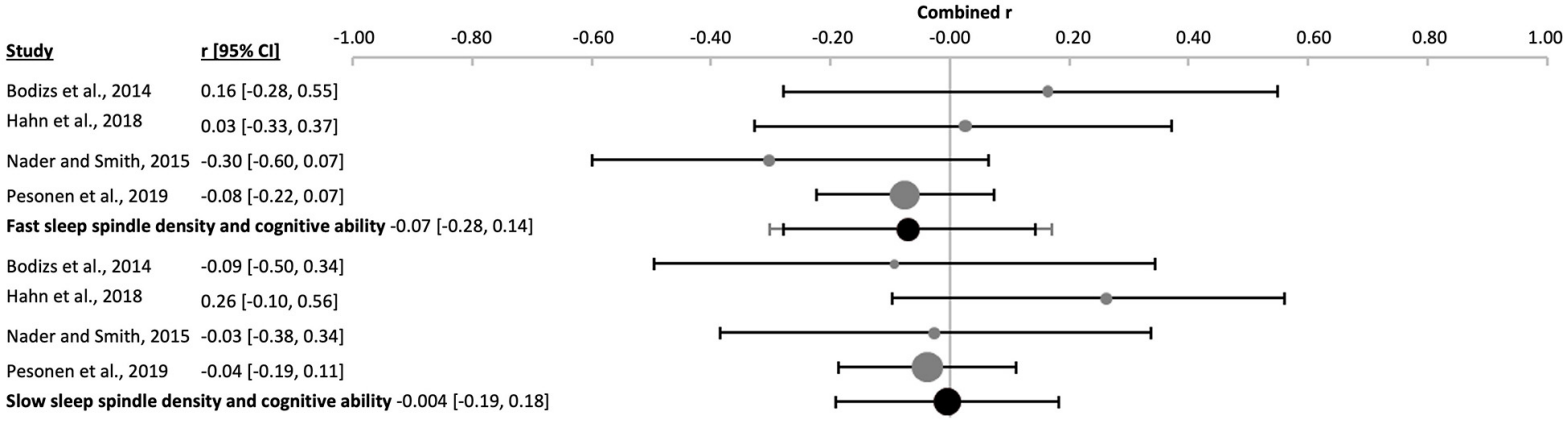
Forest plot of the correlation between sleep spindle properties and cognitive ability in adolescents. The individual correlations (Fisher's z-transformed, gray dots) and the 95% confidence intervals (black line) are shown for each study, followed by the combined correlation (Fisher's z-transformed, black dot), the combined confidence intervals (black line) and the prediction interval (gray line) using the random-effects model. The size of the dots represents the weight. Moreover, the combined correlation separated by age groups is shown on the right side. Positive and negative correlations indicate the direction, and the magnitude indicates the strength of the relation between cognitive abilities and the corresponding sleep spindle parameter.

The combined correlation of slow spindle density with cognitive ability scores reached significance [k = 15, *n* = 834, *r* = 0.11 (−0.01, 0.22), p_*r* =_ 0.044, *I*^2^ = 55.75%, Egger's regression's *p* = n.s., Begg and Mazumbar's *p* = 0.015, Figure 13, Supplementary Table 33, and Supplementary Figure 6]. However, this effect was not consistent across age groups, but rather reflected results of the six studies with adults [k = 6, *n* = 362, *r* = 0.14 (0.01, 0.27), p_*r* =_ 0.005, *I*^2^ = 0.00%, Egger's regression's *p* = 0.01., Begg and Mazumbar's *p* = 0.039, Figure 11 and Supplementary Table 36] and healthy aged subjects (k = 1, *n* = 63, *r* = 0.49, p_*r* =_ 0.00, Supplementary Table 37). Neither the studies with children [k = 4, *n* = 143, *r* = 0.07 (−0.32, 0.44), p_*r* =_ 0.582, *I*^2^ = 55.63 %, no publication bias, Figure 14 and Supplementary Table 34], nor adolescents [k = 4, *n* = 266, *r* = −0.004 (−0.19, 0.18), p_*r* =_ 0.947, *I*^2^ = 0.00%, no risk of publication bias, Figure 15 and Supplementary Table 35) revealed a combined linear correlation.

The percentage of females did not drive the association between slow spindle density and cognitive ability scores (*R*^2^ = 6.47%, p_z_ = 0.351, Supplementary Table 40 and Supplementary Figure 7). After eliminating the study for which an approximated Pearson coefficient was used (Hahn et al., 2018) the combined correlation was only a trend, yet the risk of publication bias was removed [k = 13, *n* = 766, *r* = 0.10 (−0.03, 0.23), p_*r* =_ 0.090, *I*^2^ = 56.40%, publication bias n.s., Supplementary Table 38]. Omitting the nap study (Ujma et al., 2015a) had a similar effect [k = 14, *n* = 755, *r* = 0.09 (−0.03, 0.21), p_*r* =_ 0.102, *I*^2^ = 52.38%, Begg and Mazumbars's *p* = 0.019, Supplementary Table 39].

Slow sleep spindle duration had no significant combined correlation with cognitive ability [k = 10, *n* = 652, *r* = 0.07 (−0.05, 0.18), p_*r* =_ 0.210, *I*^2^ = 32.75%, no publication bias, Figure 13, Supplementary Table 33, and Supplementary Figure 6], as also found separately for children [k = 3, *n* = 109, *r* = 0.14 (−0.30, 0.53), p_*r* =_ 0.172, *I*^2^ = 9.53%, Supplementary Table 34], adolescents [k = 2, n =200, *r* = −0.05 (−0.52, 0.44), p_*r* =_ 0.195, *I*^2^ = 0.00%, Supplementary Table 35] and adults [k = 5, *n* = 343, *r* = 0.10 (−0.13, 0.31), p_*r* =_ 242, *I*^2^ = 39.93%, Supplementary Table 36]. Eliminating the nap study from the meta-analysis did not affect results [k = 9, *n* = 573, *r* = 0.03 (−0.07, 0.14), p_*r* =_ 0.455, low heterogeneity, Supplementary Table 39]. The percentage of females explained half of the heterogeneity and showed an influence on our results (*R*^2^ = 56.05%, p_z_ = 0.006, Supplementary Table 40 and Supplementary Figure 7).

Mean slow spindle frequency had no significant combined correlation with cognitive ability [k = 6, *n* = 372, *r* = −0.03 (−0.16, 0.09), p_*r* =_ 0.518, *I*^2^ = 0.00%, no publication bias, Figure 13, Supplementary Table 33, and Supplementary Figure 6], as also found separately for adolescents (k = 1, *n* = 24, *r* = 0.18, p_*r* =_ 0.399, Supplementary Table 35) and adults [k = 2, *n* = 239, *r* = 0.002 (−0.49, 0.49), p_*r* =_ 0.969, *I*^2^ = 0.00%, Supplementary Table 36]. However, studies in children revealed a positive combined correlation of slow spindle frequency with cognitive ability score [k = 3, *n* = 109, *r* = −0.15 (−0.44, 0.16), p_*r* =_ 0.036, *I*^2^ = 0.00%, Egger's regression's *p* = 0.07, Begg and Mazumbar's *p* = n.s., Supplementary Table 34]. Eliminating data of the nap study consisting only of male subjects (k = 1, *n* = 79, *r* = −0.059, p_*r* =_ 0.196) from the combined correlation analyses did not impact those results [k = 5, *n* = 293, *r* = −0.03 (−0.20, 0.15), p_*r* =_ 0.668, *I*^2^ = 4.21%, Supplementary Table 39]. The percentage of females had no influence on the outcome (*R*^2^ = 1.43%, p_z_ = n.s., Supplementary Table 40 and Supplementary Figure 7).

The correlation between EEG power of the slow sleep spindle frequency band and a cognitive ability score was only analyzed in one study in children (k = 1, *n* = 13, *r* = 0.27, p_*r* =_ 0.381, 0% females, Supplementary Tables 33, 34).

## 4 Discussion

The need to fully understand the mechanisms and the function of sleep, its implication for learning and memory, as well as how to modulate those processes is increasing. That interest may be explained by the high amount of sleep disorders and mental diseases affecting in a negative fashion the increased cognitive requirements needed for the daily life in humans nowadays. Sleep spindles are thalamo-cortical oscillatory events (burst of 0.5–3 s) that appear during NREM sleep (mostly stage 2) and are highly associated with learning and memory consolidation (Gais et al., 2002; Marshall et al., 2006; Mölle et al., 2009, 2011; Fernandez and Luthi, 2020; Dehnavi et al., 2021). Since, sleep spindles can be different across individuals (Koo and Marshall, 2016; Ujma, 2018; Gonzalez et al., 2022; Poirson et al., 2024), this meta-analysis describes the effect of age, sex, and cognitive ability in six parameters of sleep spindles (fast and slow spindles within the frequency range of 11 to 16 Hz, together and separate) and when available, describes the effects of age and sex in eleven PSG parameters in three different meta-analyses.

### 4.1 Differences in sleep spindles and PSG in humans—effects of age

The significant findings in the meta-analysis regarding the age-dependent differences in sleep spindles were the robust decrease in sleep spindle density, amplitude and duration in older subjects compared to young adults. Decreased spindle density was thereby maintained in separate analyses for fast and slow spindles. Sleep spindles properties were measured mostly but not exclusively during S2 (Supplementary Table 9), whereby the combined difference in time spent in N2 did not differ significantly across ages. Regarding sleep macrostructure older subjects (≥ 60 years) spent significantly more time in N1 and less time in deeper NREM and REM sleep compared to younger subjects ( ≤ 35 years). Furthermore, sleep efficiency was poorer, TST reduced and both sleep latency and WASO increased in older as compared to younger subjects.

Despite including only those studies on sleep spindle events that also characterized age (k = 14), our PSG results for subjects from young adulthood until ~ 80 years are remarkably similar to larger meta-analyses that only included normative PSG data in 65 studies from which we shared 2 studies (Ohayon et al., 2004) and in 169 studies from which we didn't share included studies (Boulos et al., 2019). Note, a more recent study distinguishing between medicated older subjects above and below 75 years reported some distinctions also between N2 and N3 sleep (Poirson et al., 2024).

The generation and expression of spindles is not only dependent on the thalamus, but MRI studies in indicate activation in the cingulate, precuneus and hippocampus amongst other structures. Thalamic volume and the integrity of thalamo-cortical projections were found to decrease across adults from 20 to 85 years (Fama and Sullivan, 2015; Cao et al., 2024). Decreases in cortical gray and white matter with age may result in decreased neuronal synchrony and decrement in the spindle properties (Crowley et al., 2002; Clawson et al., 2016). Age-related decline of hippocampal volume, a subcortical region also activated in relation to sleep spindles (Goodro et al., 2012) was likewise observed. Furthermore, a decrease in the conductivity because of thicker bone and increased distance between from brain to scalp due to the reduced brain volume can be related to the reduced EEG amplitude seen in elderly people (Crowley et al., 2002; Mander et al., 2013). Employing a realistic head model and response to somatosensory evoked responses Antonakakis et al. found age and skull thickness were significant predictors of skull conductivity (Antonakakis et al., 2020).

Poorer sleep macrostructure with age is attributed to structural and neuromodulatory changes, as well as circadian disorganization as reviewed in detail (Zhong et al., 2019; Campos-Beltran and Marshall, 2021).

### 4.2 Differences in sleep spindles and sleep EEG in humans—effects of sex

In the present meta-analyses, the comparison between sexes across all ages revealed in a combined analysis reduced spindle power in males compared to females with the older subjects droving this effect. Results on sex-dependent effects on spindle amplitude or power are inconsistent, with many studies finding increased values in females and others lacking any differential effect (Fernandez and Luthi, 2020; Markovic et al., 2020; Eggert et al., 2021; Chapman et al., 2025). Reports on larger wake oscillatory activity in females and more pronounced in elderly (Han et al., 2025) as well increments over a wide range of frequencies led to the concept that biological as well as non-biological mechanisms unrelated to spindle generation may underly the sex-dependent effects (Carrier et al., 2017; Chapman et al., 2025). Indeed, old age may facilitate such disparities in neural dynamics (Han et al., 2025) as our results here and in section 4.1 indicate. It is to note, that our analysis includes age groups before and after menopause, after which progesterone and estrogen levels in females are decreased. In contrast in aging men testosterone levels gradually decrease. Progesterone metabolites increase GABA-A receptor signaling relevant for sleep spindle generation and sleep onset. It cannot be ruled out that this relationship contributes to sex-differences with aging (Lancel et al., 1996; Baker et al., 2019). Finally, effects on EEG signals of age, sex and other demographic factors appear inter-dependent. Applying a multivariate learning approach Ujma and colleagues found that both age (*r* = 0.6) and sex (*r* = 0.5) could be predicted from the EEG envelope spectrum (Ujma et al., 2022).

Regarding PSG measures, sleep in men was often poorer, articulated by different parameters dependent upon age: On average, young male subjects revealed lower sleep efficiency, less TST, more WASO and S1 at the cost of time in S2. Middle-aged men also revealed lower sleep efficiency, yet conjoint with increased sleep latency and decreased REM sleep; older male subjects revealed reduced deep NREM sleep together with increased NREM S1 sleep. Yet, these differences between sexes were moderate to small. Closely similar results indicating poorer sleep in males were found in the meta-analyses of Ohayon et al. (2004), and summarized by Carrier et al. (2017), but not in another meta-analyses (Boulos et al., 2019).

### 4.3 Differences in sleep spindles in humans—effects of cognitive abilities

Overall, the meta-analyses yielded often differential results between sleep spindle properties and cognitive ability dependent upon sleep spindle frequency and age. A positive correlation of spindle power with cognitive ability was determined for spindles independent of frequency and for fast spindles, but not for slow spindles. There was no correlation between spindle duration and cognitive ability. This supports the concept that post-training differences in spindle duration are truly a task-dependent effect and are less likely to be mediated by cognitive ability. However, the cognitive tasks included in this meta-analysis spanned multiple domains, including reasoning ability, general intelligence, executive function, and processing speed (See Supplementary Table 25 for children, Supplementary Table 27 for adolescents, Supplementary Table 29 for adults, and Supplementary Table 31 for older adults).

The relationship with cognitive ability of spindle density and spindle frequency revealed age-dependent distinctions. However, the relatively limited number of studies warrants caution regarding generalizability. Our meta-analyses showed that specifically slow spindle density correlated positively with cognitive ability, an effect attributed to middle-aged and older adults. For children, a positive correlation with cognitive ability was found only for spindle count and density in studies not discriminating between fast and slow activity. This may be due to mean spindle frequency in children and adolescents undergoing strong developmental changes (Kwon et al., 2023). Along these lines, for children, fast spindle frequency correlated negatively, and slow spindle frequency positively with cognitive ability based on data of the same 3 studies, while data of 2 other studies not discriminating between spindle frequency bands likewise revealed a negative correlation. Mean spindle frequency did not correlate with cognitive ability for adults or adolescents. The difference observed in children, compared to adults, in how slow and fast spindles relate to cognitive ability likely reflects a stronger functional tuning between spindle frequency generators (e.g., thalamic reticular nucleus, thalamocortical loops and the GABAergic system) and the neural processes supporting cognitive ability.

In comparison to a previous meta-analysis by Ujma (2018) only one result differed. Ujma (2018) found a non-significantly negative correlation to fast sleep spindle density, whereas we found a positive correlation with slow spindle density, for all ages combined as well as for adults and in old subjects. The difference is most likely attributed to the additional inclusion in our analyses of data from 5 recent studies (Hahn et al., 2018; Fang et al., 2019; Pesonen et al., 2019; Sulkamo et al., 2019; Guadagni et al., 2021). The meta-analysis by Reynolds et al. (2018) is less directly comparable, as it specifically differentiated between cognitive domains and examined their individual relationships with sleep spindles in adolescents (Reynolds et al., 2018). They reported a stronger relationship to spindles for fluid intelligence.

### 4.4 Limitations

The main limitation of this meta-analysis is the low number of studies that showed or investigated sufficient data for inclusion into our calculations. In fact, most of the studies' main topic was not to investigate differences in age or between sexes. In addition, the lack of sleep spindle measurements in numerous older studies ultimately led to their exclusion. Furthermore, the distinction between spindle types is not trivial. The present meta-analyses focused on slow and fast sleep spindles within the 11–16 Hz range. Yet over time, as we were also initial to show, slow frontal sleep spindles emerge around 9 Hz in SWS (Mölle et al., 2011), and using phase of occurrence relative to the slow oscillation as well as frequency McConnel and colleagues distinguished between 3 spindle types in humans (Mcconnell et al., 2021). A further unaddressed aspect are relative topography shifts (e.g., anterior-posterior) in spindle characteristics across the investigated age groups, most pronounced for spindle amplitudes (Martin et al., 2013). When it comes to cognitive abilities, studies fulfilling our meta-analyses criteria rarely correlated PSG parameters with cognitive ability scores, more frequently with memory consolidation. For the former we recommend a designated review by Ujma et al. (2020). The cognitive ability in this meta-analysis varied across studies, which may have contributed to heterogeneity in the reported associations. Furthermore, most of the studies that were included showed high heterogeneity, and the results must be interpreted accordingly. An additional source of variability across studies may arise of the differences in spindle detection methodologies, for instance using root mean square vs. wavelet detection or fixed vs. individually adjusted thresholds (Warby et al., 2014; Ujma et al., 2015b). Another limitation arose from the fact that studies often did not publish non-significant results. Fortunately, some authors provided this information per email, else data could not have been included into our calculations. Also, it must be stated that the present meta-analyses serve as a basis to estimate the impact of the differences that appear with age, between sexes and in correlation to cognitive abilities, and that we don't analyze inter-individual differences per se. Finally, these meta-analyses focused only on PSG and sleep spindle properties, not on the relationship of sleep spindles to slow oscillations or hippocampal sharp-wave ripples. For instance, a recent EEG and MRI combined study revealed that reasoning abilities are linked to sleep spindles, specifically in terms of their coupling status. Specifically, coupled spindles, which involve the putamen and thalamus, showed a positive correlation with reasoning. In contrast, uncoupled spindles, associated with hippocampal activation, exhibited a negative correlation with reasoning, possibly due to reduced memory processing (Baena et al., 2023). The increasing ability to record from patients with intracranial electrodes during sleep will surely furthermore contribute valuable information to deciphering corresponding roles of sleep spindles.

### 4.5 Conclusions and future remarks

To our knowledge, this is the only comprehensive meta-analysis quantifying age- and sex-related differences in sleep spindles, as well as analyzing the correlation between sleep spindles and cognitive ability in a total of 1878 healthy subjects from 42 studies. Since baseline EEG does appear to show systematic differences dependent upon age, sex and cognitive ability, we suggest factoring them in when sleep developing or planning sleep manipulations studies. For instance, the reduced spindle power in males, in particular of older males may suggest that for transcranial electric stimulation on average higher current strengths should be used to obtain the same efficacy as in age-matched females. Considering the lower density, amplitude and duration of sleep spindles in older subjects, success of manipulations may benefit from increased effort into algorithms for spindle (or other oscillatory rhythms) detection. Necessity may be exacerbated by the poorer sleep efficiency in (healthy) older men. On the other hand, extending the conclusion about sex dependency beyond the influence of spindle generators to other brain rhythms is an important consideration. In addition to accounting for sensory thresholds when using acoustic stimulation to enhance sleep rhythms, more accurate estimates of stimulus-evoked brain responses from EEG scalp recordings may require individualized head models that reflect each person's unique conductivity properties. Beyond biology, increased open-source code sharing to standardize methods and enable cross-validation of spindle detection methods is relevant. Finally, because outcome measures like memory consolidation as well as the effects of NIBS depend on trait-like features, variability in sleep spindle properties may partly explain differences in NIBS efficacy. Moreover, our findings further suggest that inter-individual spindle properties—particularly spindle frequency—could be valuable parameters for optimizing NIBS approach.
