# Supplementary material for: Differences in sleep spindles and polysomnography in humans: a meta-analysis on the influence of age, sex, and cognitive ability
Source: Front Sleep. 2026 Apr 14;5:1802882. doi: 10.3389/frsle.2026.1802882 (PMC13120934; doi:10.3389/frsle.2026.1802882)
Supplement: Supplementary file 1 [file Table_1.docx]

**Supplementary Information**

1. **PRISMA checklist**

- Supplementary Table 1a, 1b.PRISMA 2020 and PRISMA-S checklists.

1. **Systematic Reference search**

- Supplementary Table 2. Systematic reference search regarding sleep electrophysiology and sleep spindles differences with aging in humans from August 2020.
- Supplementary Table 3. Systematic reference search regarding sleep electrophysiology and sleep spindles differences between sexes in humans from August 2020.
- Supplementary Table 4. Systematic reference search regarding sleep electrophysiology, sleep spindles and intelligence in humans from August 2020.
- Supplementary Table 5. Reasons for exclusion from the meta-analysis during full-text screening regarding sleep spindles and sleep electrophysiological differences with aging in humans.
- Supplementary Table 6. Reasons for exclusion from the meta-analysis during full-text screening regarding sleep spindles and sleep electrophysiological differences between sexes in humans.
- Supplementary Table 7. Reasons for exclusion from the meta-analysis during full-text screening regarding sleep spindles and cognitive ability in humans.

1. **Individual bias assessment**

- Supplementary Table 8. Quality scores of the individual studies obtained using the Mixed Methods Appraisal Tool (MMAT).

1. **Age-related differences in sleep spindles and sleep electrophysiology**

- Supplementary Table 9. Main characteristics of the studies included in the meta-analysis about the age-related differences in sleep spindles and sleep electrophysiology.
- Supplementary Table 10. Sleep spindles parameters published in each study investigating the age-related differences in humans.
- Supplementary Table 11. Sleep electrophysiology parameters published in each study investigating the age-related differences in humans.
- Supplementary Table 12. Meta-analytic results of the age-related differences in the sleep spindles.
  - Supplementary Figure 1. Publication bias in the age-related changes in sleep spindles.
  - Supplementary Figure 2. Publication bias in the age-related changes in fast and slow sleep spindles.
  - Supplementary Table 13. Meta-analytic results of the age-related differences in the sleep EGG.
  - Supplementary Figure 3. Publication bias in the age-related changes in the sleep EEG.
  - Supplementary Table 14. Sensitivity analyses: meta-analytic results of the age-related differences in the sleep spindles without Guazzelli et al. (1986) and without Principe & Smith (1982).
  - Supplementary Table 15. Sensitivity analyses: meta-analytic results of the age-related differences in the sleep EEG without the nap study (Fogel et al., 2017).
  - Supplementary Figure 4. Meta-regression analyses: age-related differences in sleep spindles and sleep EEG moderated by the percentage of females.
- Supplementary Table 16. Meta-regression analyses: differences in sleep spindles and sleep EEG with age moderated by the percentage of females.

1. **Differences in the sleep spindles and sleep electrophysiology between sexes**
   - Supplementary Table 17. Main characteristics of the studies included in the meta-analysis about the differences in sleep spindles and sleep electrophysiology between sexes.
   - Supplementary Table 18. Sleep spindles parameters published in each study investigating the differences between sexes in humans.
   - Supplementary Table 19. Sleep electrophysiology parameters published in each study investigating the differences between sexes in humans.
   - Supplementary Table 20. Meta-analytic results of the differences in sleep spindles between sexes.
   - Supplementary Table 21. Sensitivity analysis: meta-analytic results of the differences in the sleep spindles density between sexes without Gaillard and Blois, 1981.
   - Supplementary Table 22. Meta-analytic results of the differences in the sleep EEG between sexes.
   - Supplementary Table 23. Meta-analytic results of the differences in the sleep spindles between sexes in young, middle-aged, and older subjects.
   - Supplementary Figure 5. Meta-regression analyses: differences in sleep spindles between sexes moderated by age.
   - Supplementary Table 24. Meta-regression analyses: differences in the sleep spindles between sexes moderated by age.
2. **Sleep Spindles and cognitive ability**
   - Supplementary Table 25. Main characteristics of the studies included in the meta-analysis about sleep spindles and cognitive ability in children.
   - Supplementary Table 26. Sleep spindles parameters published in each study investigating their relation to cognitive ability in children.
   - Supplementary Table 27. Main characteristics of the studies included in the meta-analysis about sleep spindles and cognitive ability in adolescents.
   - Supplementary Table 28. Sleep spindles parameters published in each study investigating their relation to cognitive ability in adolescents.
   - Supplementary Table 29. Main characteristics of the studies included in the meta-analysis about sleep spindles and cognitive ability in adults.
   - Supplementary Table 30. Sleep spindles parameters published in each study investigating their relation to cognitive ability in adults.
   - Supplementary Table 31. Main characteristics of the studies included in the meta-analysis about sleep spindles and cognitive ability in elderly.
   - Supplementary Table 32. Sleep spindles parameters published in each study investigating their relation to cognitive ability in elderly.

- Supplementary Table 33. Meta-analytic results of the correlation between sleep spindles and cognitive ability in all ages.
- Supplementary Table 34. Meta-analytic results of the correlation between sleep spindles and cognitive ability in children.
- Supplementary Table 35. Meta-analytic results of the correlation between sleep spindles and cognitive ability in adolescents.
- Supplementary Table 36. Meta-analytic results of the correlation between sleep spindles and cognitive ability in adults.
- Supplementary Table 37. Meta-analytic results of the correlation between sleep spindles and cognitive ability in older adults.
- Supplementary Figure 6. Publication bias from the correlation between sleep spindles and cognitive ability.
  - Supplementary Table 38. Sensitivity analyses: Meta-analytic results of the correlation between sleep spindles and cognitive ability without the approximated Pearson correlations.
  - Supplementary Table 39. Sensitivity analyses: Meta-analytic results of the correlation between fast and slow sleep spindles and cognitive ability without the nap study (Ujma et al., 2015) and without the approximated Pearson correlations.
  - Supplementary Figure 7. Meta-regression analyses: Correlation between sleep spindles and cognitive ability moderated by the percentage of females.
  - Supplementary Table 40. Meta-regression analyses: Correlation between sleep spindles and cognitive ability moderated by the percentage of females.

**1. PRISMA checklist**

**Supplementary Table 1a. PRISMA 2020 checklist including the abstract checklist (Modifyed from: Page et al., 2021).** NA: not applicable.

| **Section/topic** | **#** | **Checklist item** | **Section** |
| --- | --- | --- | --- |
| **TITLE** | | | |
| Title | 1 | Identify the report as a systematic review, meta-analysis, or both. | Title Pg. |
| **ABSTRACT** | | | |
| Abstract Title | 2a | Identify the report as a systematic review. | NA |
| Background: Objectives | 2b | Provide an explicit statement of the main objective(s) or question(s) the review addresses. | Abstract |
| Methods: Eligibility criteria | 2c | Specify the inclusion and exclusion criteria for the review. | Abstract |
| Methods: Information sources | 2d | Specify the information sources (e.g. databases, registers) used to identify studies and the date when each was last searched. | Abstract |
| Methods: Risk of bias | 2e | Specify the methods used to assess risk of bias in the included studies. | Abstract |
| Methods: Synthesis of results | 2f | Specify the methods used to present and synthesise results. | Abstract |
| Results: Included studies | 2g | Give the total number of included studies and participants and summarise relevant characteristics of studies. | Abstract |
| Results: Synthesis of results | 2h | Present results for main outcomes, preferably indicating the number of included studies and participants for each. If meta-analysis was done, report the summary estimate and confidence/credible interval. If comparing groups, indicate the direction of the effect (i.e. which group is favoured). | Abstract |
| Discussion: Limitations of evidence | 2i | Provide a brief summary of the limitations of the evidence included in the review (e.g., study risk of bias, inconsistency and imprecision). | Abstract |
| Discussion: Interpretation | 2j | Provide a general interpretation of the results and important implications. | Abstract |
| Other: Funding | 2k | Specify the primary source of funding for the review. | Not included here |
| Other: Registration | 2l | Provide the register name and registration number. | NA |
| **INTRODUCTION** | | | |
| Rationale | 3 | Describe the rationale for the review in the context of existing knowledge. | 1.0 |
| Objectives | 4 | Provide an explicit statement of the objective(s) or question(s) the review addresses. | 1.0 |
| **METHODS**   | | | |
| Eligibility criteria | 5 | Specify the inclusion and exclusion criteria for the review and how studies were grouped for the syntheses. | 2.1 |
| Information sources | 6 | Specify all databases, registers, websites, organisations, reference lists and other sources searched or consulted to identify studies. Specify the date when each source was last searched or consulted. | 2.1 |
| Search strategy | 7 | Present the full search strategies for all databases, registers, and websites, including any filters and limits used. | 2.1 |
| Selection process | 8 | Specify the methods used to decide whether a study met the inclusion criteria of the review, including how many reviewers screened each record and each report retrieved, whether they worked independently, and if applicable, details of automation tools used in the process. | 2.2 |
| Data collection process | 9 | Specify the methods used to collect data from reports, including how many reviewers collected data from each report, whether they worked independently, any processes for obtaining or confirming data from study investigators, and if applicable, details of automation tools used in the process. | 2.2 |
| Data items | 10a | List and define all outcomes for which data were sought. Specify whether all results that were compatible with each outcome domain in each study were sought (e.g. for all measures, time points, analyses), and if not, the methods used to decide which results to collect. | 2.2 |
|  | 10b | List and define all other variables for which data were sought (e.g. participant and intervention characteristics, funding sources). Describe any assumptions made about any missing or unclear information. | 2.2 |
| Study risk of bias assessment | 11 | Specify the methods used to assess risk of bias in the included studies, including details of the tool(s) used, how many reviewers assessed each study and whether they worked independently, and if applicable, details of automation tools used in the process. | 2.1 |
| Effect measures | 12 | Specify for each outcome the effect measure(s) (e.g. risk ratio, mean difference) used in the synthesis or presentation of results. | 2.3 |
| Synthesis methods | 13a | Describe the processes used to decide which studies were eligible for each synthesis (e.g., tabulating the study intervention characteristics and comparing against the planned groups for each synthesis (item #5)). | 2.2 |
|  | 13b | Describe any methods required to prepare the data for presentation or synthesis, such as handling of missing summary statistics, or data conversions. | 2.2 |
|  | 13c | Describe any methods used to tabulate or visually display results of individual studies and syntheses. | 2.3 |
|  | 13d | Describe any methods used to synthesize results and provide a rationale for the choice(s). If meta-analysis was performed, describe the model(s), method(s) to identify the presence and extent of statistical heterogeneity, and software package(s) used. | 2.3 |
|  | 13e | Describe any methods used to explore possible causes of heterogeneity among study results (e.g., subgroup analysis, meta-regression). | 2.3 |
|  | 13f | Describe any sensitivity analyses conducted to assess robustness of the synthesized results. | 2.3 |
| Reporting bias assessment | 14 | Describe any methods used to assess risk of bias due to missing results in a synthesis (arising from reporting biases). | 2.3 |
| Certainty assessment | 15 | Describe any methods used to assess certainty (or confidence) in the body of evidence for an outcome. | 2.3 |
| **RESULTS**   | | | |
| Study selection | 16a | Describe the results of the search and selection process, from the number of records identified in the search to the number of studies included in the review, ideally using a flow diagram. | 3 |
|  | 16b | Cite studies that might appear to meet the inclusion criteria, but which were excluded, and explain why they were excluded. | 3 |
| Study characteristics | 17 | Cite each included study and present its characteristics. | 3 |
| Risk of bias in studies | 18 | Present assessments of risk of bias for each included study. | 3 |
| Results of individual studies | 19 | For all outcomes, present, for each study: (a) summary statistics for each group (where appropriate) and (b) an effect estimate and its precision (e.g. confidence/credible interval), ideally using structured tables or plots. | 3 |
| Results of syntheses | 20a | For each synthesis, briefly summarize the characteristics and risk of bias among contributing studies. | 3 |
|  | 20b | Present results of all statistical syntheses conducted. If meta-analysis was done, present for each the summary estimate and its precision (e.g. confidence/credible interval) and measures of statistical heterogeneity. If comparing groups, describe the direction of the effect. | 3 |
|  | 20c | Present results of all investigations of possible causes of heterogeneity among study results. | 3 |
|  | 20d | Present results of all sensitivity analyses conducted to assess the robustness of the synthesized results. | 3 |
| Reporting biases | 21 | Present assessments of risk of bias due to missing results (arising from reporting biases) for each synthesis assessed. | 3 |
| Certainty of evidence | 22 | Present assessments of certainty (or confidence) in the body of evidence for each outcome assessed. | 3 |
| **DISCUSSION**   | | | |
| Discussion | 23a | Provide a general interpretation of the results in the context of other evidence. | 4 |
|  | 23b | Discuss any limitations of the evidence included in the review. | 4.4 |
|  | 23c | Discuss any limitations of the review processes used. | 4.4 |
|  | 23d | Discuss implications of the results for practice, policy, and future research. | 4.6 |
| **OTHER INFORMATION** | | | |
| Registration and protocol | 24a | Provide registration information for the review, including register name and registration number, or state that the review was not registered. | 2 |
|  | 24b | Indicate where the review protocol can be accessed, or state that a protocol was not prepared. | NA |
|  | 24c | Describe and explain any amendments to information provided at registration or in the protocol. | NA |
| Support | 25 | Describe sources of financial or non-financial support for the review, and the role of the funders or sponsors in the review. | 6 |
| Competing interests | 26 | Declare any competing interests of review authors. | 6 |
| Availability of data, code and other materials | 27 | Report which of the following are publicly available and where they can be found: template data collection forms; data extracted from included studies; data used for all analyses; analytic code; any other materials used in the review. | NA |

**2. Systematic reference search**

**Supplementary Table 1b.** PRISMA-S: An Extension to the PRISMA Statement for Reporting Literature Searches in Systematic Reviews (Rethlefsen et al., 2021).

| **Section/topic** | **#** | **Checklist item** | **Page #** |
| --- | --- | --- | --- |
| **INFORMATION SOURCES AND METHODS** | | | |
| Database name | 1 | Name each individual database searched, stating the platform for each. | 5 |
| Multi-database searching | 2 | If databases were searched simultaneously on a single platform, state the name of the platform, listing all of the databases searched. | 5 |
| Study registries | 3 | List any study registries searched. | N/A |
| Online resources and browsing | 4 | Describe any online or print source purposefully searched or browsed (e.g., tables of contents, print conference proceedings, web sites), and how this was done. | 5 |
| Citation searching | 5 | Indicate whether cited references or citing references were examined, and describe any methods used for locating cited/citing references (e.g., browsing reference lists, using a citation index, setting up email alerts for references citing included studies). | 5-6 |
| Contacts | 6 | Indicate whether additional studies or data were sought by contacting authors, experts, manufacturers, or others. | 6 |
| Other methods | 7 | Describe any additional information sources or search methods used. | 6 |
| **SEARCH STRATEGIES** | | | |
| Full search strategies | 8 | Include the search strategies for each database and information source, copied and pasted exactly as run. | 5 |
| Limits and restrictions | 9 | Specify that no limits were used, or describe any limits or restrictions applied to a search (e.g., date or time period, language, study design) and provide justification for their use. | N/A |
| Search filters | 10 | Indicate whether published search filters were used (as originally designed or modified), and if so, cite the filter(s) used. | N/A |
| Prior work | 11 | Indicate when search strategies from other literature reviews were adapted or reused for a substantive part or all of the search, citing the previous review(s). | N/A |
| Updates | 12 | Report the methods used to update the search(es) (e.g., rerunning searches, email alerts). | 9 |
| Dates of searches | 13 | For each search strategy, provide the date when the last search occurred. | 9 |
| **PEER REVIEW** | | | |
| Peer review | 14 | Describe any search peer review process. | N/A |
| **MANAGING RECORDS** | | | |
| Total Records | 15 | Document the total number of records identified from each database and other information sources. | 5-7, SI Tab.2-3 |
| Deduplication | 16 | Describe the processes and any software used to deduplicate records from multiple database searches and other information sources. | 5-7, SI Tab. 2-3 |

**Supplementary Table 2. Systematic reference search regarding sleep electrophysiology and sleep spindles differences with aging in humans from August 2020.**

| **Keywords** | **Pubmed** | **Google Scholar** | **Biomed Central** | **Science**  **Direct** | **Wiley Online Library** | **Livivo** | **Cochrane Library** | **Total** |
| --- | --- | --- | --- | --- | --- | --- | --- | --- |
| **Sleep spindles AND age** | 351 | 10 | 0 | 12 | 3 | 31 | 4 | 411 |
| **Sleep spindles AND aging** | 118 | 0 | 0 | 12 | 3 | 31 | 4 | 168 |
| **Sleep spindles AND older** | 69 | 4 | 0 | 5 | 3 | 4 | 0 | 85 |
| **Sleep spindles AND old** | 92 | 0 | 0 | 5 | 3 | 9 | 0 | 109 |
| **Sleep spindles AND**  **age-related** | 53 | 2 | 0 | 2 | 0 | 9 | 0 | 66 |
| **Sleep spindles AND lifespan** | 14 | 1 | 0 | 0 | 0 | 1 | 0 | 16 |
| **Total** | 697 | 17 | 0 | 36 | 12 | 85 | 8 | **855** |

Number of studies found for the given key words using the corresponding search engine.

**Supplementary Table 3. Systematic reference search regarding sleep electrophysiology and sleep spindles differences between sexes in humans from August 2020.**

| **Keywords** | **Pubmed** | **Google Scholar** | **Biomed Central** | **Science**  **Direct** | **Wiley Online Library** | **Livivo** | **Cochrane Library** | **Total** |
| --- | --- | --- | --- | --- | --- | --- | --- | --- |
| **Sleep spindles AND sex** | 55 | 5 | 0 | 23 | 0 | 2 | 4 | 89 |
| **Sleep spindles AND male AND female** | 706 | 0 | 0 | 10 | 0 | 0 | 74 | 790 |
| **Sleep spindles AND women AND men** | 23 | 0 | 0 | 3 | 0 | 0 | 7 | 33 |
| **Sleep spindles AND gender** | 74 | 3 | 0 | 10 | 0 | 1 | 2 | 90 |
| **Sleep spindles AND sexual dimorphism** | 23 | 4 | 0 | 2 | 0 | 3 | 0 | 32 |
| **Sleep spindles AND sex dimorphism** | 23 | 0 | 0 | 1 | 0 | 0 | 0 | 24 |
| **Sleep spindles AND sexually dimorphic** | 5 | 0 | 0 | 0 | 0 | 0 | 0 | 5 |
| **Total** | 909 | 12 | 0 | 49 | 0 | 6 | 87 | **1063** |

Number of studies found for the given key words using the corresponding search engine.

**Supplementary Table 4. Systematic reference search regarding sleep electrophysiology, sleep spindles and cognitive ability** **in humans from August 2020.**

| **Keywords** | **Pubmed** | **Google Scholar** | | **Biomed Central** | | **Science Direct** | | **Wiley Online Library** | **Livivo** | **Cochrane Library** | **Total** |
| --- | --- | --- | --- | --- | --- | --- | --- | --- | --- | --- | --- |
| **Sleep Spindles AND Intelligence** | 55 | | 7 | | 0 | | 0 | 0 | 5 | 2 | 69 |
| **Sleep Spindles AND IQ** | 22 | | 1 | | 0 | | 1 | 0 | 1 | 6 | 31 |
| **Sleep Spindles AND mental ability** | 14 | | 0 | | 0 | | 0 | 0 | 0 | 1 | 13 |
| **Sleep Spindles AND cognitive ability** | 45 | | 2 | | 0 | | 0 | 2 | 2 | 4 | 55 |
| **Sleep Spindles AND cognitive performance** | 132 | | 9 | | 0 | | 0 | 1 | 4 | 29 | 175 |
| **Sleep Spindles AND intellectual ability** | 8 | | 3 | | 0 | | 0 | 0 | 1 | 0 | 12 |
| **Total** | 276 | | 22 | | 0 | | 1 | 3 | 13 | 42 | **357** |

Number of studies found for the given key words using the corresponding search engine.

**Supplementary Table 5. Reasons for exclusion from the meta-analysis during full-text screening regarding sleep spindles and sleep electrophysiological differences with aging in humans.**

| **Reference** | **Reasons for exclusion** | **Reference** | **Reasons for exclusion** |
| --- | --- | --- | --- |
| Bowersox et al., 1985 | Not desired data | Pace-Schott and Spencer, 2011 | It is a book chapter |
| Cajochen et al., 2006 | It is a review | Purcell et al., 2017 | Not desired data |
| Carrier et al., 2001 | Not desired data | Schwarz et al., 2017 | Not desired data |
| Chellapa et al., 2012 | 40h Multiple nap protocol | Scullin, 2018 | It is a review |
| Clawson et al., 2016 | It is a review | Scullin et al., 2019 | Not desired data |
| Crowley et al., 2004 | Not desired data | Scullin and Bliwise, 2015 | It is a review |
| De la Calzada, 2000 | It is a review | Scullin and Gao, 2019 | It is a review |
| De Gennaro and Ferrara, 2003 | It is a review | Seibt et al., 2016 | It is an editorial |
| Dijk and Duffy, 1999 | Not desired data | Sitnikova et al., 2019 | Not desired data |
| Ehlers and Kupfer, 1997 | Not desired data | Smagula et al., 2020 | Not desired data |
| Fogel et al., 2012 | It is a review | Sprecher et al., 2016 | Not desired data |
| Guadagni et al., 2021 | Not desired data | Taillard et al., 2019 | Not desired data |
| Huupponen et al., 2002 | Not desired data | Ujma et al., 2014 | Not desired data |
| Iotchev et al., 2019 | Performed in pet dogs | Ujma et al., 2019 | Not desired data |
| Knoblauch et al., 2005 | 40h Multiple nap protocol | Van Luijtelaar and Bikbaevb, 2007 | Not desired data |
| Kubicki et al., 1989 | Not desired data and it is in German | Vien et al., 2019 | Not desired data |
| Li et al., 2020 | Not desired data | Waiquier, 1993 | Not desired data |
| Luca et al., 2015 | Not desired data | Wei et al., 1999 | Not desired data |
| Mander et al., 2017 | It is a review | Wright and Gilmore, 1985 | Not desired data |
| Mander et al., 2017B | Not desired data | Zhong et al., 2019 | It is a review |
| Martin et al., 2013 | Not desired data |  |  |
| Muehlroth et al., 2019 | Not desired data |  |  |
| Muehlroth and Werkle-Bergner, 2019 | It is a review |  |  |
| Münch et al., 2005 | 40h Multiple nap protocol |  |  |
| Münch et al., 2010 | Not desired data |  |  |

**Supplementary Table 6. Reasons for exclusion from the meta-analysis during full-text screening regarding sleep spindles and sleep electrophysiological differences between sexes in humans.**

| Reference | Reasons for exclusion |
| --- | --- |
| Ackerman er al., 2015 | Not desired data |
| Baker et al., 2018 | It is a review |
| Bódizs et al., 2014 | Not desired data |
| Brown et al., 2020 | Only females |
| Carrier et al., 2001 | Not desired data |
| Dijk et al., 2009 | Not desired data |
| Empson and Purdie, 1999 | It is a review |
| Genzel et al., 2012 | Not desired data |
| Luca et al., 2015 | Not desired data |
| Purcell et al., 2017 | Not desired data |
| Swift et al., 2019 | It is performed in rats |
| Ujma et al., 2017 | Not desired data |
| Ujma et al., 2019 | Not desired data |

**Supplementary Table 7. Reasons for exclusion from the meta-analysis during full-text screening regarding sleep spindles and cognitive ability** **in humans.**

| **Reference** | **Reasons for exclusion** | **Reference** | **Reasons for exclusion** |
| --- | --- | --- | --- |
| Ackermann et al., 2015 | Intelligence not measured | Hennies et al., 2016 | Intelligence not measured |
| Astill et al., 2014 | Intelligence not measured | Jegou et al., 2019 | Intelligence not measured |
| Bang et al., 2014 | Intelligence not measured | Lafortune et al., 2014 | Intelligence not measured |
| Barakat et al., 2013 | Intelligence not measured | Lustenberger et al., 2015 | Intelligence not measured |
| Blaskovich et al., 2016 | Intelligence not measured | Muehlroth et al., 2019 | Intelligence not measured |
| Bódizs et al., 2008 | Intelligence not measured | Piantoni et al., 2013 | Not desired data, IQ not measured |
| Clawson, 2016 | It is a review | Reynolds et al., 2018 | It is a meta-analysis |
| Clemens et al., 2006 | Not desired data on sleep spindles | Schabus et al., 2008 | Not desired data on sleep spindles |
| Dickelmann, 2014 | It is a review | Studte et al., 2017 | It is a nap study |
| Doucette et al., 2015 | Intelligence not measured | Ujma et al., 2017 | Not desired data on sleep spindles |
| Fang et al., 2020 | Not desired data on sleep spindles | Ujma, 2018 | It is a meta-analysis |
| Fogel et al., 2010 | Performed in rats | Ujma et al., 2019 | Not desired data on sleep spindles |
| Fogel and Smith, 2011 | It is a review | van Schalkwijk et al., 2019 | Intelligence not measured |
| Goldstone et al., 2020 | Not desired data on sleep spindles | van Schalkwijk et al., 2020 | Intelligence not measured |
| Gorgoni et al., 2020 | It is a review | Vermeulen et al., 2018 | Intelligence not measured |
| Hahn et al., 2020 | Intelligence not measured | Zinke et al., 2018 | Intelligence not measured |

**3. Individual bias assessment**

**Supplementary Table 8. Quality scores of the individual studies obtained using the Mixed Methods Appraisal Tool (MMAT).** The score of Fang et al. (2017) was not calculated because it was published as an abstract and some of their outcome is published elsewhere (Ujma, 2018). A score of 5 corresponds to 100% quality criteria met.

| **Reference** | **Quality** | **Reference** | **Quality** | **Reference** | **Quality** | **Reference** | **Quality** |
| --- | --- | --- | --- | --- | --- | --- | --- |
| **Bodizs et al., 2005** | 5 | **Fogel et al., 2017** | 5 | **Landolt et al., 1996** | 5 | **Schabus et al., 2006** | 5 |
| **Bodizs et al., 2014** | 5 | **Gaillard and Blois, 1981** | 3 | **Lustenberger et al., 2012** | 5 | **Sulkamo et al., 2019** | 5 |
| **Chatburn et al., 2013** | 5 | **Gaudreault 2018** | 5 | **Mander et al., 2014** | 5 | **Tessier et al., 2015** | 5 |
| **Crowley et al., 2002** | 5 | **Geiger et al., 2011** | 5 | **Muehlroth et al., 2019** | 5 | **Tucker and Fishbein, 2009** | 5 |
| **della Monica et al., 2018** | 5 | **Gruber et al., 2013** | 5 | **Nader and Smith, 2015** | 5 | **Ujma et al., 2014** | 5 |
| **Ehlers and Kupfer, 1997** | 5 | **Guadagni et al., 2021** | 5 | **Nicolas et al., 2001** | 5 | **Ujma et al., 2015** | 5 |
| **Eggert et al., 2021** | 5 | **Guazzelli 1986** | 5 | **Pesonen et al., 2019** | 5 | **Ujma et al., 2016** | 5 |
| **Fang et al., 2019** | 5 | **Hahn et al., 2018** | 5 | **Peters et al., 2007** | 5 | **Ward et al., 2014** | 5 |
| **Fogel and Smith, 2006** | 5 | **Helfrich et al., 2018** | 5 | **Peters et al., 2008** | 5 |  |  |
| **Fogel et al., 2007** | 5 | **Hoedlmoser et al., 2014** | 5 | **Peters et al., 2014** | 5 |  |  |
|  |  | **Huupponen et al., 2002** | 5 | **Principe and Smith, 1982** | 2.5 |  |  |

**4. Age-related differences in sleep spindles and sleep electrophysiology**

Briefly, positive effect sizes shown in the Supplementary Tables 12-15 indicate that old subjects shown a larger standardized mean difference than young subjects while negative effect sizes indicate the opposite direction of effects for the analyzed parameter. Interpretation of the magnitude of effects follows “the rule of thumb”, namely effect sizes (Hedge’s g) around 0.20 are small, around 0.50 are medium and around or above 0.80 are large in magnitude (Hedges, 1981; Durlak, 2009).

**Supplementary Table 9. Main characteristics of the studies included in the meta-analysis about the age-related differences in sleep spindles and sleep electrophysiology.** Under subjects, the total sample, the sample number separated by age and sex and the age ranges/mean age for both young and old groups are provided. Under Sleep Scoring, it is mentioned each scoring method followed by the authors. Under sleep spindles, the method of spindles detection, the electrode used for each type of spindle, the sleep stage used, and the frequency ranges are shown. RK: Rechtschaffen and Kales (1968). AASM (American Academy of Sleep Medicine): Iber et al. (2007). DK: Dement and Kleitman (1957). FSS: Fast Sleep Spindles, SSS: Slow Sleep Spindles. C: Central, F: frontal, P: Parietal. S2: Sleep Stage 2, NREMS: Non-Rapid Eye Movement Sleep, TST: Total Sleep Time.

|  | Subjects | | | | | | Sleep | | Sleep Spindles | | | | | |
| --- | --- | --- | --- | --- | --- | --- | --- | --- | --- | --- | --- | --- | --- | --- |
| Reference | **N** | **Young** | **Old** | **Females** | **Males** | **Age** | **Scoring** | **Spindle detection** | | **Spindles** | **FSS** | **SSS** | **Stage** | **Frequencies** |
| Crowley et al., 2002 | 34 | 14 | 20 | 6/9 | 8/11 | 21.4±2.4  75.65±6.3 | RK | Self-developed software | | C3 |  |  | S2 | 11-16 Hz |
| della Monica et al., 2018 | 130 | 66 | 64 | 29/44 | 37/20 | 20-30  65-84 | RK | Spectral power | | C3-C4 |  |  | NREMS | 12.25-15 Hz |
| Eggert et al., 2021 | 60 | 30 | 30 | 0/60 | 60/60 | 25.6±2.4  69.1±5.5 | AASM | Spectral power | | C3 |  |  | S2 | 11-16 Hz |
| Fogel et al., 2017: Nap | 28 | 13 | 15 | 6/9 | 7/6 | 19-30  55-69 | AASM | Ray et al. (2015) | | Cz | Pz | Fz | NREMS | 11-14 Hz  14-17 Hz |
| Gaudreault 2018 | 61 | 30 | 31 | 14/18 | 16/13 | 20-30  50-70 | AASM | Martin et al. (2013) | | Frontal |  |  | S2 | 11-15 Hz |
| Guazzelli 1986 | 66 | 20 | 46 | 0/35 | 20/13 | 20-30  66-78 | DK | Visual | | C3 |  |  | NREMS | 11.5-14.5 Hz |
| Helfrich et al., 2018 | 52 | 20 | 32 | 12/22 | 8/10 | 20.4±2.0  73.7±5.3 | RK | Mölle et al. (2011) Staresina et al. (2015) | | Fz |  |  | NREMS | 12-16 Hz |
| Landolt et al., 1996 | 16 | 8 | 8 |  | 8/8 | 20-26  57-64 | RK | Spectral power | | C3 |  |  | NREMS | 12.25-15 Hz |
| Mander et al., 2014 | 30 | 16 | 14 | 8/11 | 8/3 | 20.5±2.1  71.9±6.7 | RK | Mander et al. (2011) | | Several |  |  | S2 | 11-13 Hz  13-14 Hz |
| Muehlroth et al., 2019 | 53 | 24 | 29 | 13/14 | 11/15 | 18-28  63-74 | AASM | Mölle et al. (2011); Klinzing et al. (2018) | |  | Cz | F3-F4 | NREMS | 9-12.5 Hz  12.5-16 Hz |
| Nicolas et al., 2001 | 12 | 6 | 6 | 3/3 | 3/3 | 20-29  60-69 | RK | Visual | | C3 |  |  | S2 | 12-14 Hz |
| Peters et al., 2008 | 28 | 14 | 14 | 7/7 | 7/7 | 17-24  62-79 | RK | Visual | | C3-C4 |  |  | S2 | 12-16 Hz |
| Peters et al., 2014 | 48 | 24 | 24 | 12/12 | 12/12 | 17-24  60-85 | RK | Visual | | C3-C4 |  |  | S2 | 12-16 Hz |
| Principe and Smith, 1982 | 8 | 4 | 4 |  |  | 25-34  67-79 |  | Microcomputer | |  |  |  | TST | 11.4-17.1 Hz |

**Supplementary Table 10. Sleep spindles parameters published in each study investigating the age-related differences in humans.** Dens: Density, Amp: Amplitude, Dur: Duration, Freq: Frequency, Co: Count, Sigma: Spindle power.

|  | Sleep Spindles | | | | | | Fast Sleep Spindles | | | | | | Slow Sleep Spindles | | | | | |  |
| --- | --- | --- | --- | --- | --- | --- | --- | --- | --- | --- | --- | --- | --- | --- | --- | --- | --- | --- | --- |
| Reference | **Dens** | **Amp** | **Dur** | **Freq** | **Co** | **Sigma** | **Dens** | **Amp** | **Dur** | **Freq** | **Co** | **Sigma** | **Dens** | **Amp** | **Dur** | **Freq** | **Co** | **Sigma** |  |
| Crowley et al., 2002 | X | X | X | X |  |  |  |  |  |  |  |  |  |  |  |  |  |  |  |
| della Monica et al., 2018 |  |  |  |  |  | X |  |  |  |  |  |  |  |  |  |  |  |  |  |
| Eggert et al., 2021 |  |  |  |  |  | X |  |  |  |  |  |  |  |  |  |  |  |  |  |
| Fogel et al., 2017 | X |  | X | X | X |  | X |  | X | X | X |  | X |  | X | X | X |  |  |
| Gaudreault 2018 | X | X |  |  |  | X |  |  |  |  |  |  |  |  |  |  |  |  |  |
| Guazzelli 1986 | X | X | X | X |  |  |  |  |  |  |  |  |  |  |  |  |  |  |  |
| Helfrich et al., 2018 |  | X | X | X | X | X |  |  |  |  |  |  |  |  |  |  |  |  |  |
| Landolt et al., 1996 |  |  |  |  |  |  |  |  |  |  |  | X |  |  |  |  |  | X |  |
| Mander et al., 2014 | X |  | X | X |  |  | X |  | X | X |  |  | X |  | X | X |  |  |  |
| Muehlroth et al., 2019 |  |  |  |  |  |  | X | X |  | X | X |  | X | X |  | X | X |  |  |
| Nicolas et al., 2001 | X |  | X | X | X |  |  |  |  |  |  |  |  |  |  |  |  |  |  |
| Peters et al., 2008 | X |  |  |  |  |  |  |  |  |  |  |  |  |  |  |  |  |  |  |
| Peters et al., 2014 | X |  |  |  |  |  |  |  |  |  |  |  |  |  |  |  |  |  |  |
| Principe and Smith, 1982 |  | X |  |  |  |  |  |  |  |  |  |  |  |  |  |  |  |  |  |

**Supplementary Table 11. Sleep electrophysiology parameters published in each study investigating the age-related differences in humans.** SLat: Sleep Latency, WASO: Wake After Sleep Onset, TST: Total Sleep Time, SEff: Sleep Efficiency, S1: Sleep Stage 1, S2: Sleep Stage 2, S3: Sleep Stage 3, S4: Sleep Stage 4, SWS: Slow Waves Sleep, REMS: Rapid-Eye Movement Sleep.

|  | **Sleep Quality** | | | | **Sleep Stages** | | | | | | |
| --- | --- | --- | --- | --- | --- | --- | --- | --- | --- | --- | --- |
| **Reference** | **SLat** | **WASO** | **TST** | **SEff** | **S1** | **S2** | **S3** | **S4** | **SWS** | **REMS** |  |
| **Crowley et al., 2002** | X |  | X | X | X | X |  |  | X | X |  |
| **della Monica et al., 2018** | X |  | X | X | X | X |  | X | X | X |  |
| Eggert et al., 2021 |  |  |  |  |  |  |  |  |  |  |  |
| **Fogel et al., 2017** | X | X | X | X | X | X |  |  | X | X |  |
| **Gaudreault 2018** | X |  | X | X | X | X |  |  | X | X |  |
| **Guazzelli 1986** |  |  |  |  |  |  |  |  |  |  |  |
| **Helfrich et al., 2018** | X | X | X | X | X | X |  |  | X | X |  |
| **Landolt et al., 1996** | X | X | X | X | X | X | X | X | X | X |  |
| **Mander et al., 2014** | X | X | X |  | X | X |  |  | X | X |  |
| **Muehlroth et al., 2019** | X |  | X | X |  |  |  |  |  |  |  |
| **Nicolas et al., 2001** |  |  | X | X | X | X | X | X | X | X |  |
| **Peters et al., 2008** |  | X | X | X | X | X |  |  | X | X |  |
| **Peters et al., 2014** |  | X | X | X | X | X |  |  | X | X |  |
| **Principe and Smith, 1982** |  |  |  |  |  |  |  |  |  |  |  |

**Supplementary Table 12. Meta-analytic results of the age-related differences in the sleep spindles.** k = number of studies. N = total sample. Effect size is denoted as Hedges’ g in both random- and fixed-effects models. Square brackets show the lower and upper limits from the confidence intervals (95%). p_g_ represents the p-vaue from the effect size. Heterogeneity is shown by the Q (Cochran’s Q and its p-value: p_Q_), I^2^, Tau and T^2^ statistics. Egger’s (p): Egger’s regression’s p-value. Begg (p): Begg and Mazumbar correlation’s p-value. Significances are presented in bold. Amp: amplitude, Dens: density, Dur: duration, Freq: frequency, Sigma: absolute spindle power, 13-14Hz: relative spindle power between 13-14Hz, 14-15 Hz: relative spindle power between 14-15Hz, 12-13 Hz: relative spindle power between 12-13Hz. NC: not sufficient data to calculate.

|  | | | **Random effects** | | **Fixed effects** | | **Heterogeneity** | | | |  | **Publication bias** | |  |
| --- | --- | --- | --- | --- | --- | --- | --- | --- | --- | --- | --- | --- | --- | --- |
| **Sleep Spindles** | **k** | **N** | **Effect size [95% CI]** | **p_g_** | **Effect size [95% CI]** | **p_g_** | **Q** | **p_Q_** | **I^2^** | **Tau** | **T^2^** | **Egger’s (p)** | **Begg (p)** | |
| **Amp** | 5 | 221 | -1.26 [-2.32, -0.20] | **0.001** | -1.21 [-1.63, -0.79] | **0.0000000000000009** | 14.74 | **0.005** | 72.87 | 0.57 | 0.33 | 0.945 | 1 | |
| **Dens** | 8 | 307 | -1.66 [-2.15, -1.18] | **0.0000000000000004** | -1.61 [-1.92, -1.29] | **0.000** | 15.68 | **0.028** | 55.37 | 0.43 | 0.18 | 0.445 | 0.216 | |
| **Dur** | 6 | 222 | -1.63 [-3.73, 0.46] | **0.045** | -1.41 [-1.84, -0.98] | **0.000** | 79.82 | **0.0000000000000009** | 93.74 | 1.63 | 2.65 | 0.258 | 0.573 | |
| **Freq** | 6 | 222 | 0.26 [-0.94, 1.46] | 0.580 | -0.05 [-0.42, 0.32] | 0.718 | 53.85 | **0.0000000002** | 90.71 | 1.13 | 1.27 | 0.199 | 0.188 | |
| **Count** | 3 | 92 | -1.11 [-5.14, 2.92] | 0.235 | -1.05 [-2.10, 0.00] | **0.00002** | 35.90 | **0.00000002** | 94.43 | 1.80 | 3.23 | 0.906 | 0.602 | |
| **Sigma** | 4 | 303 | -0.41 [-2.16, 1.35] | 0.461 | -0.55 [-0.94, -0.17] | **0.000005** | 42.93 | **0.000000003** | 93.01 | 0.91 | 0.84 | 0.461 | 1 | |
| **Fast sleep**  **spindles** | **k** | **N** | **Effect size [95% CI]** | **p_g_** | **Effect size [95% CI]** | **p_g_** | **Q** | **p_Q_** | **I^2^** | **Tau** | **T^2^** | **Egger’s (p)** | **Begg (p)** | |
| **Amp** | 1 | 53 |  |  | -0.45 [NC] | 0.100 |  |  |  |  |  |  |  | |
| **Dens** | 3 | 111 | -1.24 [-4.40, 1.92] | 0.092 | -1.22 [-2.14, -0.30] | **0.00000001** | 23.20 | **0.000009** | 91.38 | 1.21 | 1.47 | 0.055 | 0.117 | |
| **Dur** | 2 | 58 | -0.30 [-18.54, 17.95] | 0.837 | -0.22 [-3.89, 3.45] | 0.447 | 24.59 | **0.0000007** | 95.93 | 1.99 | 3.96 | NC | 0.317 | |
| **Freq** | 3 | 111 | 0.43 [-2.62, 3.47] | 0.547 | 0.54 [-0.32, 1.40] | **0.006** | 21.32 | **0.00002** | 90.62 | 1.10 | 1.21 | 0.727 | 0.117 | |
| **Count** | 2 | 81 | -0.70 [-18.18, 16.78] | 0.611 | -0.85 [-4.06, 2.35] | **0.001** | 29.36 | **0.00000006** | 96.59 | 1.91 | 3.66 | NC | 0.317 | |
| **13-14Hz** | 1 | 16 |  |  | 1.45 [NC] | **0.007** |  |  |  |  |  |  |  | |
| **14-15Hz** | 1 | 16 |  |  | 0.37 [NC] | 0.443 |  |  |  |  |  |  |  | |
| **Slow sleep spindles** | **k** | **N** | **Effect size [95% CI]** | **p_g_** | **Effect size [95% CI]** | **p_g_** | **Q** | **p_Q_** | **I^2^** | **Tau** | **T^2^** | **Egger’s (p)** | **Begg (p)** | |
| **Amp** | 1 | 53 |  |  | -0.58 [NC] | **0.037** |  |  |  |  |  |  |  | |
| **Dens** | 3 | 111 | -1.28 [1.93, -0.64] | **0.000** | -1.28 [-2.17, -0.40] | **0.0000000004** | 1.05 | 0.591 | 0.00 | 0.00 | 0.00 | 0.394 | 0.117 | |
| **Dur** | 2 | 58 | 0.03 [-23.89, 23.94] | 0.989 | -0.05 [-3.99, 3.88] | 0.865 | 36.84 | **0.000000001** | 97.29 | 2.63 | 6.89 | NC | 0.317 | |
| **Freq** | 3 | 111 | 0.58 [-3.29, 4.45] | 0.520 | 0.12 [-0.74, 0.98] | 0.550 | 27.87 | **0.0000009** | 92.82 | 1.31 | 1.71 | 0.088 | 0.117 | |
| **Count** | 2 | 81 | -0.17 [-11.42, 11.08] | 0.847 | -0.39 [-3.32, 2.54] | 0.092 | 13.70 | **0.0002** | 92.70 | 1.21 | 1.45 | NC | 0.317 | |
| **12-13Hz** | 1 | 16 |  |  | 1.64 [NC] | **0.003** |  |  |  |  |  |  |  | |

Note: All results are discussed using the random effects model (DerSimonian & Laird, 1986), but the supplementary tables also provide the fixed effects model, and the corresponding levels of significance (95% confidence interval). When only one study was available for a category (e.g., for slow sleep spindle frequency), the effect size of the study was calculated using the fixed-effects model and data are only reported in a supplementary table.

**Supplementary Figure 1. Publication bias in the age-related changes in sleep spindles.** The funnel plots represent the effect size (Y-axis), the standard deviation (SD, X-axis) for each sleep spindle parameter and the reference of the outlier points. The combined effect size is depicted at the base of the funnel.

**
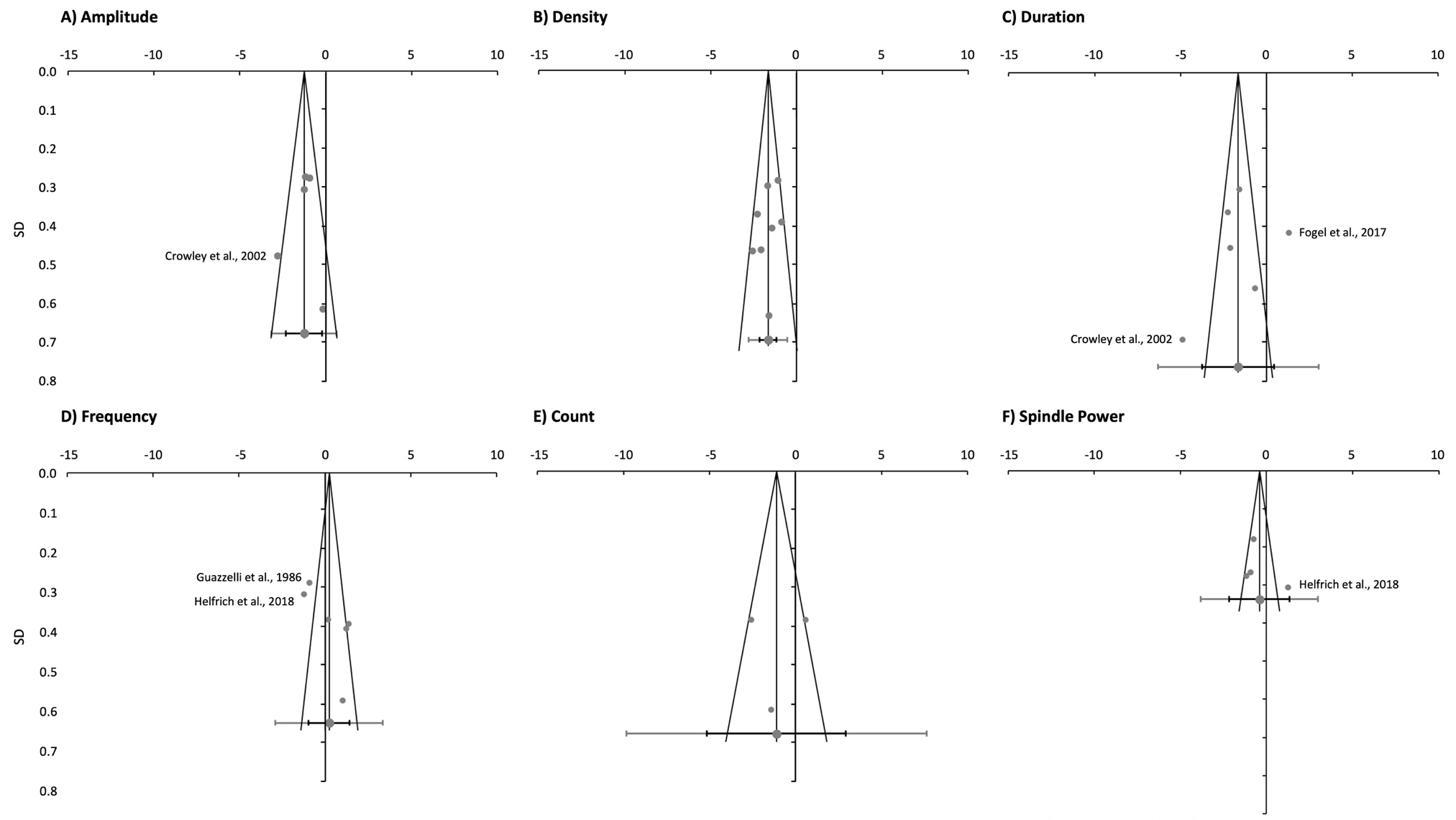
**

**Supplementary Figure 2. Publication bias in the age-related changes in fast and slow sleep spindles.** The funnel plots represent the effect size (Y-axis), the standard deviation (SD, X-axis) for each sleep spindle parameter. The combined effect size is depicted at the base of the funnel.

**
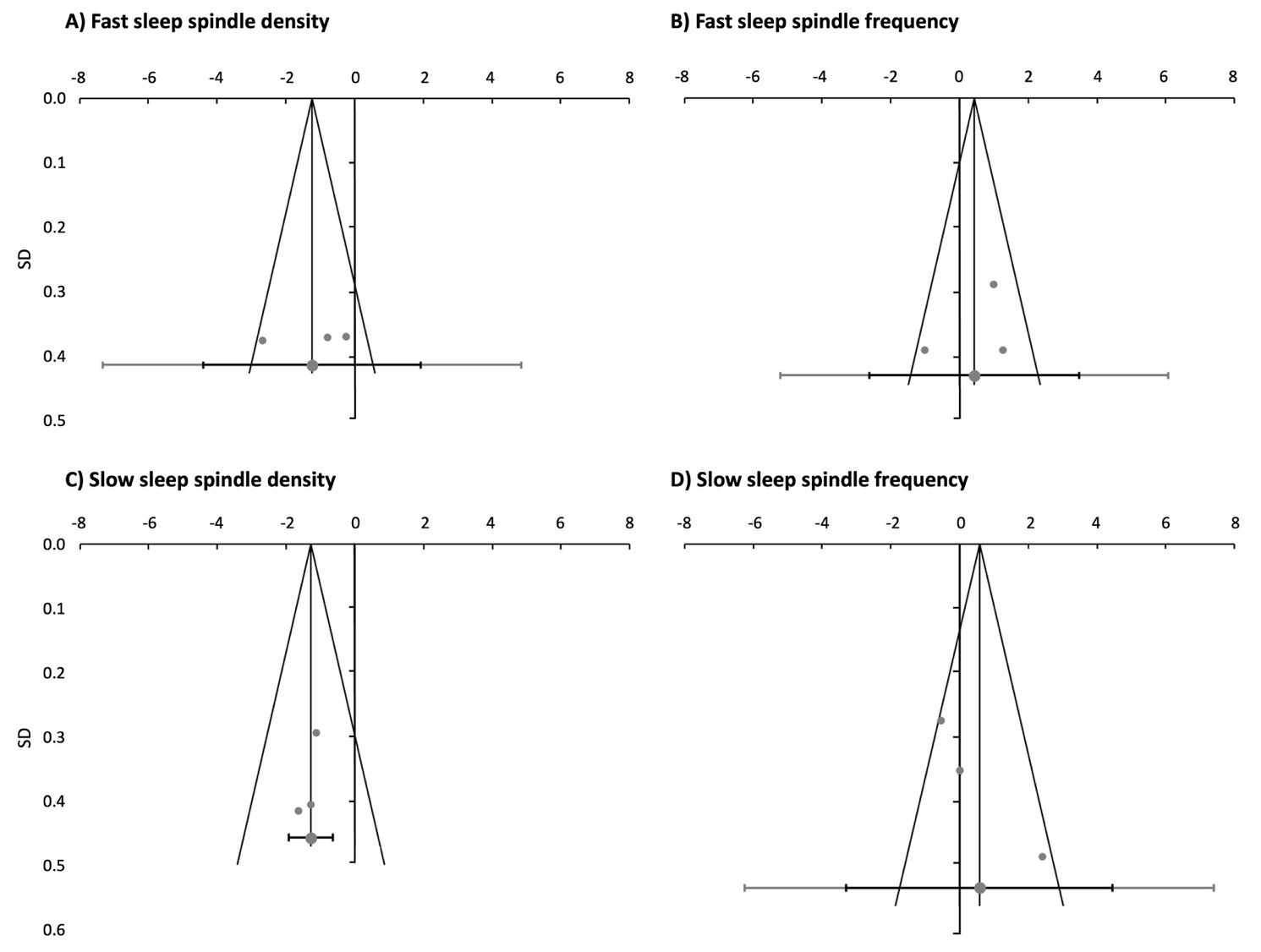
**

**Supplementary Table 13.** **Meta-analytic results of the age-related differences in the sleep EGG.** k = number of studies. N = total sample. Effect size is denoted as Hedges’ g in both random- and fixed-effects models. Square brackets show the lower and upper limits from the confidence intervals (95%). p_g_ represents the p-vaue from the effect size. Heterogeneity is shown by the Q (Cochran’s Q and its p-value: p_Q_), I^2^, Tau and T^2^ statistics. Egger’s (p): Egger’s regression’s p-value. Begg (p): Begg and Mazumbar correlation’s p-value. Significances are presented in bold. SLat: sleep latency, WASO: wake after sleep onset, TST: total sleep time, SEff: sleep efficiency, S1: sleep stage 1, S2: sleep stage 2, S3: sleep stage 3, S4: sleep stage 4, SWS: slow waves sleep, REMS: rapid eye movement sleep. NC: not sufficient data to calculate.

|  | | | **Random effects** | | **Fixed effects** | | **Heterogeneity** | | | | | **Publication bias** | |  |
| --- | --- | --- | --- | --- | --- | --- | --- | --- | --- | --- | --- | --- | --- | --- |
| **Sleep quality** | **k** | **N** | **Effect size [95% CI]** | **p_g_** | **Effect size [95% CI]** | **p_g_** | **Q** | **p_Q_** | **I^2^** | **Tau** | **T^2^** | **Egger’s (p)** | **Begg (p)** | |
| **SLat (min)** | 8 | 404 | 0.11 [-0.10, 0.32] | 0.230 | 0.11 [-0.13, 0.34] | 0.280 | 5.67 | 0.579 | 0.00 | 0.00 | 0.00 | 0.283 | 0.216 | |
| **WASO (min)** | 6 | 202 | 2.00 [-0.43, 4.43] | **0.035** | 1.38 [0.93, 1.83] | **0.000000000000004** | 91.45 | **0.000000000000000003** | 94.53 | 1.83 | 3.36 | 0.084 | 0.188 | |
| **TST (min)** | 11 | 492 | -0.86 [-1.53, -0.19] | **0.004** | -0.78 **[-0.99, -0.56]** | **0.0000000000000007** | 76.66 | **0.000000000002** | 86.96 | 0.85 | 0.72 | 0.347 | 0.312 | |
| **SEff (%)** | 10 | 462 | -1.07 [-1.93, -0.21] | **0.005** | -0.96 **[-1.19, -0.72]** | **0.000** | 97.66 | **0.00000000000000004** | 90.78 | 1.05 | 1.10 | 0.236 | 0.325 | |
| **Sleep Stages (min)** | **k** | **N** | **Effect size [95% CI]** | **p_g_** | **Effect size [95% CI]** | **p_g_** | **Q** | **p_Q_** | **I^2^** | **Tau** | **T^2^** | **Egger’s (p)** | **Begg (p)** | |
| **S1** | 10 | 439 | 0.99 [0.53, 1.45] | **0.000001** | 0.70 [0.48, 0.93] | **0.000000000001** | 39.45 | **0.000009** | 77.19 | 0.60 | 0.36 | **0.023** | 0.089 | |
| **S2** | 10 | 439 | 0.07 [-0.16, 0.30] | 0.485 | 0.07 [-0.14, 0.29] | 0.438 | 9.85 | 0.363 | 8.65 | 0.09 | 0.01 | 0.809 | 0.531 | |
| **S3** | 2 | 28 | -1.53 [-4.38, 1.32] | **0.000000000008** | -1.53 [-6.74, 3.68] | **0.0002** | 0.30 | 0.584 | 0.00 | 0.00 | 0.00 | NC | 0.317 | |
| **S4** | 3 | 158 | -2.39 [-5.18, 0.40] | **0.0002** | -1.51 [-2.30, -0.73] | **0.000** | 10.13 | **0.006** | 80.26 | 1.16 | 1.35 | 0.164 | 0.602 | |
| **SWS** | 10 | 439 | -1.70 [-2.54, -0.86] | **0.000004** | -1.36 [-1.60, -1.11] | **0.000** | 66.88 | **0.00000000006** | 86.54 | 0.92 | 0.84 | 0.062 | 0.089 | |
| **REMS** | 10 | 439 | -0.80 [-1.30, -0.29] | **0.0003** | -0.70 [-0.92, -0.47] | **0.000000000002** | 34.81 | **0.00006** | 74.14 | 0.55 | 0.30 | 0.607 | 0.421 | |

**Supplementary Figure 3. Publication bias in the age-related changes in the sleep EEG.** The funnel plots represent the effect size (Y-axis), the standard deviation (SD, X-axis) for each sleep EEG parameter and the reference of the outlier points. WASO: wake after sleep onset, TST: total sleep time, SWS: slow waves sleep, REMS: rapid eye movement sleep. The combined effect size is depicted at the base of the funnel.

**
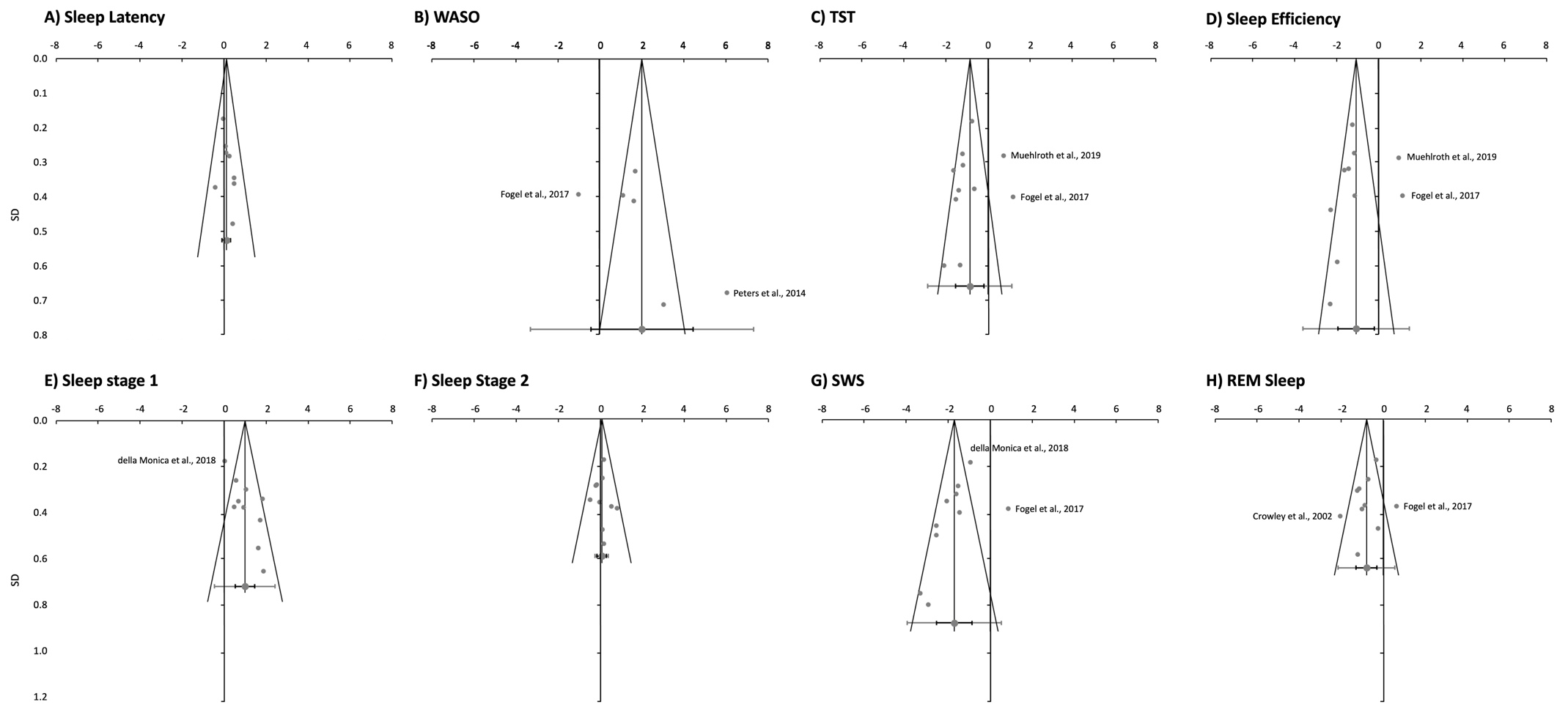
**

**Supplementary Table 14.** **Sensitivity analyses:** **meta-analytic results of the age-related differences in the sleep spindles without Guazzelli et al. (1986) and without Principe and Smith (1982).** k = number of studies. N = total sample. Effect size is denoted as Hedges’ g in both random- and fixed-effects models. Square brackets show the lower and upper limits from the confidence intervals (95%). p_g_ represents the p-value from the effect size. Heterogeneity is shown by the Q (Cochran’s Q and its p-value: p_Q_), I^2^, Tau and T^2^ statistics. Egger’s (p): Egger’s regression’s p-value. Begg (p): Begg and Mazumbar correlation’s p-value. Significances are presented in bold. Amp: amplitude, Dens: density, Dur: duration, Freq: frequency.

| **Without Guazzelli et al., 1986** | | | **Random effects** | | | **Fixed effects** | | **Heterogeneity** | | | | | **Publication bias** | |
| --- | --- | --- | --- | --- | --- | --- | --- | --- | --- | --- | --- | --- | --- | --- |
| **Sleep Spindles** | **k** | **N** | **Effect size [95% CI]** | | **p_g_** | **Effect size [95% CI]** | **p_g_** | **Q** | **p_Q_** | **I^2^** | **Tau** | **T^2^** | **Egger’s (p)** | **Begg (p)** |
| **Amp** | 4 | 155 | -1.36 [-2.96, 0.24] | | **0.007** | -1.33 [-1.90, -0.76] | **0.0000000000001** | 13.27 | **0.004** | 77.40 | 0.70 | 0.49 | 0.816 | 1 |
| **Dens** | 7 | 241 | -1.77 [-2.29, -1.25] | | **0.000** | -1.76 [-2.13, -1.39] | **0.000** | 11.34 | 0.078 | 47.10 | 0.38 | 0.15 | 0.783 | 0.453 |
| **Dur** | 5 | 156 | -1.67 [-4.46, 1.12] | | 0.097 | -1.34 [-1.90, -0.78] | **0.00000000003** | 79.45 | **0.0000000000000002** | 94.97 | 2.01 | 4.03 | 0.256 | 0.624 |
| **Freq** | 5 | 156 | 0.51 [-0.85, 1.87] | | 0.298 | 0.27[-0.20, 0.75] | 0.107 | 40.39 | **0.00000004** | 90.10 | 1.16 | 1.36 | 0.397 | 0.327 |
| **Without Principe and Smith, 1982** | | | **Random effects** | **Fixed effects** | | | | **Heterogeneity** | | | | |  |  |
| **Sleep Spindles** | **k** | **N** | **Effect size [95% CI]** | | **p_g_** | **Effect size [95% CI]** | **p_g_** | **Q** | **p_Q_** | **I^2^** | **Tau** | **T^2^** | **Egger’s (p)** | **Begg (p)** |
| **Amp** | 4 | 213 | -1.43 [-2.65, -0.20] | | **0.0002** | -1.28 **[-1.77, -0.78]** | **0.0000000000000002** | 11.55 | **0.009** | 74.02 | **0.54** | **0.29** | **0.010** | 0.174 |

**Supplementary Table 15.** **Sensitivity analyses:** **meta-analytic results of the age-related differences in the sleep EEG without the nap study (Fogel et al., 2017).** k = number of studies. N = total sample. Effect size is denoted as Hedges’ g in both random- and fixed-effects models. Square brackets show the lower and upper limits from the confidence intervals (95%). p_g_ represents the p-vaue from the effect size. Heterogeneity is shown by the Q (Cochran’s Q and its p-value: p_Q_), I^2^, Tau and T^2^ statistics. Egger’s (p): Egger’s regression’s p-value. Begg (p): Begg and Mazumbar correlation’s p-value. Significances are presented in bold. Amp: amplitude, Dens: density, Dur: duration, Freq: frequency . SLat: sleep latency, WASO: wake after sleep onset, TST: total sleep time, SEff: sleep efficiency. S1: sleep stage 1, S2: sleep stage 2, S3: sleep stage 3, S4: sleep stage 4, SWS: slow waves sleep, REMS: rapid eye movement sleep. NC: not sufficient data to calculate.

|  | | | **Random effects** | | | **Fixed effects** | | **Heterogeneity** | | | | | **Publication bias** | | |  |
| --- | --- | --- | --- | --- | --- | --- | --- | --- | --- | --- | --- | --- | --- | --- | --- | --- |
| **Sleep Spindles** | **k** | **N** | **Effect size [95% CI]** | | **p_g_** | **Effect size [95% CI]** | **p_g_** | **Q** | **p_Q_** | **I^2^** | **Tau** | **T^2^** | | **Egger’s (p)** | **Begg (p)** | |
| **Dens** | 7 | 279 | -1.77 [-2.25, -1.29] | | **0.000** | -1.71 **[-2.05, -1.36]** | **0.000** | 11.65 | 0.070 | 48.53 | 0.37 | 0.14 | | 0.397 | 0.293 | |
| **Dur** | 5 | 194 | -2.19 [-4.01, -0.37] | | **0.001** | -1.96 **[-2.47, -1.45]** | **0.000** | 26.22 | **0.00003** | 84.75 | 1.01 | 1.01 | | 0.295 | 0.624 | |
| **Freq** | 5 | 194 | 0.28 [-1.31, 1.86] | | 0.627 | -0.10 **[-0.54, 0.34]** | 0.531 | 53.29 | **0.00000000007** | 92.49 | 1.27 | 1.60 | | 0.278 | 0.624 | |
| **Count** | 2 | 64 | -2.08 [-9.46, 5.31] | | **0.000** | -2.24**[-6.30, 1.83]** | **0.000000000003** | 2.73 | 0.098 | 63.42 | 0.66 | 0.44 | | NC | 0.317 | |
| **Fast Sleep Spindles** | **k** | **N** | **Effect size [95% CI]** | | **p_g_** | **Effect size [95% CI]** | **p_g_** | **Q** | **p_Q_** | **I^2^** | **Tau** | **T^2^** | | **Egger’s (p)** | **Begg (p)** | |
| **Dens** | 2 | 83 | -1.73 [-13.7, 10.25] | | 0.067 | -1.72 [-5.07, 1.64] | **0.00000000008** | 12.77 | **0.0003** | 92.17 | 1.28 | 1.64 | | NC | 0.317 | |
| **Dur** | 1 | 30 |  |  |  | -1.73 [NC] | **0.00004** |  |  |  |  |  | |  |  | |
| **Freq** | 2 | 83 | 1.09 [-0.4, 2.64] | | **0.000** | 1.09 [-1.86, 4.04] | **0.000003** | 0.28 | 0.598 | 0.00 | 0.00 | 0.00 | | NC | 0.317 | |
| **Count** | 1 | 53 |  |  |  | -2.07 [NC] | **0,0000000009** |  |  |  |  |  | |  |  | |
| **Slow Sleep Spindles** | **k** | **N** | **Effect size [95% CI]** | | **p_g_** | **Effect size [95% CI]** | **p_g_** | **Q** | **p_Q_** | **I^2^** | **Tau** | **T^2^** | | **Egger’s (p)** | **Begg (p)** | |
| **Dens** | 2 | 83 | -1.29 [-4.42, 1.83] | | **0.0000001** | -1.29 [-4.33, 1.75] | **0.00000007** | 1.05 | 0.306 | 4.56 | 0.08 | 0.01 | | NC | 0.317 | |
| **Dur** | 1 | 30 |  |  |  | -1.85 [NC] | **0.00001** |  |  |  |  |  | |  |  | |
| **Freq** | 2 | 83 | -0.83 [-3.71, 3.09] | | 0.235 | -0.34 [-3.12, 2.44] | 0.121 | 1.45 | 0.228 | 31.14 | 0.21 | 0.05 | | NC | 0.317 | |
| **Count** | 1 | 53 |  |  |  | -1.04 [NC] | **0.0003** |  |  |  |  |  | |  |  | |
| **Sleep EEG** | **k** | **N** | **Effect size [95% CI]** | | **p_g_** | **Effect size [95% CI]** | **p_g_** | **Q** | **p_Q_** | **I^2^** | **Tau** | **T^2^** | | **Egger’s (p)** | **Begg (p)** | |
| **SLat (min)** | 7 | 376 | 0.15 [-0.04, 0.34] | | 0.058 | 0.15 [-0.10, 0.40] | 0.151 | 3.46 | 0.750 | 0.00 | 0.00 | 0.00 | | **0.006** | **0.024** | |
| **WASO (min)** | 5 | 174 | 2.59 [0.15, 5.03] | | **0.003** | 1.98 [1.44, 2.53] | **0.000** | 44.62 | **0.000000005** | 91.04 | 1.45 | 2.12 | | 0.117 | 0.142 | |
| **TST (min)** | 10 | 464 | -1.05 [-1.61, -0.49] | | **0.00002** | -0.90 [-1.12, -0.67] | **0.000** | 51.18 | **0.00000006** | 82.41 | 0.70 | 0.49 | | 0.166 | 0.128 | |
| **SEff (%)** | 9 | 434 | -1.30 [-2.06, -0.54] | | **0.00007** | -1.11 [-1.35, -0.86] | **0.000** | 68.24 | **0.00000000001** | 88.28 | 0.91 | 0.82 | | 0.147 | 0.144 | |
| **S1** | 9 | 411 | 1.05 [0.55, 1.56] | | **0.000002** | 0.72 [0.48, 0.96] | **0.000000000003** | 39.04 | **0.000005** | 79.51 | 0.64 | 0.41 | | **0.024** | 0.061 | |
| **S2** | 9 | 411 | 0.03 [-0.17, 0.22] | | 0.756 | 0.03[-0.20, 0.25] | 0.786 | 6.07 | 0.639 | 0.00 | 0.00 | 0.00 | | 0.709 | 1 | |
| **SWS** | 9 | 411 | -1.93 [-2.50, -1.36] | | **0.000000000000006** | -1.55 [-1.81, -1.29] | **0.000** | 30.78 | **0.0001** | 74.01 | **0.61** | **0.37** | | **0.002** | **0.022** | |
| **REMS** | 9 | 411 | -0.94 [-1.34, -0.53] | | **0.00000009** | -0.79[-1.03, -0.56] | **0.00000000000001** | 21.41 | **0.006** | 62.82 | 0.42 | 0.17 | | 0.344 | 0.297 | |

**Supplementary Figure 4. Meta-regression analyses: Age-related differences in sleep spindles and sleep EEG moderated by the percentage of females.** The bubble plots depict the effect sizes (Y-axis) against the percentage of females (X-axis) in each study (bubbles) overlaid with the predicted regression line. The size of the bubbles represents the study weight.


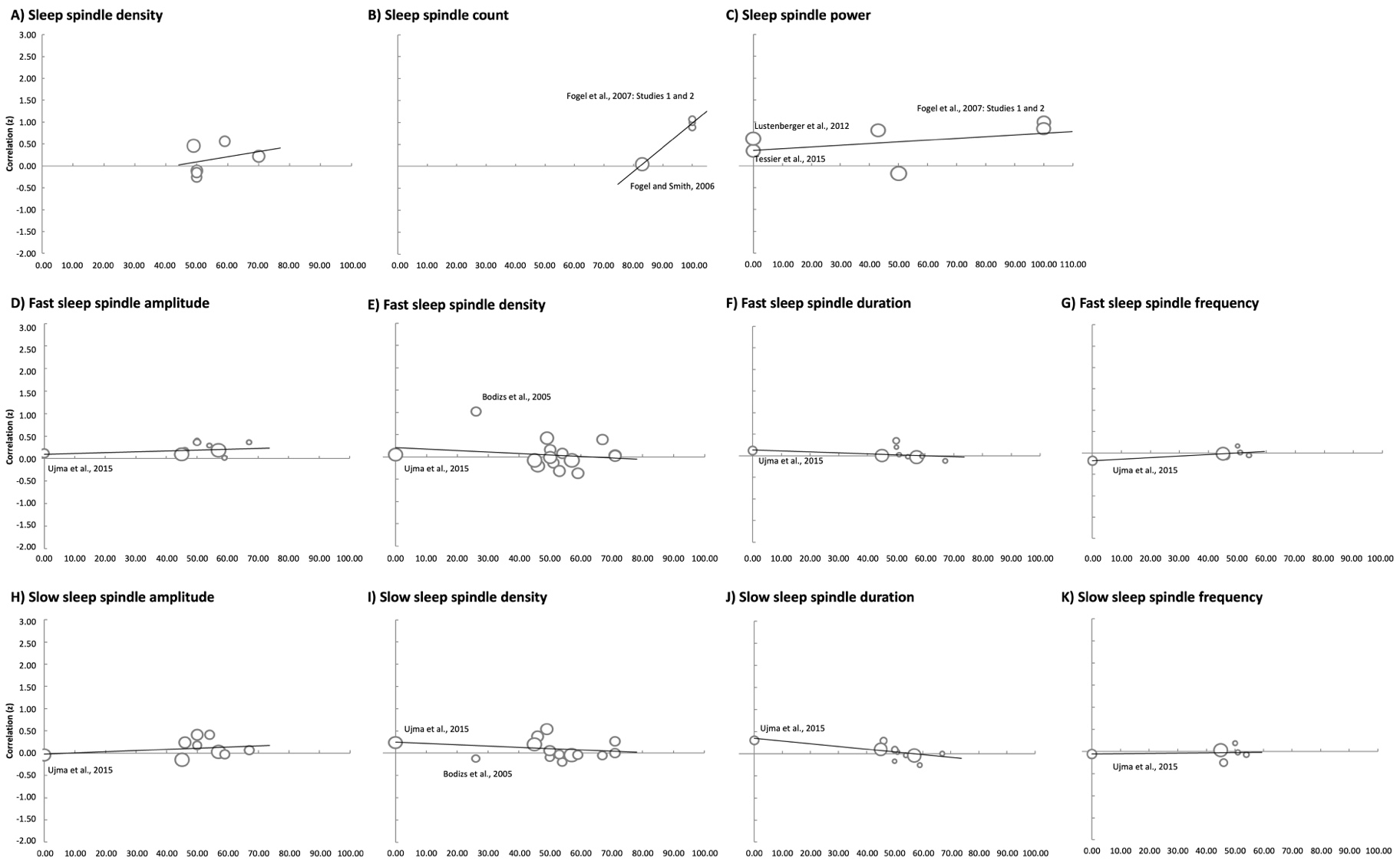


**Supplementary Table 16.** **Meta-regression analyses: Differences in sleep spindles and sleep EEG with age moderated by the percentage of females.**

| **Sleep Spindles** | **Slope** | **95% CI** | **p_z_** | **R^2^** |
| --- | --- | --- | --- | --- |
| **Amplitude** | 0.058 | [-0.09, 0.21] | 0.212 | 38.61 |
| **Density** | 0.067 | [-0.02, 0.16] | 0.076 | 35.67 |
| **Duration** | 0.050 | [-0.23, 0.33] | 0.639 | 3.94 |
| **Frequency** | -0.065 | [-0.24, 0.11] | 0.332 | 20.96 |
| **Count** | -0.126 | [-0.84, 0.59] | 0.445 | 36.89 |
| **Sigma (µV^2^)** | 0.022 | [0.02, -0.04] | 0.244 | 35.34 |
| **Fast sleep spindles** | **Slope** | **95% CI** | **p_z_** | **R^2^** |
| **Density** | 0.106 | [-0.64, 0.85] | 0.537 | 27.58 |
| **Frequency** | 0.072 | [-0.72, 0.86] | 0.692 | 13.57 |
| **Slow sleep spindles** | **Slope** | **95% CI** | **p_z_** | **R^2^** |
| **Density** | -0.043 | [-0.22, 0.14] | 0.306 | 99.85 |
| **Frequency** | -0.025 | [-1.10, 1.05] | 0.919 | 1.02 |

Slope and CI of the predicted regression line between sleep spindle parameter and combined sleep spindle parameters (amplitude, density, duration, frequency, count, sigma) moderated by the percentage of females in the study, corresponding R-square and p-value.

**5. Differences in sleep spindles and sleep electrophysiology between sexes**

Briefly, positive effect sizes shown in the Supplementary Tables 20-23 indicate that male subjects shown a larger standardized mean difference than female subjects while negative effect sizes indicate the opposite direction of effects for the analyzed parameter. Interpretation of the magnitude of effects follows “the rule of thumb”, namely effect sizes (Hedge’s g) around 0.20 are small, around 0.50 are medium and around or above 0.80 are large in magnitude (Hedges, 1981; Durlak, 2009).

**Supplementary Table 17. Main characteristics of the studies included in the meta-analysis about the differences in sleep spindles and sleep electrophysiology between sexes.** Under subjects, the total sample, the sample number separated by sex and the age ranges/mean age for both groups are provided. Under Sleep Scoring, it is mentioned each scoring method followed by the authors. Under sleep spindles, the method of spindles detection, the electrode used for each type of spindle, the sleep stage used, and the frequency ranges are shown. RK: Rechtschaffen and Kales (1968). AASM (American Academy of Sleep Medicine): Iber et al. (2007). IAM: Individual Adjusted Method (Bodizs et al., 2009). C: Central. S2: Sleep Stage 2, NREMS: Non-Rapid Eye Movement Sleep, TST: Total Sleep Time.

|  | Subjects | | | | Sleep | Sleep Spindles | | | |
| --- | --- | --- | --- | --- | --- | --- | --- | --- | --- |
| Reference | **N** | **Females** | **Males** | **Age** | **Scoring** | **Spindle detection** | **Electrodes** | **Stage** | **Frequencies** |
| Crowley et al., 2002: Young group | 14 | 6 | 8 | 18-25 | RK | Self-developed software | C3 | S2 | 11-16 Hz |
| Crowley et al., 2002: Old group | 20 | 9 | 11 | 75.5±6.3 | RK | Self-developed software | C3 | S2 | 11-16 Hz |
| della Monica et al., 2018: Young group | 66 | 29 | 37 | 20-30 | RK | Spectral power | C3-C4 | NREMS | 12.25-15 Hz |
| della Monica et al., 2018: Middle-aged group | 76 | 41 | 35 | 31-64 | RK | Spectral power | C3-C4 | NREMS | 12.25-15 Hz |
| della Monica et al., 2018: Old group | 64 | 44 | 20 | 65-84 | RK | Spectral power | C3-C4 | NREMS | 12.25-15 Hz |
| Ehlers and Kupfer, 1997: Study 1 | 32 | 14 | 18 | 20-29 | RK | Spectral Power | C4 | TST | 11-16 Hz |
| Ehlers and Kupfer, 1997: Study 2 | 29 | 14 | 15 | 30-40 | RK | Spectral Power | C4 | TST | 11-16 Hz |
| Eggert et al., 2021: old group | 60 | 30 | 30 | 68.5±5.6 | AASM | Spectral Power | C3 | S2 | 11-16 Hz |
| Gaillard and Blois 1981 | 10 | 5 | 5 | 33±11.5 | RK | Gaillard and Tissot (1973) | C4 | S2 | 11.6-17.2 Hz |
| Huupponen et al., 2002 | 40 | 20 | 20 | 22-49 | RK | Fuzzy Spindle detector | Several | S2 | 11-16 Hz |
| Pesonen et al., 2019 | 176 | 100 | 76 | 17 | AASM | IAM | C3 | S2 | 10-13 Hz  13-16 Hz |
| Ujma et al., 2014 | 160 | 72 | 88 | 17-69 | AASM | IAM | Several | NREMS | 11-13 Hz  13-15 Hz |

**Supplementary Table 18. Sleep spindles parameters published in each study investigating the differences between sexes in humans.** Dens: Density, Amp: Amplitude, Dur: Duration, Freq: Frequency, Sigma: Spindle power.

|  | Sleep Spindles | | | | | Fast Sleep Spindles | | | | Slow Sleep Spindles |
| --- | --- | --- | --- | --- | --- | --- | --- | --- | --- | --- |
| Reference | **Dens** | **Amp** | **Dur** | **Freq** | **Sigma** | **Dens** | **Amp** | **Dur** | **Freq** | **Freq** |
| Crowley et al., 2002: Young group | X | X | X | X |  |  |  |  |  |  |
| Crowley et al., 2002: Old group | X | X | X | X |  |  |  |  |  |  |
| della Monica et al, 2018: Young group |  |  |  |  | X |  |  |  |  |  |
| della Monica et al, 2018: Middle-aged group |  |  |  |  | X |  |  |  |  |  |
| della Monica et al, 2018: Old group |  |  |  |  | X |  |  |  |  |  |
| Ehlers and Kupfer, 1997: Study 1 |  |  |  |  | X |  |  |  |  |  |
| Ehlers and Kupfer, 1997: Study 2 |  |  |  |  | X |  |  |  |  |  |
| Eggert et al., 2021: Old group |  |  |  |  | X |  |  |  |  |  |
| Gaillard and Blois, 1981 | X |  |  |  |  |  |  |  |  |  |
| Huupponen et al., 2002 | X |  |  |  |  |  |  |  |  |  |
| Pesonen et al., 2019 | X | X | X |  |  |  |  |  |  |  |
| Ujma et al., 2014 |  |  |  |  |  | X | X | X | X | X |

**Supplementary Table 19. Sleep electrophysiology parameters published in each study investigating the differences between sexes in humans.** SLat: Sleep Latency, WASO: Wake After Sleep Onset, TST: Total Sleep Time, SEff: Sleep Efficiency, S1: Sleep Stage 1, S2: Sleep Stage 2, S3: Sleep Stage 3, S4: Sleep Stage 4, SWS: Slow Waves Sleep, REMS: Rapid-Eye Movement Sleep.

|  | Sleep Quality | | | | Sleep Stages | | | | | |
| --- | --- | --- | --- | --- | --- | --- | --- | --- | --- | --- |
| Reference | **SLat** | **WASO** | **TST** | **SEff** | **S1** | **S2** | **S3** | **S4** | **SWS** | **REMS** |
| Crowley et al., 2002: Young group |  |  |  |  |  |  |  |  |  |  |
| Crowley et al., 2002: Old group |  |  |  |  |  |  |  |  |  |  |
| della Monica et al, 2018: Young group | X |  | X | X | X | X |  | X | X | X |
| della Monica et al, 2018: Middle-aged group | X |  | X | X | X | X |  | X | X | X |
| della Monica et al, 2018: Old group | X |  | X | X | X | X |  | X | X | X |
| Ehlers and Kupfer, 1997: Study 1 | X | X | X | X | X | X | X | X | X | X |
| Ehlers and Kupfer, 1997: Study 2 | X | X | X | X | X | X | X | X | X | X |
| Eggert et al., 2021: Old group |  |  |  |  |  |  |  |  |  |  |
| Gaillard and Blois, 1981 |  |  |  |  |  |  |  |  |  |  |
| Huupponen et al., 2002 |  |  |  |  |  |  |  |  |  |  |
| Pesonen et al., 2019 |  | X | X | X | X | X |  |  | X | X |
| Ujma et al., 2014 |  |  |  |  |  |  |  |  |  |  |

**Supplementary Table 20. Meta-analytic results of the differences in sleep spindles between sexes.** k = number of studies. N = total sample. Effect size is denoted as Hedges’ g in both random- and fixed-effects models. Square brackets show the lower and upper limits from the confidence intervals (95%). p_g_ represents the p-vaue from the effect size. Heterogeneity is shown by the Q (Cochran’s Q and its p-value: p_Q_), I^2^, Tau and T^2^ statistics. Egger’s (p): Egger’s regression’s p-value. Begg (p): Begg and Mazumbar correlation’s p-value. Significances are presented in bold. Sigma density: mean power density of the spindle band (11-16 Hz). Sigma power: absolute power of the spindle band (12.25-15.00 Hz). NC: not sufficient data to calculate. Heterogeneity and Publication bias was not always calculated because most of the samples are from the same publication (Ehlers and Kupfer, 1997; Crowley et al., 2002; Della Monica et al., 2018).

|  | | | **Random effects** | | **Fixed effects** | | **Heterogeneity** | | | | | **Publication bias** | |
| --- | --- | --- | --- | --- | --- | --- | --- | --- | --- | --- | --- | --- | --- |
| **Sleep Spindles** | **k** | **N** | **Effect size [95% CI]** | **p_g_** | **Effect size [95% CI]** | **p_g_** | **Q** | **p_Q_** | **I^2^** | **Tau** | **T^2^** | **Egger’s (p)** | **Begg (p)** |
| **Amplitude** | 3 | 210 | -0.17 [-1.00, 0.67] | 0.391 | -0.08 [-0.68, 0.51] | 0.549 |  |  |  |  |  |  |  |
| **Density** | 5 | 260 | -0.21 [-0.99, 0.57] | 0.452 | -0.06 [-0.40, 0.29] | 0.651 | 9.03 | 0.060 | 55.70 | 0.39 | 0.15 | 0.078 | **0.050** |
| **Duration** | 3 | 210 | 0.22 [-0.74, 1.18] | 0.331 | 0.36 [-0.24, 0.96] | **0.010** |  |  |  |  |  |  |  |
| **Frequency** | 2 | 34 | -0.34 [-1.56, 0.87] | **0.0003** | -0.34 [-4.54, 3.86] | 0.298 |  |  |  |  |  |  |  |
| **Sigma density** | 2 | 61 | -0.35 [-3.72, 3.01] | 0.180 | -0.35 [-3.58, 2.87] | 0.162 |  |  |  |  |  |  |  |
| **Sigma power** | 4 | 266 | -0.59 [-1.16, -0.01] | **0.001** | -0.56 [-0.97, -0.16] | **0.00001** |  |  |  |  |  |  |  |
| **Fast sleep Spindles** | **k** | **N** | **Effect size [95% CI]** | **p_g_** | **Effect size [95% CI]** | **p_g_** | **Q** | **p_Q_** | **I^2^** | **Tau** | **T^2^** | **Egger’s (p)** | **Begg (p)** |
| **Amplitude** | 1 | 160 |  |  | -0.56 [NC] | **0.0005** |  |  |  |  |  |  |  |
| **Density** | 1 | 160 |  |  | 0.32 [NC] | **0.047** |  |  |  |  |  |  |  |
| **Duration** | 1 | 160 |  |  | 0.41 [NC] | **0.011** |  |  |  |  |  |  |  |
| **Frequency** | 1 | 160 |  |  | -0.65 [NC] | **0.00006** |  |  |  |  |  |  |  |
| **Slow sleep Spindles** | **k** | **N** | **Effect size [95% CI]** | **p_g_** | **Effect size [95% CI]** | **p_g_** | **Q** | **p_Q_** | **I^2^** | **Tau** | **T^2^** | **Egger’s (p)** | **Begg (p)** |
| **Frequency** | 1 | 160 |  |  | -0.45 [NC] | **0.005** |  |  |  |  |  |  |  |

**Supplementary Table 21. Sensitivity analysis: meta-analytic results of the differences in the sleep spindles density between sexes without Gaillard and Blois, 1981.** k = number of studies. N = total sample. Effect size is denoted as Hedges’ g in both random- and fixed-effects models. Square brackets show the lower and upper limits from the confidence intervals (95%). p_g_ represents the p-vaue from the effect size. Significances are presented in bold. NC: not calculated because more than two studies are needed.

|  | | | **Random effects** | | **Fixed effects** | |
| --- | --- | --- | --- | --- | --- | --- |
| **Sleep Spindles** | **k** | **N** | **Effect size [95% CI]** | **p_g_** | **Effect size [95% CI]** | **p_g_** |
| **Density** | 4 | 250 | -0.04 [-0.71, 0.64] | 0.864 | -0.01 [-0.41, 0.40] | 0.965 |

**Supplementary Table 22. Meta-analytic results of the differences in the sleep EEG between sexes.** k = number of studies. N = total sample. Effect size is denoted as Hedges’ g in both random- and fixed-effects models. Square brackets show the lower and upper limits from the confidence intervals (95%). p_g_ represents the p-vaue from the effect size. Heterogeneity is shown by the Q (Cochran’s Q and its p-value: p_Q_), I^2^, Tau and T^2^ statistics. Egger’s (p): Egger’s regression’s p-value. Begg (p): Begg and Mazumbar correlation’s p-value. Significances are presented in bold. SLat: sleep latency, WASO: wake after sleep onset, TST: total sleep time, SEff: sleep efficiency, S1: sleep stage 1, S2: sleep stage 2, S3: sleep stage 3, S4: sleep stage 4, SWS: slow waves sleep, REMS: Rapid eye movement sleep. Heterogeneity and publication bias were not calculated because some of the studies are samples from the same study (Ehlers and Kupfer, 1997; Crowley et al., 2002; Della Monica et al., 2018). NC: not sufficient data to calculate.

|  | | | **Random effects** |  | **Fixed effects** | |
| --- | --- | --- | --- | --- | --- | --- |
| **Sleep quality** | **k** | **N** | **Effect size [95% CI]** | **p_g_** | **Effect size [95% CI]** | **p_g_** |
| **SLat (min)** | 5 | 267 | 0.04 [-0.22, 0.30] | 0.678 | 0.04 [-0.30, 0.38] | 0.753 |
| **WASO (min)** | 3 | 237 | 0.23 [0.12, 0.34] | **0.000** | 0.23 [-0.33, 0.79] | 0.077 |
| **TST (min)** | 6 | 443 | -0.32 [-0.52, -011] | **0.00008** | -0.32 [-0.56, -0.07 | **0.001** |
| **SEff (%)** | 6 | 443 | -0.32 [-0.45, -0.19] | **0.00000000006** | -0.32 [-0.57, -0.07] | **0.001** |
| **Sleep stages (min)** | **k** | **N** | **Effect size** **[95% CI]** | **p_g_** | **Effect size [95% CI]** | **p_g_** |
| **S1** | 6 | 443 | 0.43 [-0.18, 1.04] | 0.068 | 0.35 [0.10, 0.60] | **0.0003** |
| **S2** | 6 | 443 | 0.11 [-0.68, 0.89] | 0.724 | -0.12 [-0.37, 0.13] | 0.230 |
| **S3** | 2 | 61 | -0.37 [-3.75, 3.22] | 0.334 | -0.26 [-3.47, 2.95] | 0.229 |
| **S4** | 5 | 267 | -0.56 [-1.25, 0.13] | **0.023** | -0.50 [-0.85, -0.15] | **0.00008** |
| **SWS** | 6 | 443 | -0.52 [-1.16, 0.13] | **0.039** | -0.34 [-0.59, -0.09] | **0.0004** |
| **REMS** | 6 | 443 | -0.17 [-0.45, 0.10] | 0.106 | -0.19 [-0.44, 0.05] | **0.044** |

**Supplementary** **Table 23. Meta-analytic results of the differences in the sleep spindles and PSG between sexes in young, middle-aged, and older subjects.** k = number of studies. N = total sample. Effect size is denoted as Hedges’ g in both random- and fixed-effects models. Square brackets show the lower and upper limits from the confidence intervals (95%). p_g_ represents the p-value from the effect size. Significances are presented in bold. Amp: amplitude, Dens: density, Dur: duration, Freq: frequency. Power mean density (µV^2^/octave) and power (µV^2^). SLat: sleep latency, WASO: wake after sleep onset, TST: total sleep time, SEff: sleep efficiency. S1: sleep stage 1, S2: sleep stage 2, S3: sleep stage 3, S4: sleep stage 4, SWS: slow waves sleep, REMS: Rapid eye movement sleep. Positive effect sizes represent greater values for males. *Random effects: g = -0.89 [-2.88, 1.10], p_g_ = 0.00000001.

| **Young subjects** | | | | | | |  | **Middle-aged subjects** | | | | | |  | **Older subjects** | | | |
| --- | --- | --- | --- | --- | --- | --- | --- | --- | --- | --- | --- | --- | --- | --- | --- | --- | --- | --- |
|  | | | **Random effects** | | **Fixed effects** | |  |  | | **Random effects** | | **Fixed effects** | |  |  | | **Fixed effects** | |
| **Sleep parameter** | **k** | **N** | **Effect size [95% CI]** | **p_g_** | **Effect size [95% CI]** | **p_g_** |  | **k** | **N** | **Effect size [95% CI]** | **p_g_** | **Effect size [95% CI]** | **p_g_** |  | **k** | **N** | **Effect size [95% CI]** | **p_g_** |
| **Amp** | 2 | 190 | -0.14  [-3.73, 3.45] | 0.628 | -0.04  [-1.88, 1.81] | 0.802 |  |  |  |  |  |  |  |  | 1 | 20 | -0.50 [NC] | 0.251 |
| **Dens** | 3 | 200 | -0.64  [-2.47, 1.20] | 0.135 | -0.13  [-0.75, 0.48] | 0.344 |  |  |  |  |  |  |  |  | 1 | 20 | -0.12 [NC] | 0.782 |
| **Dur** | 2 | 190 | 0.28  [-3.68, 4.25] | 0.362 | 0.41  [-1.45, 2.28] | **0.005** |  |  |  |  |  |  |  |  | 1 | 20 | -0.12 [NC] | 0.782 |
| **Freq** | 1 | 14 |  |  | -0.23 [NC] | 0.646 |  |  |  |  |  |  |  |  | 1 | 20 | -0.43 [NC] | 0.328 |
| **Sigma density** | 1 | 32 |  |  | -0.62 [NC] | 0.084 |  | 1 | 29 |  |  | -0.09 [NC] | 0.812 |  |  |  |  |  |
| **Sigma power** | 1 | 66 |  |  | -0.41 [NC] | 0.097 |  | 1 | 76 |  |  | -0.23 [NC] | 0.312 |  | 2 | 124 | -0.89 [-3.35, 1.56]* | **0.000004** |
| **SLat (min)** | 2 | 98 | -0.13  [-1.51, 1.26] | 0.247 | -0.13  [-2.68, 2.42] | 0.529 |  | 2 | 105 | 0.25  [-0.30, 0.79] | **0.00000001** | 0.25  [-2.21, 2.70] | 0.205 |  | 1 | 64 | -0.06 [NC] | 0.819 |
| **WASO (min)** | 2 | 208 | 0.22  [0.21, 0.22] | **0.000** | 0.22  [-1.55, 1.99] | 0.120 |  | 1 | 29 |  |  | 0.33 [NC] | 0.371 |  |  |  |  |  |
| **TST (min)** | 3 | 274 | -0.26  [-0.83, 0.30] | **0.045** | -0.28  [-0.80, 0.25] | **0.023** |  | 2 | 105 | -0.35  [-2.66, 1.95] | 0.051 | -0.35  [-2.83, 2.12] | 0.069 |  | 1 | 64 | -0.44 [NC] | 0.105 |
| **SEff (%)** | 3 | 274 | -0.24  [-0.26, -0.21] | **0.000** | -0.24  [-0.76, 0.28] | **0.050** |  | 2 | 105 | -0.47  [-0.90, -0.04] | **0.000** | -0.47  [-2.95, 2.01] | **0.016** |  | 1 | 64 | -0.44 [NC] | 0.105 |
| **S1** | 3 | 274 | 0.26  [-0.24, 0.76] | **0.025** | 0.26  [-0.24, 0.78] | **0.032** |  | 2 | 105 | 0.07  [-0.93, 1.06] | 0.388 | 0.07  [-2.38, 2.52] | 0.726 |  | 1 | 64 | 1.57 [NC] | **0.0000002** |
| **S2** | 3 | 274 | -0.40  [-0.93, 0.13] | **0.001** | -0.40  [-0.93, 0.13] | **0.001** |  | 2 | 105 | 0.82  [-9.96, 11.60] | 0.336 | 0.38  [-2.17, 2.94] | 0.056 |  | 1 | 64 | 0.36 [NC] | 0.184 |
| **S3** | 1 | 32 |  |  | 0 [NC] | 0.991 |  | 1 |  |  |  | -0.55 [NC] | 0.133 |  |  |  |  |  |
| **S4** | 2 | 208 | -0.08  [-1.25, 1.10] | 0.399 | -0.08  [-2.62, 2.47] | 0.697 |  | 2 | 105 | -0.65  [-5.15, 3.84] | 0.064 | -0.55  [-3.06, 1.96] | **0.006** |  | 1 | 64 | -1.27 [NC] | **0.00001** |
| **SWS** | 3 | 274 | -0.05  [-0.21, 0.10] | 0.150 | -0.05  [-0.57, 0.47] | 0.669 |  | 2 | 105 | -0.78  [-5.94, 4.37] | 0.053 | -0.64  [-3.18, 1.89] | **0.001** |  | 1 | 64 | -1.43 [NC] | **0.000001** |
| **REMS** | 3 | 274 | -0.04  [-0.95, 0.86] | 0.833 | -0.17  [-0.69, 0.36] | 0.170 |  | 2 | 105 | -0.29  [-0.80, 0.23] | **0.000000000002** | -0.29  [-2.75, 2.17] | 0.139 |  | 1 | 64 | -0.15 [NC] | 0.576 |

**Supplementary Figure 5. Meta-regression analyses: differences in sleep spindles between sexes moderated by age.** The bubble plots depict the effect size (Y-axis), the mean age in each study (X-axis) and the reference of the studies. The size of the bubbles represents the weight.

**
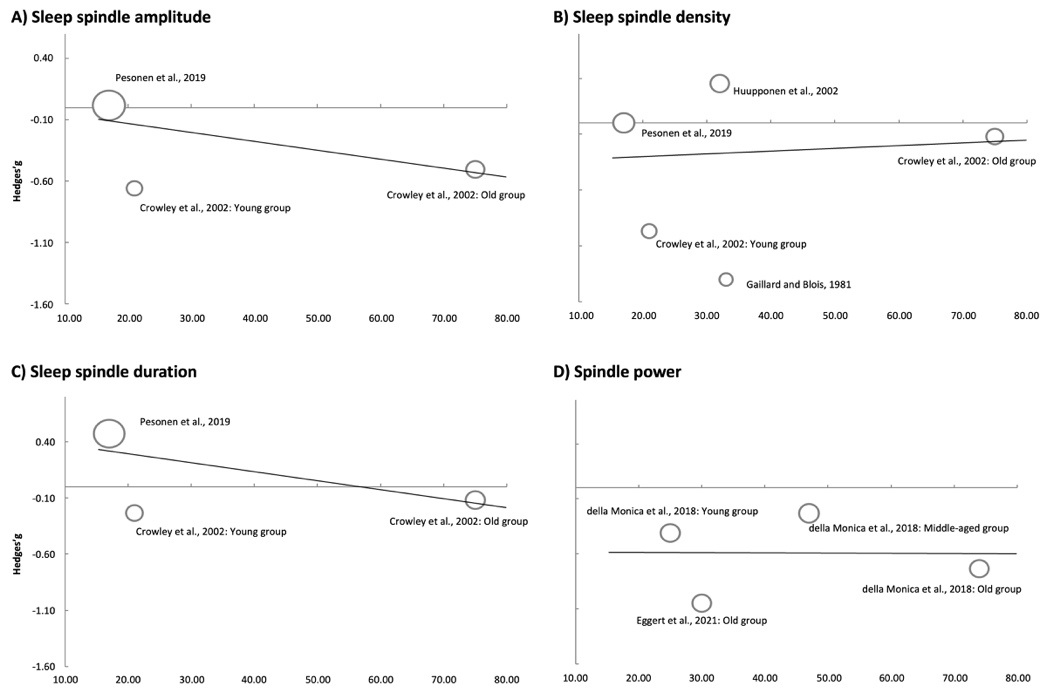
**

**Supplementary Table 24. Meta-regression analyses: Differences in the sleep spindles between sexes moderated by age.**

| **Sleep Spindles** | **Slope** | **95% CI** | **p_z_** | **R^2^** |
| --- | --- | --- | --- | --- |
| **Amplitude** | -0.007 | [-0.05, 0.04] | 0.461 | 35.21 |
| **Density** | 0.002 | [-0.04, 0.04] | 0.861 | 0.77 |
| **Duration** | -0.008 | [-0.05, 0.04] | 0.444 | 36.90 |
| **Sigma (µV^2^)** | -0.002 | [-0.04, 0.04] | 0.985 | 0.02 |

**6. Sleep spindles and cognitive ability**

Briefly, results shown in the Supplementary Tables 33-39 indicate the relation between cognitive ability and sleep spindles as averaged correlated coefficients, using the Fisher’s r-to-Z-transformation method (Fisher, 1921) and interpretation is as usual: results denote the direction (positive or negative) and the strength (magnitude) of the relation between cognitive ability measurement and each sleep spindle parameter.

**Supplementary Table 25. Main characteristics of the studies included in the meta-analysis about sleep spindles and cognitive ability** **in children.** Under subjects, the total sample, the sample number separated by sex and the age range are provided. Under Sleep Scoring, it is mentioned each scoring method followed by the authors. Under sleep spindles, the method of spindles detection, the electrode used for each type of spindle, the sleep stage used, and the frequency ranges are shown. Each test or scale for measuring cognitive abilities are shown under Cognitive Test. RK: Rechtschaffen and Kales (1968). AASM (American Academy of Sleep Medicine): Iber et al. (2007). ASK Analyzer: Automatic Detector by The Siesta Group, Vienna, Austria. IAM: Individual Adjusted Method (Bodizs et al., 2009). C: Central. S2: Sleep Stage 2, NREMS: Non-Rapid Eye Movement Sleep, SWS: Slow Waves Sleep. SBIS: Stanford-Binet Intelligence Scale. WISC: Wechsler Intelligence Scale for Children. CPM: Colored Progressive Matrices.

|  | Subjects | | | | Sleep | Sleep Spindles | | | | | |  |
| --- | --- | --- | --- | --- | --- | --- | --- | --- | --- | --- | --- | --- |
| Reference | **N** | **Females** | **Males** | **Age** | **Scoring** | **Detection** | **Electrodes** |  |  | **Stage** | **Frequencies** | **Test** |
| Chatburn et al., 2013 | 27 | 14 | 13 | 4-13 | RK | Visual | C3 |  |  | S2 | 11-15.9 Hz | SBIS |
| Geiger et al., 2011 | 14 | 6 | 8 | 9-13 | AASM | Spectral power | C3 |  |  | S2 | 12-15 Hz | WISC-IV |
| Gruber et al., 2013 | 29 | 14 | 15 | 7-11 | AASM | Schabus et al. (2007) | Several |  |  | NREMS | 11-15 Hz | WISC-IV |
| Hahn et al., 2018 | 34 | 24 | 10 | 8-11 | AASM | ASK analyzer | C3, Cz, C4 |  |  | S2 | 11-13 Hz  13-15 Hz | WISC |
| Hoedlmoser et al., 2014 | 54 | 25 | 29 | 8-11 | AASM | ASK analyzer | Cz |  |  | NREMS | 11-13 Hz | WISC-IV |
| Sulkamo et al., 2019 | 17 | 10 | 7 | 8.9-10.8 | AASM | Huupponen et al. (2007) | C3-C4 |  |  | NREMS | 11-15 Hz | WISC-III |
| Tessier et al., 2015 | 13 | 0 | 13 | 6-13 | AASM | Visual | C3 |  |  | S2 | 12-13 Hz  13.25-15.75 Hz | WISC-III |
| Ujma et al., 2016 | 28 | 15 | 13 | 4-8 | AASM | IAM | Cz |  |  | S2 and SWS | <11 Hz  11-13 Hz | CPM |

**Supplementary Table 26. Sleep spindles parameters published in each study investigating their relation to cognitive ability** **in children.** Dens: Density, Amp: Amplitude, Dur: Duration, Freq: Frequency, Sigma: spindle power.

|  | **Sleep Spindles** | | | **Fast Sleep Spindles** | | | | | **Slow Sleep Spindles** | | | | |
| --- | --- | --- | --- | --- | --- | --- | --- | --- | --- | --- | --- | --- | --- |
| **Reference** | **Dens** | **Freq** | **Sigma** | **Dens** | **Amp** | **Dur** | **Freq** | **Sigma** | **Dens** | **Amp** | **Dur** | **Freq** | **Sigma** |
| **Chatburn et al., 2013** |  |  |  | X |  | X | X |  | X |  | X | X |  |
| **Geiger et al., 2011** |  | X | X |  |  |  |  |  |  |  |  |  |  |
| **Gruber et al., 2013** |  | X |  |  |  |  |  |  |  |  |  |  |  |
| **Hahn et al., 2018** |  |  |  | X |  |  |  |  | X |  |  |  |  |
| **Hoedlmoser et al., 2014** |  |  |  | X | X | X | X |  | X | X | X | X |  |
| **Sulkamo et al., 2019** | X |  |  |  |  |  |  |  |  |  |  |  |  |
| **Tessier et al., 2015** |  |  | X |  |  |  |  | X |  |  |  |  | X |
| **Ujma et al., 2016** |  |  |  | X | X | X | X |  | X | X | X | X |  |

**Supplementary Table 27. Main characteristics of the studies included in the meta-analysis about sleep spindles and cognitive ability** **in adolescents.** Under subjects, the total sample, the sample number separated by sex and the age range are provided. Under Sleep Scoring, it is mentioned each scoring method followed by the authors. Under sleep spindles, the method of spindles detection, the electrode used for each type of spindle, the sleep stage used, and the frequency ranges are shown. Each test or scale for measuring cognitive abilities are shown under Cognitive Test. RK: Rechtschaffen and Kales (1968). AASM (American Academy of Sleep Medicine): Iber et al. (2007). IAM: Individual Adjustment Method (Bodizs et al., 2009). ASK Analyzer: Automatic detector, The Siesta Group, Vienna, Austria. PRANA: Automated spindle counter, PhiTools, Strasbourg, France. C: Central. S2: Sleep Stage 2, NREMS: Non-Rapid Eye Movement Sleep. RPMT: Raven Progressive Matrices Test. WAIS: Wechsler Adult Intelligence Scale. WISC: Wechsler Intelligence Scale for Children.

|  | Subjects | | | | Sleep | Sleep Spindles | | | | Cognitive |
| --- | --- | --- | --- | --- | --- | --- | --- | --- | --- | --- |
| Reference | **N** | **Females** | **Males** | **Age** | **Scoring** | **Detection** | **Electrodes** | **Stage** | **Frequencies** | **Test** |
| Bodizs et al., 2014 | 24 | 12 | 12 | 15-22 | RK | IAM | Cz | NREMS | 9-16 Hz | RPMT |
| Hahn et al., 2018 | 34 | 24 | 10 | 14-18 | AASM | ASK analyzer | C3, Cz, C4 | S2 | 11-13 Hz  13-15 Hz | WAIS |
| Nader and Smith, 2015 | 32 | 17 | 15 | 12-19 | RK | PRANA | C3-C4 | S2 | 11-13.5 Hz  13.5-16 Hz | WISC-IV/WAIS-III |
| Pesonen et al., 2019 | 176 | 100 | 76 | 17 | AASM | IAM | C3 | S2 | 10-13 Hz  13-16 Hz | WAIS-III |

**Supplementary Table 28. Sleep spindles parameters published in each study investigating their relation to cognitive ability** **in adolescents.** Dens: Density, Amp: Amplitude, Dur: Duration, Freq: Frequency.

|  | **Fast Sleep Spindles** | | | | **Slow Sleep Spindles** | | | |
| --- | --- | --- | --- | --- | --- | --- | --- | --- |
| **Reference** | **Dens** | **Amp** | **Dur** | **Freq** | **Dens** | **Amp** | **Dur** | **Freq** |
| **Bodizs et al., 2014** | X | X | X | X | X | X | X | X |
| **Hahn et al., 2018** | X |  |  |  | X |  |  |  |
| **Nader and Smith, 2015** | X |  |  |  | X |  |  |  |
| **Pesonen et al., 2019** | X | X | X |  | X | X | X |  |

**Supplementary Table 29. Main characteristics of the studies included in the meta-analysis about sleep spindles and cognitive ability** **in adults.** Under subjects, the total sample, the sample number separated by sex and the age are provided. Under Sleep Scoring, it is mentioned each scoring method followed by the authors. Under sleep spindles, the method of spindles detection, the electrode used for each type of spindle, the sleep stage used, and the frequency ranges are shown. Each test or scale for measuring cognitive abilities are shown under Cognitive Test. RK: Rechtschaffen and Kales (1968). AASM: American Academy of Sleep Medicine (Iber et al., 2007). IAM: Individual Adjusted Method (Bodizs et al., 2009). ISSD: Individual Sleep Spindle Detection method (Ray et al., 2015). PRANA: Automated spindle counter, PhiTools, Strasbourg, France (Ray et al., 2010). C: Central, F: Frontal, P: Parietal. S2: Sleep Stage 2, SWS: Slow Waves Sleep, NREMS: Non-Rapid Eye Movement Sleep. RPMT: Raven Progressive Matrices Test. CBS: Cambridge Brain Sciences Trials. MAB: Multidimensional Aptitude Battery. ZVT: Zahlen-Verbindungs-Test. APM: Advanced Progressive Matrices Test. CFT: Culture Fair Test.

|  | Subjects | | | | Sleep | Sleep Spindles | | | | | | Cognitive |
| --- | --- | --- | --- | --- | --- | --- | --- | --- | --- | --- | --- | --- |
| Reference | **N** | **Females** | **Males** | **Age** | **Scoring** | **Detection** | **Electrodes** | **FSS** | **SSS** | **Stage** | **Frequencies** | **Test** |
| Bodizs et al., 2005 | 19 | 5 | 14 | 27-47 (n = 18), 67 (n = 1) | RK | IAM |  | F4 | C3-C4 | S2 | 11-15 Hz | RPMT |
| Fang et al., 2017 | 27 | 18 | 9 | 19-40 | AASM | ISSD |  | Pz | Fz | S2 and SWS | 11-13.5 Hz  13.5-16 Hz | CBS |
| Fang et al., 2017B | 29 | 17 | 12 | 20-35 | AASM | ISSD | Cz | Pz | Fz | NREMS | 11-13.5 Hz  13.5-16 Hz | CBS |
| Fang et al., 2019 | 29 | 17 | 12 | 20-35 | AASM | ISSD | Cz | Pz | Fz | NREMS | 11-13.5 Hz  13.5-16 Hz | CBS |
| Fogel and Smith, 2006 | 12 | 12 | 0 | 20-25 | RK | Visual | C3-C4 |  |  | S2 | 12-16 Hz | MAB-II |
| Fogel et al., 2007: Study 1 | 10 | 10 | 0 | 18-29 | RK | Visual | C3-C4 |  |  | S2 | 12-16 Hz | MAB-II |
| Fogel et al., 2007: Study 2 | 12 | 12 | 0 | 20-25 | RK | Visual | C3-C4 |  |  | S2 | 12-16 Hz | MAB-II |
| Fogel et al., 2007: Study 3 | 35 | 29 | 6 | 18-26 | RK | Visual | Cz |  |  | SWS | 12-14 Hz  14-16 Hz | MAB-II |
| Lustenberger et al., 2012 | 15 | 0 | 15 | 18-20 | AASM | Spectral power | C4 |  |  | NREMS | 12-15 Hz | ZVT |
| Peters et al., 2007 | 24 | 12 | 12 | 20.54±2.40 | RK | Visual | C3-C4 |  |  | S2 | 12-16 Hz | MAB-II |
| Peters et al., 2008: Young group | 14 | 7 | 7 | 17-24 | RK | Visual | C3-C4 |  |  | S2 | 12-16 Hz | MAB-II |
| Schabus et al., 2006 | 48 | 24 | 24 | 20-30 | RK | Anderer et al. (2005) | C3-C4 |  |  | S2 | 11-13 Hz  13-15 Hz | APM |
| Tucker and Fishbein, 2009 | 24 | 12 | 12 | 20.9 |  | Spectral power | C3-C4 |  |  | S2 | 12-15 Hz | MAB-II |
| Ujma et al., 2014 | 160 | 72 | 88 | 17-69 | AASM | IAM | Cz |  |  | S2 | 11-13 Hz  13-15 Hz | APM/CFT |
| Ujma et al., 2015: nap study | 79 | 0 | 79 | 18-30 | AASM | IAM | C3-C4 |  |  | S2 | Individual | CFT 20-R |
| Ward et al., 2014 | 30 | 21 | 9 | 18-29 | RK | PRANA software | C3 |  |  | S2 | 12-16 Hz | MAB-II |

**Supplementary Table 30. Sleep spindles parameters published in each study investigating their relation to cognitive ability** **in adults.** Dens: Density, Amp: Amplitude, Sigma: spindle power, Dur: Duration, Freq: Frequency.

|  | **Sleep Spindles** | | | | **Fast Sleep Spindles** | | | | **Slow Sleep Spindles** | | | |
| --- | --- | --- | --- | --- | --- | --- | --- | --- | --- | --- | --- | --- |
| **Reference** | **Dens** | **Amp** | **Count** | **Sigma** | **Dens** | **Amp** | **Dur** | **Freq** | **Dens** | **Amp** | **Dur** | **Freq** |
| **Bodizs et al., 2005** |  |  |  |  | X |  |  |  | X |  |  |  |
| **Fang et al., 2017** |  |  |  |  | X | X | X |  | X | X | X |  |
| **Fang et al., 2017B** |  |  |  |  | X | X | X |  | X | X | X |  |
| **Fang et al., 2019** |  | X |  |  |  |  |  |  |  |  |  |  |
| **Fogel and Smith, 2006** |  |  | X |  |  |  |  |  |  |  |  |  |
| **Fogel et al., 2007: Study 1** |  |  | X | X |  |  |  |  |  |  |  |  |
| **Fogel et al., 2007: Study 2** |  |  | X | X |  |  |  |  |  |  |  |  |
| **Fogel et al., 2007: Study 3** |  |  | X |  |  |  |  |  |  |  |  |  |
| **Lustenberger et al., 2012** |  |  |  | X |  |  |  |  |  |  |  |  |
| **Peters et al., 2007** | X |  |  |  |  |  |  |  |  |  |  |  |
| **Peters et al., 2008: Young group** | X |  |  |  |  |  |  |  |  |  |  |  |
| **Schabus et al., 2006** |  |  |  |  | X | X | X |  | X | X | X |  |
| **Tucker and Fishbein, 2009** |  |  |  | X |  |  |  |  |  |  |  |  |
| **Ujma et al., 2014** |  |  |  |  | X | X | X | X | X | X | X | X |
| **Ujma et al., 2015** |  |  |  |  | X | X | X | X | X | X | X | X |
| **Ward et al., 2014** | X |  |  |  |  |  |  |  |  |  |  |  |

**Supplementary Table 31. Main characteristics of the studies included in the meta-analysis about sleep spindles and cognitive ability** **in elderly.** Under subjects, the total sample, the sample number separated by sex and the age are provided. Under Sleep Scoring, it is mentioned each scoring method followed by the authors. Under sleep spindles, the method of spindles detection, the electrode used for each type of spindle, the sleep stage used, and the frequency ranges are shown. Each test or scale for measuring cognitive abilities are shown under Cognitive Test. RK: Rechtschaffen and Kales (1968). AASM (American Academy of Sleep Medicine): Iber et al. (2007). MSS: Michele Sleep Scoring Software, Cerebra Health. C: Central, F: Frontal. S2: Sleep Stage 2. D-KEFS: Delis-Kaplan Executive Function System. MAB: Multidimensional Aptitude Battery.

|  | Subjects | | | | Sleep | Sleep Spindles | | | | | | Cognitive |
| --- | --- | --- | --- | --- | --- | --- | --- | --- | --- | --- | --- | --- |
| Reference | **N** | **Females** | **Males** | **Age** | **Scoring** | **Detection** | **Electrodes** | **FSS** | **SSS** | **Stage** | **Frequencies** | **Test** |
| Guadagni et al., 2021 | 63 | 31 | 32 | 68.2±5.6 | AASM | MSS | C3-C4 | C3-C4 | F3-F4 | S2 | 10-12 Hz  12-16 Hz | D-KEFS |
| Peters et al., 2008: Old group | 14 | 7 | 7 | 62-79 | RK | Visual | C3-C4 |  |  | S2 | 12-16 Hz | MAB-II |

**Supplementary Table 32. Sleep spindles parameters published in each study investigating their relation to cognitive ability** **in elderly.**

|  | **Sleep Spindles** | **Fast Sleep Spindles** | **Slow Sleep Spindles** |
| --- | --- | --- | --- |
| **Reference** | **Density** | **Density** | **Density** |
| **Guadagni et al., 2021** | X | X | X |
| **Peters et al., 2008: Old group** | X |  |  |

**Supplementary Table 33.** **Meta-analytic results of the correlation between sleep spindles and cognitive ability** **in all ages.** k = number of studies. N = total sample. The combined correlation in both random- and fixed-effects models is denoted as r. Square brackets show the lower and upper limits from the confidence intervals (95%). p_r_ represents the p-vaue from the combined correlation. Heterogeneity is shown by the Q (Cochran’s Q and its p-value: p_Q_), I^2^, Tau and T^2^ statistics. Egger’s (p): Egger’s regression’s p-value. Begg (p): Begg and Mazumbar correlation’s p-value. Significances are presented in bold. Amp: amplitude, Dens: density, Freq: frequency. Sigma: spindle power, FSS power: absolute fast sleep spindles power, SSS power: slow sleep spindles power. NC: not sufficient data to calculate.

|  | | | **Random effects** | | **Fixed effects** | | **Heterogeneity** | | | | | **Publication bias** | |  |
| --- | --- | --- | --- | --- | --- | --- | --- | --- | --- | --- | --- | --- | --- | --- |
| **Sleep Spindles** | **k** | **N** | **r [95% CI]** | **p_r_** | **r [95% CI]** | **p_r_** | **Q** | **p_Q_** | **I^2^** | **Tau** | **T^2^** | **Egger’s (p)** | **Begg (p)** | |
| **Amp** | 1 | 27 |  |  | 0.435 [NC] | **0.002** |  |  |  |  |  |  |  | |
| **Dens** | 6 | 162 | 0.17 [-0.18, 0.47] | 0.220 | 0.24 [0.03, 0.43] | **0.004** | 11.39 | **0.044** | 56.11 | 0.24 | 0.06 | 0.22 | 0.260 | |
| **Freq** | 2 | 43 | -0.57 [-0.67, -0.45] | **0.000** | -0.57 [-0.99, 0.89] | **0.00009** | 0.01 | 0.938 | 0.000 | 0.000 | 0.000 | NC | 0.317 | |
| **Count** | 4 | 69 | 0.61 [-0.10, 0.91] | **0.005** | 0.43 [0.04, 0.71] | **0.001** | 12.40 | **0.006** | 75.80 | 0.52 | 0.27 | **0.04** | 0.308 | |
| **Sigma** | 6 | 88 | 0.49 [0.06, 0.76] | **0.004** | 0.41 [0.13, 0.63] | **0.0002** | 13.67 | **0.018** | 63.42 | 0.39 | 0.15 | **0.02** | 0.091 | |
| **Fast sleep spindles** | **k** | **N** | **r [95% CI]** | **p_r_** | **r [95% CI]** | **p_r_** | **Q** | **p_Q_** | **I^2^** | **Tau** | **T^2^** | **Egger’s (p)** | **Begg (p)** | |
| **Amp** | 9 | 625 | 0.18 [0.10, 0.25] | **0.0000004** | 0.18 [0.08, 0.27] | **0.00001** | 5.92 | 0.657 | 0.00 | 0.00 | 0.00 | 0.14 | 0.095 | |
| **Dens** | 15 | 834 | 0.04 [-0.12, 0.21] | 0.577 | 0.01 [-0.07, 0.08] | 0.855 | 43.18 | **0.00008** | 67.57 | 0.20 | 0.04 | 0.21 | 0.181 | |
| **Dur** | 10 | 652 | 0.04 [-0.04, 0.12] | 0.303 | 0.04 [-0.05, 0.13] | 0.354 | 7.29 | 0.607 | 0.00 | 0.00 | 0.00 | 0.49 | 0.371 | |
| **Freq** | 6 | 372 | -0.05 [-0.14, 0.05] | 0.206 | -0.05 [-0.18, 0.09] | 0.366 | 2.56 | 0.768 | 0.00 | 0.00 | 0.00 | 0.63 | 0.188 | |
| **FSS power** | 1 | 13 |  |  | 0.33 [NC] | 0.278 |  |  |  |  |  |  |  | |
| **Slow sleep spindles** | **k** | **N** | **r [95% CI]** | **p_r_** | **r [95% CI]** | **p_r_** | **Q** | **p_Q_** | **I^2^** | **Tau** | **T^2^** | **Egger’s (p)** | **Begg (p)** | |
| **Amp** | 9 | 625 | 0.09 [-0.06, 0.24] | 0.160 | 0.04 [-0.05, 0.14] | 0.296 | 18.08 | **0.021** | 55.75 | 0.14 | 0.02 | 0.17 | 0.211 | |
| **Dens** | 15 | 834 | 0.11 [-0.01, 0.22] | **0.044** | 0.12 [0.04, 0.19] | **0.001** | 28.61 | **0.012** | 51.07 | 0.15 | 0.02 | 0.08 | **0.015** | |
| **Dur** | 10 | 652 | 0.07 [-0.05, 0.18] | 0.210 | 0.07 [-0.05, 0.18] | 0.095 | 13.38 | 0.146 | 32.75 | 0.09 | 0.01 | 0.33 | 0.283 | |
| **Freq** | 6 | 372 | -0.03 [-0.16, 0.09] | 0.518 | -0.03 [-0.17, 0.10] | 0.551 | 4.25 | 0.514 | 0.00 | 0.00 | 0.00 | 0.81 | 0.573 | |
| **SSS power** | 1 | 13 |  |  | 0.27 [NC] | 0.381 |  |  |  |  |  |  |  | |

**Supplementary Table 34. Meta-analytic results of the correlation between sleep spindles and cognitive ability** **in children.** k = number of studies. N = total sample. The combined correlation in both random- and fixed-effects models is denoted as r. Square brackets show the lower and upper limits from the confidence intervals (95%). p_r_ represents the p-vaue from the combined correlation. Heterogeneity is shown by the Q (Cochran’s Q and its p-value: p_Q_), I^2^, Tau and T^2^ statistics. Egger’s (p): Egger’s regression’s p-value. Begg (p): Begg and Mazumbar correlation’s p-value. Significances are presented in bold. Amp: amplitude, Dens: density, Dur: duration, Freq: frequency, Sigma: spindle power, FSS power: absolute fast sleep spindles power. NC: not sufficient data to calculate.

|  | | | **Random effects** | | | **Fixed effects** | | **Heterogeneity** | | | | | **Publication bias** | |  |
| --- | --- | --- | --- | --- | --- | --- | --- | --- | --- | --- | --- | --- | --- | --- | --- |
| **Sleep Spindles** | **k** | **N** | **r [95% CI]** | | **p_r_** | **r [95% CI]** | **p_r_** | **Q** | **p_Q_** | **I^2^** | **Tau** | **T^2^** | **Egger’s (p)** | **Begg (p)** | |
| **Dens** | 1 | 17 |  |  |  | 0.51 [NC] | **0.035** |  |  |  |  |  |  |  | |
| **Freq** | 2 | 43 | -0.57 [-0.67, -0.45] | | **0.000** | -0.57 [-0.99, 0.89] | **0.00009** | 0.01 | 0.938 | 0.00 | 0.00 | 0.00 | NC | 0.317 | |
| **Sigma** | 2 | 27 | 0.53 [-0.98, 1] | | **0.012** | 0.53 [-0.98, 1,00] | **0.007** | 1.15 | 0.284 | 12.80 | 0.12 | 0.01 | NC | 0.317 | |
| **Fast sleep spindles** | **k** | **N** | **r [95% CI]** | | **p_r_** | **r [95% CI]** | **p_r_** | **Q** | **p_Q_** | **I^2^** | **Tau** | **T^2^** | **Egger’s (p)** | **Begg (p)** | |
| **Amp** | 2 | 82 | 0.21 [-0.47, 0.73] | | **0.0002** | 0.21 [-0.85, 0.93] | 0.069 | 0.25 | 0.619 | 0.00 | 0.00 | 0.00 | NC | 0.317 | |
| **Dens** | 4 | 143 | -0.08 [-0.30, 0.14] | | 0.259 | -0.08 [-0.34, 0.20] | 0.361 | 1.96 | 0.581 | 0.00 | 0.00 | 0.00 | 0.30 | 0.497 | |
| **Dur** | 3 | 109 | 0.002 [-0.05, 0.05] | | 0.892 | 0.002 [-0.40, 0.41] | 0.988 | 0.02 | 0.988 | 0.00 | 0.00 | 0.00 | 0.95 | 0.602 | |
| **Freq** | 3 | 109 | -0.05 [-0.14, 0.05] | | **0.045** | -0.05 [-0.44, 0.37] | 0.647 | 0.10 | 0.949 | 0.00 | 0.00 | 0.00 | 0.48 | 0.117 | |
| **FSS power** | 1 | 13 |  | |  | 0.33 [NC] | 0.278 |  |  |  |  |  |  |  | |
| **Slow sleep spindles** | **k** | **N** | **r [95% CI]** | | **p_r_** | **r [95% CI]** | **p_r_** | **Q** | **p_Q_** | **I^2^** | **Tau** | **T^2^** | **Egger’s (p)** | **Begg (p)** | |
| **Amp** | 2 | 82 | 0.29 [-0.61, 0.86] | | **0.0002** | 0.29 [-0.82, 0.94] | 0.010 | 0.48 | 0.489 | 0.00 | 0.00 | 0.00 | NC | 0.317 | |
| **Dens** | 4 | 143 | 0.07 [-0.32, 0.44] | | 0.582 | 0.11 [-0.17, 0.37] | 0.211 | 6.76 | 0.080 | 55.63 | 0.20 | 0.04 | **0.07** | 0.174 | |
| **Dur** | 3 | 109 | 0.14 [-0.30, 0.53] | | 0.172 | 0.15 [-0.27, 0.52] | 0.135 | 2.21 | 0.331 | 9.53 | 0.06 | 0.00 | 0.14 | 0.602 | |
| **Freq** | 3 | 109 | -0.15 [-0.44, 0.16] | | **0.036** | -0.15 [-0.53, 0.27] | 0.122 | 1.09 | 0.581 | 0.00 | 0.00 | 0.00 | **0.07** | 0.117 | |
| **SSS power** | 1 | 13 |  | |  | 0.27 [NC] | 0.381 |  |  |  |  |  |  |  | |

**Supplementary Table 35. Meta-analytic results of the correlation between sleep spindles and cognitive ability** **in adolescents.** k = number of studies. N = total sample. The combined correlation in both random- and fixed-effects models is denoted as r. Square brackets show the lower and upper limits from the confidence intervals (95%). p_r_ represents the p-vaue from the combined correlation. Heterogeneity is shown by the Q (Cochran’s Q and its p-value: p_Q_), I^2^, Tau and T^2^ statistics. Egger’s (p): Egger’s regression’s p-value. Begg (p): Begg and Mazumbar correlation’s p-value. Significances are presented in bold. Amp: amplitude, Dens: density, Dur: duration, Freq: frequency. NC: not sufficient data to calculate.

|  | | | **Random effects** | | **Fixed effects** | | **Heterogeneity** | | | | | **Publication bias** | |  |
| --- | --- | --- | --- | --- | --- | --- | --- | --- | --- | --- | --- | --- | --- | --- |
| **Fast Sleep Spindles** | **k** | **N** | **r [95% CI]** | **p_r_** | **r [95% CI]** | **p_r_** | **Q** | **p_Q_** | **I^2^** | **Tau** | **T^2^** | **Egger’s (p)** | **Begg (p)** | |
| **Amp** | 2 | 200 | 0.21 [-0.58, 0.80] | **0.002** | 0.21 [-0.61, 0.81] | **0.003** | 0.93 | 0.336 | 0.00 | 0.00 | 0.00 | NC | 0.317 | |
| **Dens** | 4 | 266 | -0.07 [-0.28, 0.14] | 0.300 | -0.07 [-0.26, 0.13] | 0.258 | 3.15 | 0.369 | 4.78 | 0.03 | 0.00 | 0.84 | 0.497 | |
| **Dur** | 2 | 200 | -0.0002 [-0.72, 0.72] | 0.997 | -0.0002 [-0.72, 0.72] | 0.997 | 0.98 | 0.322 | 0.00 | 0.00 | 0.00 | NC | 0.317 | |
| **Freq** | 1 | 24 |  |  | 0.16 [NC] | 0.451 |  |  |  |  |  |  |  | |
| **Slow sleep spindles** | **k** | **N** | **r [95% CI]** | **p_r_** | **r [95% CI]** | **p_r_** | **Q** | **p_Q_** | **I^2^** | **Tau** | **T^2^** | **Egger’s (p)** | **Begg (p)** | |
| **Amp** | 2 | 200 | 0.05 [-0.48, 0.55] | 0.289 | 0.05 [-0.70, 0.74] | 0.507 | 0.39 | 0.531 | 0.00 | 0.00 | 0.00 | NC | 0.317 | |
| **Dens** | 4 | 266 | -0.004 [-0.19, 0.18] | 0.947 | -0.004 [-0.20, 0.19] | 0.950 | 2.68 | 0.444 | 0.00 | 0.00 | 0.00 | 0.63 | 1 | |
| **Dur** | 2 | 200 | -0.05 [-0.52, 0.44] | 0.195 | -0.05 [-0.75, 0.70] | 0.457 | 0.33 | 0.566 | 0.00 | 0.00 | 0.00 | NC | 0.317 | |
| **Freq** | 1 | 24 |  |  | 0.18 [NC] | 0.399 |  |  |  |  |  |  |  | |

**Supplementary Table 36. Meta-analytic results of the correlation between sleep spindles and cognitive ability** **in adults.** k = number of studies. N = total sample. The combined correlation in both random- and fixed-effects models is denoted as r. Square brackets show the lower and upper limits from the confidence intervals (95%). p_r_ represents the p-vaue from the combined correlation. Heterogeneity is shown by the Q (Cochran’s Q and its p-value: p_Q_), I^2^, Tau and T^2^ statistics. Egger’s (p): Egger’s regression’s p-value. Begg (p): Begg and Mazumbar correlation’s p-value. Significances are presented in bold. Amp: amplitude, Dens: density, Dur: duration, Freq: frequency. Sigma: spindle power.

|  | | | **Random effects** | | **Fixed effects** | | **Heterogeneity** | | | | | **Publication bias** | |  |
| --- | --- | --- | --- | --- | --- | --- | --- | --- | --- | --- | --- | --- | --- | --- |
| **Sleep spindles** | **k** | **N** | **r [95% CI]** | **p_r_** | **r [95% CI]** | **p_r_** | **Q** | **p_Q_** | **I^2^** | **Tau** | **T^2^** | **Egger’s (p)** | **Begg (p)** | |
| **Amp** | 1 | 27 |  | | 0.435 [NC] | **0.002** |  |  |  |  |  |  |  | |
| **Dens** | 3 | 68 | 0.01 [-0.53, 0.55] | 0.957 | 0.02 [-0.50,0.52] | 0.906 | 2.31 | 13.43 | 13.43 | 0.09 | 0.01 | 0.36 | 0.117 | |
| **Count** | 4 | 69 | 0.61 [-0.10, 0.91] | **0.005** | 0.43 [0.04, 0.71] | **0.001** | 12.40 | **0.006** | 75.80 | **0.52** | **0.27** | **0.04** | 0.308 | |
| **Sigma** | 4 | 61 | 0.48 [-0.32, 0.88] | **0.05** | 0.36 [-0.08, 0.68] | **0.009** | 11.84 | **0.008** | 74.67 | **0.51** | **0.26** | **0.05** | **0.042** | |
| **Fast sleep spindles** | **k** | **N** | **r [95% CI]** | **p_r_** | **r [95% CI]** | **p_r_** | **Q** | **p_Q_** | **I^2^** | **Tau** | **T^2^** | **Egger’s (p)** | **Begg (p)** | |
| **Amp** | 5 | 343 | 0.15 [-0.01, 0.30] | **0.008** | 0.15 [0.00, 0.30] | **0.006** | 4.22 | 0.378 | 5.13 | 0.03 | 0.00 | 0.39 | 0.327 | |
| **Dens** | 6 | 362 | 0.12 [-0.32, 0.53] | 0.487 | 0.02 [-0.12, 0.16] | 0.671 | 24.79 | **0.0001** | 79.83 | 0.28 | 0.08 | 0.17 | 0.188 | |
| **Dur** | 5 | 343 | 0.08 [-0.12, 0.27] | 0.265 | 0.07 [-0.08, 0.22] | 0.204 | 5.53 | 0.237 | 27.71 | 0.08 | 0.01 | 0.96 | 1 | |
| **Freq** | 2 | 239 | -0.08 [-0.8, 0.74] | 0.341 | -0.07 [-0.72, 0.64] | 0.298 | 1.41 | 0.235 | 29.06 | 0.06 | 0.00 | NC | 0.317 | |
| **Slow sleep spindles** | **k** | **N** | **r [95% CI]** | **p_r_** | **r [95% CI]** | **p_r_** | **Q** | **p_Q_** | **I^2^** | **Tau** | **T^2^** | **Egger’s (p)** | **Begg (p)** | |
| **Amp** | 5 | 343 | 0.04 [-0.23, 0.30] | 0.711 | -0.02 [-0.17, 0.13] | 0.730 | 11.03 | **0.026** | 63.73 | 0.18 | 0.03 | 0.54 | 0.142 | |
| **Dens** | 6 | 362 | 0.14 [0.01, 0.27] | **0.005** | 0.14 [0.00, 0.27] | **0.009** | 4.44 | 0.488 | 0.00 | 0.00 | 0.00 | **0.01** | **0.039** | |
| **Dur** | 5 | 343 | 0.10 [-0.13, 0.31] | 0.242 | 0.11 [-0.04, 0.26] | **0.041** | 6.66 | 0.155 | 39.93 | 0.11 | 0.01 | 0.25 | 0.327 | |
| **Freq** | 2 | 239 | 0.002 [-0.49, 0.49] | 0.969 | 0.002 [-0.68, 0.68] | 0.980 | 0.42 | 0.581 | 0.00 | 0.00 | 0.00 | NC | 0.317 | |

**Supplementary Table 37. Meta-analytic results of the correlation between sleep spindles and cognitive ability** **in older adults.** k = number of studies. N = total sample. The combined correlation in both random- and fixed-effects models is denoted as r. Square brackets show the lower and upper limits from the confidence intervals (95%). p_r_ represents the p-vaue from the combined correlation. Heterogeneity is shown by the Q (Cochran’s Q and its p-value: p_Q_), I^2^, Tau and T^2^ statistics. Egger’s (p): Egger’s regression’s p-value. Begg (p): Begg and Mazumbar correlation’s p-value. Significances are presented in bold. Dens: density.

|  | | | **Random effects** | | **Fixed effects** | | **Heterogeneity** | | | | | **Publication bias** | |  |
| --- | --- | --- | --- | --- | --- | --- | --- | --- | --- | --- | --- | --- | --- | --- |
| **Sleep Spindles** | **k** | **N** | **r [95% CI]** | **p_r_** | **r [95% CI]** | **p_r_** | **Q** | **p_Q_** | **I^2^** | **Tau** | **T^2^** | **Egger’s (p)** | **Begg (p)** | |
| **Dens** | 2 | 77 | 0.21 [-1, 1] | 0.472 | 0.35 [-0.81, 0.95] | **0.002** | 3.50 | 0.061 | 71.42 | 0.37 | 0.13 | NC | 0.317 | |
| **Fast sleep spindles** | **k** | **N** | **r [95% CI]** | **p_r_** | **r [95% CI]** | **p_r_** | **Q** | **p_Q_** | **I^2^** | **Tau** | **T^2^** | **Egger’s (p)** | **Begg (p)** | |
| **Dens** | 1 | 63 |  |  | 0.40 [NC] | **0.001** |  |  |  |  |  |  |  | |
| **Slow sleep spindles** | **k** | **N** | **r [95% CI]** | **p_r_** | **r [95% CI]** | **p_r_** | **Q** | **p_Q_** | **I^2^** | **Tau** | **T^2^** | **Egger’s (p)** | **Begg (p)** | |
| **Dens** | 1 | 63 |  |  | 0.49 [NC] | **0.000** |  |  |  |  |  |  |  | |

**Supplementary Figure 6. Publication bias from the correlation between sleep spindles and cognitive ability.** The funnel plots represent the correlation (z-transformed, Y-axis), the standard deviation (SD, z-transformed, X-axis) for each sleep spindle parameter and the reference of the outlier points. The combined effect size is depicted at the base of the funnel. Moreover, the adjusted combined effect size is provided below. When the trim and fill method was used, the studies are represented with a white dot.

**
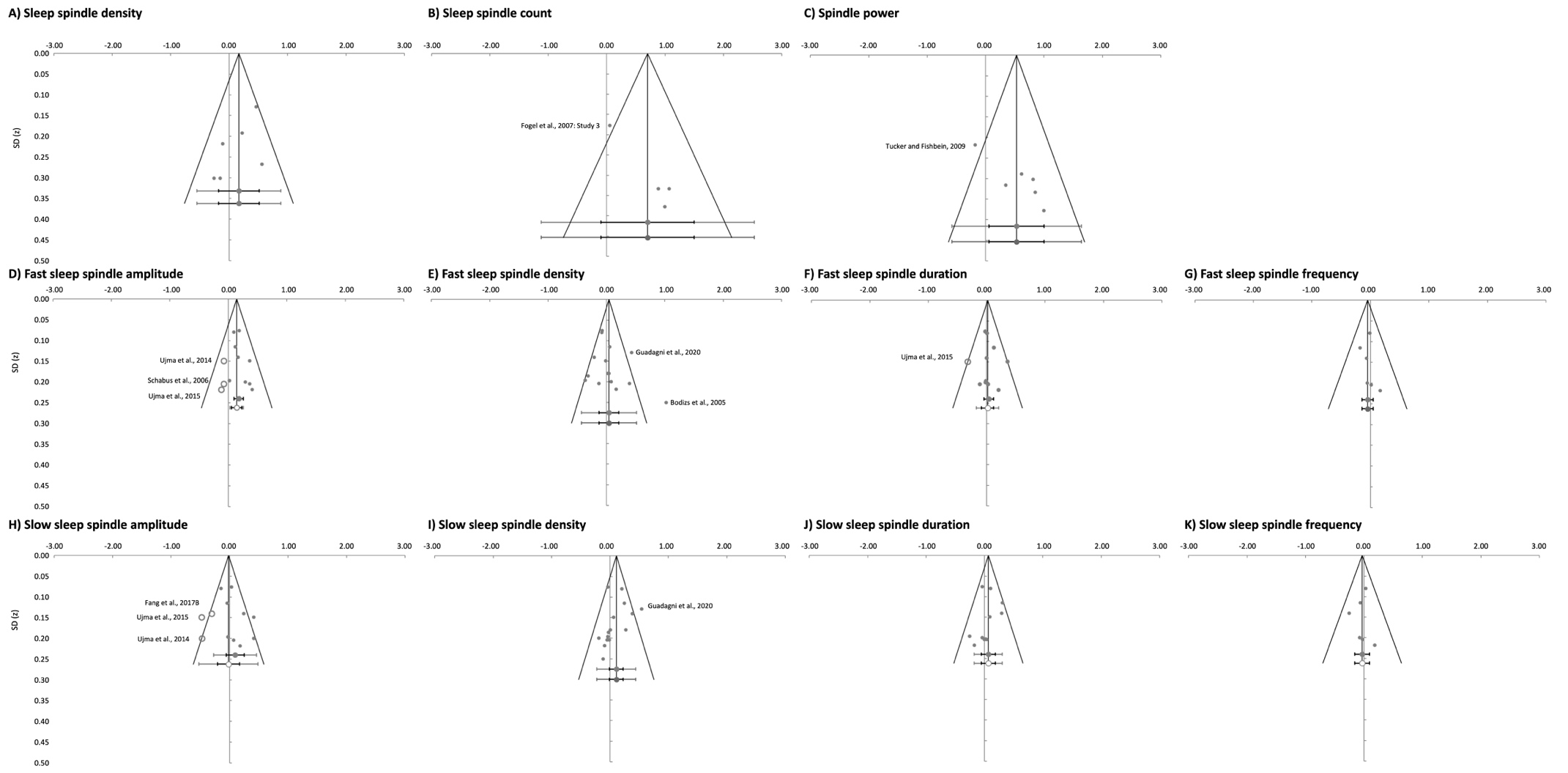
**

**Supplementary Table 38.** **Sensitivity analyses:** **Meta-analytic results of the correlation between sleep spindles and cognitive ability without the approximated Pearson correlations.** k = number of studies. N = total sample. The combined correlation in both random- and fixed-effects models is denoted as r. Square brackets show the lower and upper limits from the confidence intervals (95%). p_r_ represents the p-vaue from the combined correlation. Heterogeneity is shown by the Q (Cochran’s Q and its p-value: p_Q_), I^2^, Tau and T^2^ statistics. Egger’s (p): Egger’s regression’s p-value. Begg (p): Begg and Mazumbar correlation’s p-value. Significances are presented in bold. Dens: density, Freq: frequency, Sigma: spindle power.

|  |  |  | **Random effects** | | | **Fixed effects** | | **Heterogeneity** | | | | | **Publication bias** | |  |
| --- | --- | --- | --- | --- | --- | --- | --- | --- | --- | --- | --- | --- | --- | --- | --- |
| **Sleep spindles** | **k** | **N** | **r [95% CI]** | | **p_r_** | **r [95% CI]** | **p_r_** | **Q** | **p_Q_** | **I^2^** | **Tau** | **T^2^** | **Egger’s (p)** | **Begg (p)** | |
| **Dens** | 5 | 145 | 0.10 [-0.28, 0.45] | | 0.478 | 0.20 [-0.04, 0.42] | **0.018** | 9.80 | **0.044** | 59.18 | **0.25** | **0.06** | **0.03** | 0.086 | |
| **Freq** | 1 | 29 |  |  |  | -0.56 [NC] | **0.001** |  |  |  |  |  |  |  | |
| **Sigma** | 5 | 74 | 0.45 [-0.11, 0.79] | | **0.025** | 0.35 [0.01, 0.62] | **0.005** | 11.85 | **0.018** | 66.25 | **0.42** | **0.17** | **0.03** | **0.05** | |
| **Fast sleep spindles** | **k** | **N** | **r [95% CI]** | | **p_r_** | **r [95% CI]** | **p_r_** | **Q** | **p_Q_** | **I^2^** | **Tau** | **T^2^** | **Egger’s (p)** | **Begg (p)** | |
| **Dens** | 13 | 766 | 0.05 [-0.15, 0.24] | | 0.603 | 0.004 [-0.08, 0.08] | 0.908 | 43.13 | **0.00002** | 72.18 | 0.22 | 0.05 | 0.23 | 0.2 | |
| **Slow sleep spindles** | **k** | **N** | **r [95% CI]** | | **p_r_** | **r [95% CI]** | **p_r_** | **Q** | **p_Q_** | **I^2^** | **Tau** | **T^2^** | **Egger’s (p)** | **Begg (p)** | |
| **Dens** | 13 | 766 | 0.10 [-0.03, 0.23] | | 0.090 | 0.12 [0.04, 0.20] | **0.001** | 27.52 | **0.006** | 56.40 | 0.16 | 0.02 | 0.07 | 0.077 | |

**Supplementary Table 39.** **Sensitivity analyses:** **Meta-analytic results of the correlation between fast and slow sleep spindles and cognitive ability without the nap study (Ujma et al., 2015).** k = number of studies. N = total sample. The combined correlation in both random- and fixed-effects models is denoted as r. Square brackets show the lower and upper limits from the confidence intervals (95%). p_r_ represents the p-vaue from the combined correlation. Heterogeneity is shown by the Q (Cochran’s Q and its p-value: p_Q_), I^2^, Tau and T^2^ statistics. Egger’s (p): Egger’s regression’s p-value. Begg (p): Begg and Mazumbar correlation’s p-value. Significances are presented in bold. Amp: amplitude, Dens: density, Dur: duration, Freq: frequency. NC: not sufficient data to calculate

|  | | | **Random effects** | | **Fixed effects** | | **Heterogeneity** | | | | | **Publication bias** | |  |
| --- | --- | --- | --- | --- | --- | --- | --- | --- | --- | --- | --- | --- | --- | --- |
| **Fast sleep spindles** | **k** | **N** | **r [95% CI]** | **p_r_** | **r [95% CI]** | **p_r_** | **Q** | **p_Q_** | **I^2^** | **Tau** | **T^2^** | **Egger’s (p)** | **Begg (p)** | |
| **Amp** | 8 | 546 | 0.18 [0.09, 0.27] | **0.000002** | 0.18 [0.08, 0.28] | **0.00002** | 5.60 | 0.587 | 0.00 | 0.00 | 0.00 | 0.16 | 0.138 | |
| **Dens** | 14 | 755 | 0.04 [-0.14, 0.23] | 0.604 | 0.001 [-0.08, 0.08] | 0.974 | 42.97 | **0.00004** | 69.75 | 0.22 | 0.05 | 0.20 | 0.112 | |
| **Dur** | 9 | 573 | 0.03 [-0.06, 0.11] | 0.520 | 0.03 [-0.07, 0.12] | 0.557 | 6.65 | 0.575 | 0.00 | 0.00 | 0.00 | 0.49 | 0.251 | |
| **Freq** | 5 | 293 | -0.01 [-0.09, 0.07] | 0.669 | -0.01 [-0.18, 0.15] | 0.842 | 0.87 | 0.928 | 0.00 | 0.00 | 0.00 | 0.58 | 0.142 | |
| **Slow sleep spindles** | **k** | **N** | **r [95% CI]** | **p_r_** | **r [95% CI]** | **p_r_** | **Q** | **p_Q_** | **I^2^** | **Tau** | **T^2^** | **Egger’s (p)** | **Begg (p)** | |
| **Amp** | 8 | 546 | 0.12 [-0.05, 0.28] | 0.104 | 0.05 [-0.05, 0.16] | 0.209 | 17.47 | **0.015** | 59.93 | 0.16 | 0.03 | 0.25 | 0.458 | |
| **Dens** | 14 | 755 | 0.09 [-0.03, 0.21] | 0.102 | 0.11 [0.03, 0.19] | **0.004** | 27.30 | **0.011** | 52.38 | 0.15 | 0.02 | 0.11 | **0.019** | |
| **Dur** | 9 | 573 | 0.03 [-0.07, 0.14] | 0.455 | 0.03 [-0.06, 0.13] | 0.432 | 8.43 | 0.393 | 5.05 | 0.03 | 0.00 | 0.56 | 0.466 | |
| **Freq** | 5 | 293 | -0.03 [-0.20, 0.15] | 0.668 | -0.02 [-0.19, 0.14] | 0.687 | 4.18 | 0.383 | 4.21 | 0.03 | 0.00 | 0.86 | 0.624 | |

**S****upplementary Figure 7. Meta-regression analyses: Linear association between sleep spindle properties and age moderated by the percentage of females in each study.** Bubble plots depict the fitted meta-regression slope (ß) (z-transformed data), the mean age in each study (X-axis) and the reference of the outlier points. The size of the bubbles represents the weight. Bubble plots showing the linear meta-regression between cognitive ability and spindle characteristics (density or duration), with the percentage of female participants as moderator. Circle size reflects study precision, the solid line the fitted regression, and the shaded area the 95% CI. R² indicates the proportion of between-study heterogeneity explained by the moderator.

**
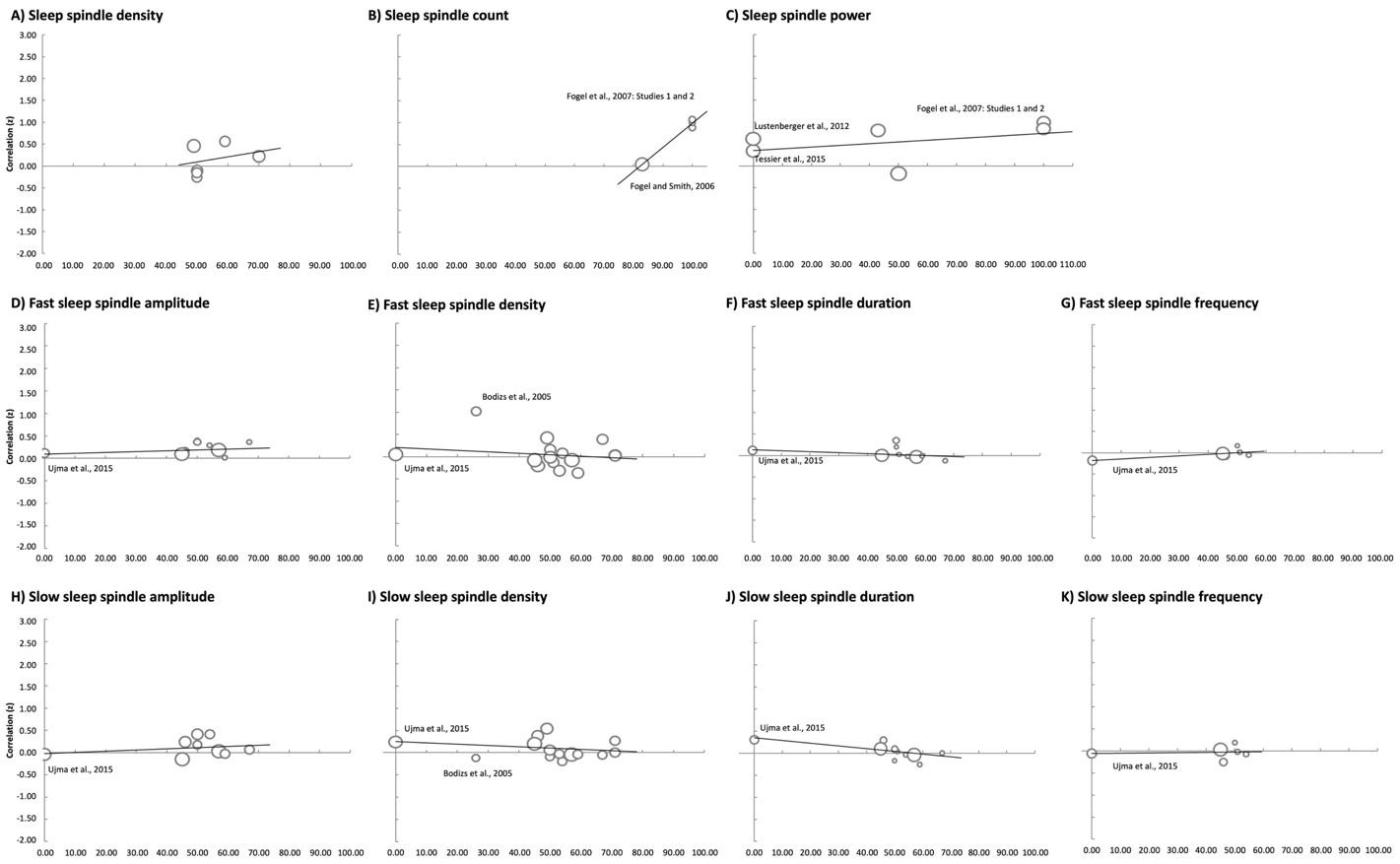
**

**Supplementary Table 40. Meta-regression analyses: Correlation between sleep spindles and cognitive ability moderated by the percentage of females.** Slope and CI of the predicted regression line between sleep spindle parameter and cognitive ability moderated by the percentage of females in the study. R-square gives the proportion of between-study variance explained by the moderator ‘female’. A positive slope coefficient (ß) indicates studies including female participants tended to show larger effect sizes than those with male participant. pz is the p-value for the slope coefficient.

| **Sleep Spindles** | **Slope** | **95% CI** | **p_z_** | **R^2^** |
| --- | --- | --- | --- | --- |
| **Density** | 0.01 | [-0.04, 0.06] | 0.559 | 9.11 |
| **Count** | 0.05 | [0, 0.10] | **0.0005** | 98.76 |
| **Sigma** | 0.004 | [-0.01, 0.01] | 0.468 | 14.76 |
| **Fast sleep spindles** | **Slope** | **95% CI** | **p_z_** | **R^2^** |
| **Amplitude** | 0.002 | [0, 0.01] | 0.422 | 10.88 |
| **Density** | -0.003 | [-0.01, 0.01] | 0.396 | 4.00 |
| **Duration** | -0.002 | [0, 0.01] | 0.312 | 14.00 |
| **Frequency** | 0.003 | [0, 0.01] | 0.190 | 67.18 |
| **Slow sleep spindles** | **Slope** | **95% CI** | **p_z_** | **R^2^** |
| **Amplitude** | 0.003 | [0, 0.01] | 0.483 | 7.14 |
| **Density** | -0.003 | [-0.01, 0] | 0.351 | 6.47 |
| **Duration** | -0.01 | [-0.01, 0] | **0.006** | 56.05 |
| **Frequency** | 0.0007 | [-0.01, 0.01] | 0.811 | 1.43 |

**References**

Anderer P, Gruber G, Parapatics S, Woertz M, Miazhynskaia T, Klosch G, Saletu B, Zeitlhofer J, Barbanoj MJ, Danker-Hopfe H, Himanen SL, Kemp B, Penzel T, Grozinger M, Kunz D, Rappelsberger P, Schlogl A, Dorffner G (2005) An E-health solution for automatic sleep classification according to Rechtschaffen and Kales: validation study of the Somnolyzer 24 x 7 utilizing the Siesta database. Neuropsychobiology 51:115-133.

Bodizs R, Kormendi J, Rigo P, Lazar AS (2009) The individual adjustment method of sleep spindle analysis: methodological improvements and roots in the fingerprint paradigm. J Neurosci Methods 178:205-213.

Crowley K, Trinder J, Kim Y, Carrington M, Colrain IM (2002) The effects of normal aging on sleep spindle and K-complex production. Clin Neurophysiol 113:1615-1622.

Della Monica C, Johnsen S, Atzori G, Groeger JA, Dijk DJ (2018) Rapid Eye Movement Sleep, Sleep Continuity and Slow Wave Sleep as Predictors of Cognition, Mood, and Subjective Sleep Quality in Healthy Men and Women, Aged 20-84 Years. Front Psychiatry 9:255.

Dement W, Kleitman N (1957) Cyclic variations in EEG during sleep and their relation to eye movements, body motility, and dreaming. Electroencephalogr Clin Neurophysiol 9:673-690.

Durlak J (2009) How to Select, Calculate, and Interpret Effect Sizes. Journal of Pediatric Psychology. March: 34(9):917-28.

Ehlers CL, Kupfer DJ (1997) Slow-wave sleep: do young adult men and women age differently? J Sleep Res 6:211-215.

Fang, Ray L, Owen AM, Fogel SM (2017) Simultaneous EEG-FMRI reveals spindle-related neural correlates of human intellectual abilities during NREM sleep. . Sleep Medicine 40:99.

Fisher RA (1921) On the "probable error" of a coefficient of correlation deduced from a small sample. Metron, 1, 3-32.

Fogel, Vien C, Karni A, Benali H, Carrier J, Doyon J (2017) Sleep spindles: a physiological marker of age-related changes in gray matter in brain regions supporting motor skill memory consolidation. Neurobiol Aging 49:154-164.

Gaillard JM, Tissot R (1973) Principles of automatic analysis of sleep records with a hybrid system. Comput Biomed Res 6:1-13.

Guazzelli M, Feinberg I, Aminoff M, Fein G, Floyd TC, Maggini C (1986) Sleep spindles in normal elderly: comparison with young adult patterns and relation to nocturnal awakening, cognitive function and brain atrophy. Electroencephalogr Clin Neurophysiol 63:526-539.

Hedges LV (1981) Distribution Theory for Glass's Estimator of Effect size and Related Estimators. Journal of Educational and Behavioral Statistics 6:107-128.

Huupponen E, Gomez-Herrero G, Saastamoinen A, Varri A, Hasan J, Himanen SL (2007) Development and comparison of four sleep spindle detection methods. Artif Intell Med 40:157-170.

Iber C, Ancoli-Israel S, Chesson AL, Quan SF (2007) The AASM manual for the scoring of sleep and associated events. Westchester, IL: American Academy of Sleep Medicine.

Klinzing JG, Kugler S, Soekadar SR, Rasch B, Born J, Diekelmann S (2018) Odor cueing during slow-wave sleep benefits memory independently of low cholinergic tone. Psychopharmacology (Berl) 235:291-299.

Mander BA, Santhanam S, Saletin JM, Walker MP (2011) Wake deterioration and sleep restoration of human learning. Curr Biol 21:R183-184.

Martin N, Lafortune M, Godbout J, Barakat M, Robillard R, Poirier G, Bastien C, Carrier J (2013) Topography of age-related changes in sleep spindles. Neurobiol Aging 34:468-476.

Mölle M, Bergmann TO, Marshall L, Born J (2011) Fast and slow spindles during the sleep slow oscillation: disparate coalescence and engagement in memory processing. Sleep 34:1411-1421.

Page MJ et al. (2021) The PRISMA 2020 statement: an updated guideline for reporting systematic reviews. Syst Rev 10:89.

Principe JC, Smith JR (1982) Sleep spindle characteristics as a function of age. Sleep 5:73-84.

Ray LB, Fogel SM, Smith CT, Peters KR (2010) Validating an automated sleep spindle detection algorithm using an individualized approach. J Sleep Res 19:374-378.

Ray LB, Sockeel S, Soon M, Bore A, Myhr A, Stojanoski B, Cusack R, Owen AM, Doyon J, Fogel SM (2015) Expert and crowd-sourced validation of an individualized sleep spindle detection method employing complex demodulation and individualized normalization. Front Hum Neurosci 9:507.

Rechtschaffen A, Kales A (1968) A manual of standardized terminology, techniques and scoring system for sleep stages of human subjects. Los Angeles: Brain Information Service, University of California.

Rethlefsen ML, Kirtley S, Waffenschmidt S, Ayala AP, Moher D, Page MJ, Koffel JB, Group P-S (2021) PRISMA-S: an extension to the PRISMA Statement for Reporting Literature Searches in Systematic Reviews. Syst Rev 10:39.

Schabus M, Dang-Vu TT, Albouy G, Balteau E, Boly M, Carrier J, Darsaud A, Degueldre C, Desseilles M, Gais S, Phillips C, Rauchs G, Schnakers C, Sterpenich V, Vandewalle G, Luxen A, Maquet P (2007) Hemodynamic cerebral correlates of sleep spindles during human non-rapid eye movement sleep. Proc Natl Acad Sci U S A 104:13164-13169.

Staresina BP, Bergmann TO, Bonnefond M, van der Meij R, Jensen O, Deuker L, Elger CE, Axmacher N, Fell J (2015) Hierarchical nesting of slow oscillations, spindles and ripples in the human hippocampus during sleep. Nat Neurosci 18:1679-1686.

Ujma PP (2018) Sleep spindles and general cognitive ability – A meta-analysis. Sleep Spindles & Cortical Up States.

Ujma PP, Bodizs R, Gombos F, Stintzing J, Konrad BN, Genzel L, Steiger A, Dresler M (2015) Nap sleep spindle correlates of intelligence. Sci Rep 5:17159.
